# The York County Hospital: Inquiry by Special Commission

**Published:** 1913-04-26

**Authors:** 


### THE YORK COUNTY HOSPITAL.

### Inquiry by Special Commission.

### FULL VERBATIM REPORT OF THE PROCEEDINGS.

The inquiry into the charges against the administration of the York County Hospital, made by Drs. Macqueen and Shepherd, which appeared in The Hospital of March 15, was opened at the hospital on Friday, April 18, by Sir Cooper Perry, M.D., of Guy's Hospital, the Commissioner appointed on the nomination of the President of the Royal College of Surgeons, with Mr. Basil Watson as Legal Assessor.

Drs. Macqueen and Shepherd were represented by Mr. Arnold Baker, Barrister, and Mr. Alfred Procter appeared on behalf of the hospital.

Sir Cooper Perry said : By the desire of the Governors of the County Hospital at York I am to hold an inquiry with the assistance of a legal assessor, Mr. Basil Watson, into the charges made against the hospital and published in The Hospital newspaper on the 15th March, and I have to furnish the Committee with a report on the result of my investigations. The conduct of the inquiry is left to me, and I understand that the Governors of the hospital propose to publish the report in due course. We thought it desirable that the press should have an opportunity of being present at our inquiry, and we endeavoured, through Mr. F. Neden (Secretary of the hospital), to secure the attendance of Dr. Shepherd and Dr. Macqueen. Shortly after I came into the hospital this morning this letter was handed to me from a firm of solicitors, who say they have delivered a brief to Mr. F. S. Arnold Baker to appear at the inquiry as counsel for Drs. Macqueen and Shepherd, adding " as from an intimation that Sir Cooper Perry will be assisted by a legal assessor, we take it that the inquiry will be conducted in accordance with the usual practice in legal proceedings." At present Mr. Arnold Baker is not here. The course that I was proposing to adopt was to take these charges in the order in which they appeared in The Hospital newspaper of March 15, but as a first witness I will ask Mr. Neden to give me some information as to the general management and constitution of the hospital.

Mr. Neden : There is no objection to one of our Committee being present?

The Commissioner : Oh, dear, no.

### EXAMINATION OF WITNESSES.

Mr. Neden, the Secretary, examined : I understand your questions are in regard to the general management of the hospital?

The Commissioner : The constitution of the hospital.

Mr. Neden : The constitution of the hospital consists in the first place of a Court of Trustees whose particular duty is to look after the investments, and also, I may say, the hospital is managed under rules which must be adhered to, and no rule with regard to the management of the hospital can be altered, and no new rule made, without the sanction of the Court of Trustees. This Court meets quarterly. Then, in addition, there is a Court of Governors, which consists of all subscribers of two guineas and upwards. This Court also meets quarterly.

Mr. Arnold Baker then entered the room.

The Commissioner : At present we have got so far as to ask the Secretary of the hospital to give me some sketch of its constitution. We have not dealt with any of the charges.

Mr. Arnold Baker : I am so sorry we were a little late.

The Commissioner (to Mr. Neden) : You have told us that there is a Court of Governors which meets quarterly, and to be a Governor you qualify by a subscription of two guineas a year. Now will you please explain what the House Committee does?

Mr. Neden : The actual management of the hospital rests with the House Committee. They are responsible for the whole of the administration, and, in fact, the actual conduct of the hospital rests with this Committee. They meet on the 2nd and 4th Tuesday in each month, practically twice a month, and, of course, their duties are to appoint all the paid officers, and at their meetings the Medical Board is represented by two members who are elected by the Medical Board annually. The only officer present is the Secretary. The Matron or any other paid official is not asked into the Board Room except under special circumstances. At every meeting, however, the Matron sends in her book, in which she reports anything which she thinks ought to be brought to the notice of the Committee—in case, for instance, of the engagement of members of the nursing staff, which must be confirmed by the House Committee. By nursing staff I particularly refer to the sisters. She also reports any case of illness in the staff.

The Commissioner : Then the Matron does not attend meetings of the House Committee? She sends her journal?

Mr. Neden : Yes.

The Commissioner : Is there a similar arrangement as regards the Residents?

Mr. Neden : No, Sir.

The Commissioner : They do not attend the House Committee?

Mr. Neden : No.

The Commissioner : And they have no diary or journal which is presented?

Mr. Neden : No.

The Commissioner : They neither appear or present any journal indicating any items which they may desire to bring to the notice of the Committee?

Mr. Neden : No.

The Commissioner : There is no arrangement of that kind?

Mr. Neden : The only medium of communication between the Residents and Managing Committee is by letter sent to the Secretary or to the Chairman of the Committee.

The Commissioner : The reason I ask the question is that I notice most of the communications are by letter?

Mr. Neden : Yes.

The Commissioner : There is another point which is not quite clear to me, and that is the relation of the Medical Board to the House Committee?

Mr. Neden : Yes, of course the Medical Board——

The Commissioner : I understand the medical staff have representatives on the House Committee; I think you said two members?

Mr. Neden : Yes.

The Commissioner : But what functions has the Medical Board independent of the House Committee?

Mr. Neden : Their principal duty is to consider questions which affect the professional and the medical or surgical side of the house, and it is the custom of the House Committee to refer to them any question which they think the Medical Board more competent to deal with. For instance, in the way of buildings, and the question of addition or anything like that—anything which they think the Medical Board would be more competent to judge of than the House Committee.

Mr. Procter : They appoint two members to the House Committee.

The Commissioner : That I already understand. The Medical Board has, I understand it, no independent executive functions, but they may be called in to advise, but they are not an executive; the executive is the House Committee.

Mr. Neden : No.

The Commissioner : The reason I ask the question is that the Residents state they quote from some rules in which they say the house surgeon and house physician shall be appointed and be removable by the House Committee and members of the honorary and surgical staff.

Mr. Neden : Yes.

The Commissioner : What does that exactly mean?

Mr. Neden : Perhaps I had better explain the formula in which the Residents are appointed. After the advertisements are put in, all the applications are put before the Medical Board, and then the Medical Board select as a rule three candidates to appear before a meeting of the House Committee, which the Medical Board are invited to attend, and the members of the Medical Board have votes on the question of the election of the resident staff.

The Commissioner : As a matter of fact were members of the Medical Board summoned to a meeting of the House Committee at which it was decided to remove these gentlemen?

Mr. Neden : No, Sir.

The Commissioner : I think, Mr. Neden, you have given all the information I want; perhaps you will supply me with a copy of the printed rules.

### THE CHARGES TAKEN IN ORDER.

The Commissioner : We are proposing to take the charges in the order in which they appeared in The Hospital, and as far as possible dealing with them fully. (See The Hospital, March 15, 1913, pp. 645-648.)

### VIOLENCE TO CHILD PATIENT AGED TWO YEARS.

First. On page 645, under the heading of "Violence and Mischarting," there is the statement, "On October 14, 1912, we made reports to the Medical Board regarding some of the existing conditions. The reports were to this effect :—That a child in the children's ward was discovered by us with suspicious marks upon it, which we attributed to violence. Inquiries from the matron and nurses brought no satisfactory explanation of the marks."

The same question is dealt with in a letter dated October 13 (so that there seems to be a day wrong in the statement) from Dr. Shepherd. He says this to the Medical Board :—"I wish to call your attention to the following *re* Jimmy Merrick, at present a patient in the Victoria Ward. The above child is now convalescent from an empyema of the right side and almost fit to go home. This morning, Sunday, 13th, I visited the ward and asked that the child's dressing be removed as I wished to examine him, not having done so for some time, as everything was going on quite well, and sister reported that the sinus has . . . . almost closed. I found everything as reported, but noticed on the left side of the chest and extending towards mid-axillary line what appeared to me marks which might well have been bruises. As regards their age I should feel inclined to state from five to seven days old. As the child appeared to be very frightened and not what he had been on a previous occasion, I sought to make further inquiries from the nurse in charge, the sister being away for the week-end. Nurse was confused and did not seem to like my inquiries. She said she knew about it but would rather not say at present. I had Dr. Macqueen sent for to examine the child, which he did, and was present while I questioned the nurse. I telephoned for Dr. Gayner, and said I should like him to see his case, but meanwhile I thought I might as well see the Matron. For some reason or other, perhaps best explained by herself, Matron was inclined to argue and make various statements—amongst others, that I must have had information from some source, and that, furthermore, she thought it not proper to look into such things when the sister was away, saying it was underhand. Moreover, she deliberately stated that I must have been making inquiries regarding the conduct of the sister in the ward, otherwise I should not have noticed anything the matter with the child. I look upon this as a gross insult, and demanded an apology. I think that any Matron who makes such statements oversteps her position. What difference my inquiries into the marks made to Matron, whether sister is away or not, puzzles me. There were other things said, but I must ask the Medical Board to have the matter fully investigated, and until this is done I cannot see my way to see any case in the Victoria Ward, along with anyone who is in any way connected with this, nor can I have any dealing with the Matron until she apologises."

The Commissioner : Have you anything to add to that, Dr. Shepherd?

Dr. Shepherd : Nothing further than what is stated; it is pretty fully set out.

The Commissioner : I think so; thank you. What have you, Mr. Procter, to say with regard to that?

Mr. Procter : I think, Sir, what has been said had better come from the medical officers who are in charge of that case; there is a report made.

The Commissioner : I have the report of the medical officers.

Mr. Procter : You will see that the matter was dealt with by them on October 16.

The Commissioner : Yes.

### THE REPORT OF THE MEDICAL OFFICERS.

Mr. Procter : And that (indicating their report) is their reply.

The Commissioner read this report, as follows :—

Special meetings of the Medical Board, held 11.30 a.m. and 8.30 p.m., to inquire into breaches of discipline in the Victoria Ward and other matters.

Papers were put in by Drs. Macqueen and Shepherd, Miss Tute, and the Sister of Victoria Ward at that time. Verbal reports were made by Messrs. Gayner and Gostling, Drs. Evelyn and Hughes.

Drs. Macqueen and Shepherd, Miss Tute, Sister Victoria, Nurses Sharp and Firth, appeared and gave evidence.

Sister Victoria reported (1) that on October 6 she did strike a child in Victoria Ward severely and had not reported this occurrence.

The Commissioner : That is all that deals with this particular point.

Then it proceeds : "Having regard to the above the Medical Board decided to recommend Miss Tute to accept the resignation and forward same to the House Committee."

The Commissioner (to Mr. Neden) : Does a sister resign to the Matron?

Mr. Neden : She hands in her resignation to the Matron, and it is confirmed or otherwise by the House Committee. I may say she has the power to suspend any member of the staff.

The Commissioner : So that the result of the inquiry was that the resignation of the sister was accepted?

Mr. Neden : That is so. I should like you to hear the evidence of the Senior Medical Officer with regard to this particular case.

The Commissioner : I should be very glad.

Dr. Evelyn, Senior Physician, called.

The Commissioner : Was the patient under your care?

Dr. Evelyn : I was acting as Chairman of the Medical Board when this report was prepared.

The Commissioner : Do you wish to amplify this report?

The report is initialled by six members of the staff, I see.

Dr. Evelyn: I may say that the question of the conduct of the Sister was thoroughly gone into by the Medical Board at these two meetings, which were very lengthy meetings.

The Commissioner: But the conclusion to which the Board came was that her resignation should be accepted. I don't suppose that you wish to carry the matter further?

Dr. Evelyn: No: we did not wish to carry the matter any further; we thought that the Sister having given in her resignation was quite sufficient.

Mr. Procter: But you did make an examination ?

Dr. Evelyn: Oh, yes; I may say I was asked by Dr. Shepherd to see this case. When I came down to the hospital I was taken to the bedside of the patient. The patient was uncovered, and I was shown the marks on the child, and, looking at the child's back, I said I was sorry, but I did not see anything on the child; "Where are the marks?" and there was a small mark which was pointed out to me in the back on the left side. A small superficial mark due very probably to the wreathing of the bandage which had been round the child's body, and that was all I could find or see.

Mr. Procter: As to the position where the child was said to have been struck?

Dr. Evelyn: I believe the child was said to have been struck on the right shoulder, but it was the left portion of the back of the chest which was pointed to me as being the place where the injury was to be seen.

Mr. Procter: The injury from a striking?

Dr. Evelyn: From a striking.

### CHILD STRUCK SEVERELY.

The Commissioner: The report of the Medical Board says that the Sister said that on October 6 she had struck the child in the Victoria Ward severely and had not reported the occurrence.

Dr. Evelyn: Yes.

The Commissioner: And the Board accepted that statement as true, I suppose?

Dr. Evelyn: Yes.

The Commissioner: So that while it may be a fact that you were unable to identify the particular marks which were seen by Dr. Shepherd, the Board were satisfied that the Sister had struck the child and accepted her resignation accordingly. Is that not so?

Dr. Evelyn: Yes.

The Commissioner: Does that put it fairly?

Dr. Evelyn: Quite so; but the fact that I on seeing the child could not see any signs of any bruising went to show that the blow could not have been a very severe one. It is much to be regretted that the Sister should ever have forgotten herself so far as to hit a child at all, let alone a child that was ill. The Sister was very upset about it, and recognised that she had done a very wrong thing.

The Commissioner: Yes.

Mr. Procter: The point is that the mark that you looked at which was shown as the result of the blow was not the result of a blow at all.

Dr. Evelyn: Absolutely not.

Mr. Arnold Baker: Do you think that the decision of the Board that the Sister should resign justifies the complaint made by Drs. Macqueen and Shepherd?

Dr. Evelyn: I don't quite follow.

Mr. Baker: Do you consider that the complaint made by Drs. Shepherd and Macqueen was justified by the decision arrived at by the Board?

Dr. Evelyn: Undoubtedly the Sister resigned as a result of the complaint.

Mr. Baker: But I am asking whether you think the result of that decision justified the action of the two gentlemen whom I represent in making that complaint?

Dr. Evelyn: So far as that complaint was concerned—yes.

Mr. Basil Watson: I don't quite understand? Do you suggest that this lady resigned and made the statement that she did severely strike the child, when as a fact she did not do so?

Dr. Evelyn: No.

The Commissioner: It is doubtful whether the particular place or marks shown to him were produced on this occasion as recorded by Drs. Shepherd and Macqueen.

Mr. Baker: I thought it hardly worth while that I should ask you. You do not deny that Dr. Shepherd and Dr. Macqueen did see marks?

Dr. Evelyn: I have not the faintest idea whether they saw them or not.

Mr. Baker: Do you think it likely?

Dr. Evelyn: What I say is that I did not see the marks.

Mr. Baker: You won't say that they made this complaint without seeing the marks?

Dr. Evelyn: I do not know at all; but I certainly did not see the marks.

Mr. Baker: I mean, do you wish to suggest to the Commissioner that they really made this complaint without being satisfied that the child had been struck?

Dr. Evelyn: They must, I presume, have seen some marks of some kind, or have heard that the child had been hit.

Mr. Baker: Now that we know that the child was struck, have you any doubt that they did in fact see the marks?

Dr. Evelyn: I could not say at all whether they saw any marks.

The Commissioner: Perhaps you cannot answer this question; it may be right that I should address it to the Matron. Do you know whether Sister Victoria or the Matron had any knowledge that the child had been struck?

Dr. Evelyn: I have no knowledge of that.

### THE ASSESSOR INTERVENES.

The Legal Assessor: May I ask you? Supposing a child should be known as extremely naughty, what is the right course for a nurse to pursue?

Dr. Evelyn: The nurse would have to remain with the child until she had got the child into a reasonable frame of mind, or she would have to take means of restraining the child.

The Legal Assessor: Supposing the child, for instance, is suffering from some very slight thing, and a question of what we call a spank would stop the child—the same sort of spank as administered to one's own children—would the nurse have the right to do it without communication with the doctor?

Dr. Evelyn: I do not think that a nurse has any right whatever to hit a child.

Mr. Gayner, Hon. Medical Officer, called.

The Commissioner: You have heard the report of the Medical Board, and, of course, you are familiar with it?

Mr. Gayner: Yes. This paper [produced] I dictated to my amanuensis—as a matter of fact, my wife—day by day as the matter proceeded. It is not a note taken on the spot, but as soon as I returned home from the hospital, and I think if you will read it——

The Commissioner: Does it bear upon this particular point?

Mr. Gayner: It deals with nothing else.

The Commissioner read the following statement:—

### MR. GAYNER'S STATEMENT.

"Sunday, October 13, 1912.

"10.30. Report on telephone by Dr. Shepherd that a patient of mine in Victoria shows marks on chest wall suggestive of having been struck with fingers, and that the nurse in charge of case refuses to answer questions in reference to the same. Also a request for me to proceed at once to the hospital. At the York County Hospital Dr. Shepherd explained that marks had been observed by him during course of examination made with a view to discharge of patient. Further, that in reporting matter to Miss Tute (the Matron) she had accused him of having derived his information from gossip in the hospital.

"In Victoria an examination of the left side of the child's chest showed suggestion of a bluish mark about two inches in length on left side of the chest wall. The mark was apparently less than half an inch across and extended from the mid-axillary line upwards and backwards. The nurse in charge of the case, questioned whether she had observed anything in this region during the past week, answered, after delay, 'I am not obliged to answer that question, am I?' and I assented to the view that she could not be forced to answer the question. Leaving the ward, I discussed the matter with the Matron, pointing out that Dr. Shepherd was greatly upset at her charge that he had been led to discover the matter by tittle-tattle in the hospital, and urged her either to withdraw the charge or to put such matter in writing as would tend to substantiate it. Further, I reported the result of my interview with the charge nurse in Victoria, and asked that the nurse might be called to the Matron's room for an immediate opportunity to explain her action. This, after some delay, being done, I, in the presence of the Matron, again put the question: Had she during the past week observed anything on the child's chest? The nurse, refusing to answer the question, withdrew, and I expressed strongly to the Matron the view that a nurse so disobedient was entirely unsuitable for work in the hospital, and that the matter would have to be reported to the House Committee.

"Later I advised Dr. Shepherd to make a note of the matter, and to get Dr. Evelyn to see the patient. I telephoned to Dr. Evelyn's house, and, going down to Victoria, I saw the charge nurse alone, and asked her if she had anything to say. She replied, 'No.' There was one note I missed which was also dated the same day, viz., 'The mark was not so obvious that I should have discovered its presence if my attention had not been directed to the question.' We now come to Monday, October 14.

"At York County Hospital Miss Tute reports that Nurse Sharp has informed her that she saw Sister Victoria strike the patient a week ago, and had seen the bruises during the last week.

"Miss Tute reported Nurse Sharp was still in charge of the ward. I represented strongly to her that I should object to go round with a nurse who had refused to answer questions as to the health of a patient under her care as outlined above, and I urged Miss Tute to remove the nurse to another ward. This having been done, I was met by the nurse in the hall, and, in response to her request, discussed the matter with her. I gathered from her that until her removal from the ward she had not been aware that her refusal to supply information to the officers responsible for the case could be regarded as a very grave offence against hospital discipline, and I am inclined to think that the motive of her action was a generous desire not to report evil of her Ward Sister during the Sister's absence.

"Age of the patient two years on admission. Sister Victoria reported due back this afternoon."

Mr. Gayner: What I wish to point out is that the mark which my attention had been directed to was here [indicating the left shoulder]. It was a bluish, mottled mark, not yellow; it was such a mottling as would be seen on a child specially suffering from a chest complaint. It was clearly shown from the subsequent inquiry by the Sister and Nurse Sharp that the smack was here [on the right shoulder]. There is no question about it; the smack was on the right side, and the imaginary mark was on the left. The importance of that is that it affects the value of the evidence both of Dr. Macqueen and Dr. Shepherd. They both say that they saw the marks of injury on the left shoulder.

### WHEN AND WHERE THE CHILD WAS STRUCK.

Mr. Baker: Do you know, Mr. Gayner, this child was suffering from a chest complaint?

Mr. Gayner: Yes.

Mr. Baker: Do you know which side of the chest was particularly affected?

Mr. Gayner: The right.

Mr. Baker: You know that?

Mr. Gayner: Yes.

Mr. Baker: And the mark was on the other side of the chest?

Mr. Gayner: Yes.

Mr. Baker: I understood by one of your first explanations that you suggested that the child had been struck opposite to the side upon which it was suffering the pain. Just at the end you said it probably had been struck on the affected side, because the nurse told you. Are you making any definite suggestion.

Mr. Gayner: I am definitely suggesting that the mark here [left side], to which my attention was called by Dr. Shepherd, which is referred to in his paper, was not due in any way to a blow.

Mr. Baker: You are suggesting that Dr. Shepherd and Dr. Macqueen made this allegation that the child had been struck on evidence which was totally insufficient?

Mr. Gayner: Which was totally erroneous.

Mr. Baker: And, in spite of that, when this matter was inquired into, the Board recommended that the nurse should be dismissed?

Mr. Gayner: Yes, because she admitted striking the child.

The Commissioner: Shall I go on with your notes?

Mr. Procter: I was hoping that that would be done without mentioning of names; that it would be sufficient to say "the sister of the ward."

The Commissioner: Perhaps the press will take note that the Sister has handed in her resignation, and it was accepted; therefore in any notice that may appear, it will be better to say "the Sister of Victoria Ward at that time," rather than mentioning her name.

Mr. Procter: Is it not desirable that that, as far as possible, should be the general rule?

Mr. Baker: About the naughty child: do you know exactly when this child was struck? Were you told by the nurse that it was during the time while the wound was being drèssed?

Mr. Gayner: Yes, I was told that. We have somewhere, I think, minutes of the evidence which was taken at the inquiry.

The Commissioner: May I go on with your notes? [Reading] "October 15, 1912. York County Hospital. Interview with Miss Tute, who reports Sister Victoria has returned to the hospital, and that she has admitted to Miss Tute (1) striking the child; (2) observing signs of the blow on subsequent days; (3) failure to report the matter to Miss Tute or Dr. Shepherd. Miss Tute further informed me Dr. Macqueen had objected (1) to Sister Victoria being on duty; (2) to Nurse —— remaining on duty in the ward, on the ground that the latter had refused to go round with Sister Victoria, and that after consultation with Mr. Gostling she had temporarily removed Sister Victoria from duty. I again urged on Miss Tute the importance of drawing up a written record of the matter."

The Commissioner: Thank you very much for that note. I think it is quite plain that what the contention is is that the mark that Drs. Shepherd and Macqueen observed was not the mark which was inflicted by the Sister. Is not that so?

Mr. Gayner: Yes.

The Commissioner: But the blow was seen by Nurse Sharp.

Mr. Baker: If that is persisted in, I think I am entitled to ask that Nurse Sharp shall come here.

The Commissioner: Is Nurse Sharp available?

Mr. Neden: Yes.

The Commissioner: Nurse Sharp should appear.

The Legal Assessor: I do not see why.

Mr. Baker: If there is any intimation which comes from you or the Commissioner, I do not attach any importance to her evidence; but there is at present an allegation against my clients. If I receive any intimation from you that you attach no importance to that, then I do not want to call Nurse Sharp.

The Commissioner: Do you wish the nurse called?

Mr. Baker: No; under these circumstances——

Mr. Procter: May I say that we do attach importance to this question as having an important bearing on the whole of the charges made by these two gentlemen? We have endeavoured to show that with regard to these marks there has been inaccuracy, and we attach importance to that as regards the whole of their charges.

The Commissioner: I think we had better have the nurse called.

Mr. Baker: The point of the allegation is not so much as to this specific mark, but they found marks and had their suspicions aroused as to how they were caused, and then, after their inquiries, found the child was struck.

Nurse Sharp was then called.

The Commissioner: You were the nurse in Victoria Ward when the incident occurred of the striking of the child by the Sister who has now left?

Nurse Sharp: Yes.

The Commissioner: And you observed certain marks on the child's body?

Mr. Baker: On which side was the child struck?

Nurse Sharp: On the left side, more or less in the centre, slightly to the left, by the palm of the hand.

Mr. Baker: Do you know if it was struck more than once?

Nurse Sharp: Yes.

Mr. Baker: How many times?

Nurse Sharp: I don't know; but several times.

Mr. Baker: Was the child struck while it was being dressed?

Nurse Sharp: Yes.

The Commissioner (to Mr. Procter): Do you wish to carry that any further?

Mr. Procter: I would like to know from Dr. Shepherd where he got that information from?

Dr. Shepherd: I got it from my own eyes.

Mr. Procter: When was that?

Dr. Shepherd: I saw it on Sunday morning, October 13, while examining the child's chest.

Mr. Procter: When did you want Dr. Evelyn to see it?

Dr. Shepherd: The same day.

Mr. Procter: What sort of marks do you say you saw?

Dr. Shepherd: I saw marks on the skin, which were given the appearance of parchment as if the capillaries had been ruptured, and that glossed appearance which is characteristic of such marks.

Mr. Procter: Did you point those out to Dr. Evelyn?

Dr. Shepherd: Yes.

Mr. Procter: You have heard his evidence?

Dr. Shepherd: Yes.

Mr. Procter: Do you say it is not true?

Dr. Shepherd: I would not like to make the statement that it is not true. We are none of us——

The Commissioner: We are none of us infallible.

Dr. Shepherd: I do not wish to make any allegation.

Mr. Procter: You speak of finger-marks; do you mean five finger-marks.

Dr. Shepherd: No, I cannot say exactly whether there were three or four; there were certainly three, but four I cannot say for.

Mr. Procter: Three definite marks?

Dr. Shepherd: Yes.

Mr. Procter: Were they in the position in which Nurse Sharp indicated?

Dr. Shepherd: Yes.

Mr. Procter: Which side of the child do you think the Sister was when she struck it?

Dr. Shepherd: She would, I expect, be on the right side dressing the chest.

The Commissioner: The appearance is compatible with that?

Dr. Shepherd: Yes.

Mr. Procter: And that Sunday morning was the first time you saw these marks?

Dr. Shepherd: Yes.

Mr. Procter: Did you form any opinion as to how long since they had been caused?

Dr. Shepherd: I have already said five or seven days.

Mr. Procter: The child was under your care during the whole of that time.

Dr. Shepherd: It was.

Mr. Procter: Will you explain why you didn't see the marks before?

Dr. Shepherd: It was a case which it was not necessary to examine daily. The child was out of danger, and only the sinus remained.

Mr. Procter: It was not necessary to see it during those seven days?

Dr. Shepherd: No; it was out of danger, and had been for five or six weeks; the case, as a matter of fact, was in before I came to the hospital.

Mr. Procter: How was it you came to examine it on the 13th?

Dr. Shepherd: Sunday, being the quietest day. I had meant to examine it previously, but my duties as a casualty officer made it impossible; in fact, many a day I went to the ward and had immediately to leave it.

Mr. Procter: That was the first time you made any examination?

Dr. Shepherd: Yes, for over a week.

Mr. Procter: Do you say that was the first intimation you had of these bruises?

Dr. Shepherd: Yes, it was.

Mr. Procter: Had you not heard anything in the hospital?

Dr. Shepherd: I had not.

Mr. Procter: Not from anybody?

Dr. Shepherd: No; after I had found them out I heard more promiscuously if you care to put it that way. I was more inclined to think that they were bruises and there was something the matter with the child more than ordinary, for the simple reason that while I was making the examination of the child it was in a very frightened condition, not at all as it had been previously.

Mr. Procter: How old was the child?

The Commissioner: Two years.

Mr. Procter: Were these bruises pointed out to you by a Sister or nurse in the ward?

Dr. Shepherd: They were not.

Mr. Procter: Your attention was not called to them?

Dr. Shepherd: My attention was miscalled from them.

Mr. Procter: You still adhere to your statement that there were finger-marks, after hearing Dr. Evelyn's evidence?

Dr. Shepherd: I do.

### QUALIFICATIONS OF DRS. SHEPHERD AND MACQUEEN.

The Legal Assessor: On October of last year, 1912, how many years' hospital experience had you had?

Dr. Shepherd: I graduated in July of last year, but I had had three years' experience in the wards of the Royal Infirmary, Edinburgh, previous.

Mr. Procter: You had graduated about four months?

The Commissioner: This was the first resident appointment you had had?

Dr. Shepherd: After my degree, but not my first experience.

The Commissioner: Was it your first resident appointment, Dr. Macqueen?

Dr. Macqueen: No.

The Legal Assessor: How old were you in October last year?

Dr. Shepherd: Twenty-three.

The Legal Assessor: What are your degrees?

Dr. Shepherd: M.B., Ch.B. Edinburgh. I graduated with distinction in medicine and midwifery.

Mr. Procter: I only wish, if I may, to point this out. That one of the serious charges is that the complaints made were not dealt with. At any rate, so far as this charge is concerned, it was dealt with in two or three days by the Medical Board

The Commissioner: Can we see the Matron with regard to this case?

Dr. Macqueen was recalled and asked: Did you see this child at the same time as Dr. Shepherd?

Dr. Macqueen: Yes.

The Commissioner: On Sunday morning?

Dr. Macqueen: Yes.

The Commissioner: How many marks did you see?

Dr. Macqueen: I saw three marks.

The Commissioner: Did you go round with Dr. Evelyn and Dr. Shepherd?

Dr. Macqueen: No.

The Legal Assessor: When did you graduate?

Dr. Macqueen : In July 1911.

The Commissioner : I suppose you had had three years before that?

Dr. Macqueen : Yes.

The Commissioner : And graduated just over a year?

Dr. Macqueen : Yes. I was casualty surgeon, assistant house surgeon, and ophthalmic house surgeon at Leicester Royal Infirmary.

The Commissioner : How long were you there?

Dr. Macqueen : Between eight and nine months.

Mr. Baker : Your degrees were Bachelor of Medicine and Bachelor of Surgery.

Dr. Macqueen : Yes.

Mr. Proctor : How old were you in October?

Dr. Macqueen : I am 22.

Miss Tute, the Matron, was then called.

### THE MATRON EXAMINED.

The Commissioner : I have to ask you some questions upon the letters addressed by Dr. Shepherd to the Committee relating to the case of this child in the Victoria Ward struck by the Sister? I need not read the first part of it, which does not concern you. We have already gone into it, but he goes on to state : "I telephoned for Dr. Gayner and said I should like him to see his case, but meanwhile I thought I might as well see the Matron. For some reason or other, perhaps best explained by herself, the Matron was inclined to argue and make various statements, amongst others that I must have had information from some source, and that furthermore she thought it not proper to look into such things when the Sister was away, saying it was underhand. Moreover, she deliberately stated that I must have been making inquiries regarding the conduct of the Sister in the ward, otherwise I should not have noticed anything the matter with the child. I look upon this as a gross insult, and demand an apology; I think that any matron who makes such statements oversteps her position. What difference my inquiries into the marks made to Matron, whether Sister is away or not, puzzles me. There were other things said, but I must ask the Medical Board to have the matter fully investigated, and until this is done I cannot see my way to see any case in the Victoria Ward, along with anyone who is in any way connected with this, nor can I have any dealing with the Matron until she apologises."

The Commissioner : We have had it in evidence from Dr. Shepherd, Matron, that he had not got this information from any nurse or any other source, but it was the result of his own investigation of the child's condition, and he explains that he was looking at the child specially because it was a Sunday, which was a quiet day, and that while seeing the case dressed he noticed these marks as to which we have had a good deal of evidence. What I rather want to know from you is, have you any reason to suppose that Dr. Shepherd did get information from any nurse or patient, although hardly a patient because it was a child's ward?

The Matron : A nurse said she had been questioned by the doctor.

The Commissioner : But the nurse was questioned by the doctor, as it appears to us, after the injury was discovered?

The Matron : I understood she had been questioned as soon as she had gone to the ward.

The Commissioner : Which nurse?

The Matron : Nurse Sharp.

The Commissioner : You don't yourself know that the Sister had been striking the child?

The Matron : No; I did not know that a child had been struck. It was my first intimation.

The Commissioner : When did this nurse tell you that Dr. Shepherd had been making inquiries?

The Matron : It was after Dr. Shepherd had left the ward that he had spoken to me. It was the second time I saw him when I spoke to him about getting the information. It was not the first time that I saw him.

The Commissioner : But it was all in relation to this case?

The Matron : Yes.

The Commissioner : You are quite clear that the inquiries were made before the marks were discovered, and not after the discovery?

The Matron : It was after the doctor left the ward, and I was asking her about it and she said she had been questioned.

The Commissioner : Is Dr. Shepherd's statement right that you did accuse him of having obtained information from some source?

The Matron : What I said to him was that I thought he had acted on information rather than observation. It was after I had seen the child and I could not see anything.

The Commissioner : So it really rests with Nurse Sharp, who told us where she got that information to the best of her ability?

The Matron : I did not inquire into that deeply then; it was not at my first interview with Dr. Shepherd that I mentioned it.

Mr. Proctor : You say you did examine the child?

The Matron : Yes.

Mr. Proctor : Was that on Sunday morning?

The Matron : Yes; I came into the ward when the doctor was there and met him at the door. He said he wanted to see one of the nurses.

Mr. Procter : Which doctor?

The Matron : Dr. Shepherd.

Mr. Procter : Did he ask you to examine the child?

The Matron : Yes; I think so. I naturally went to see it.

Mr. Procter : Did you examine the child?

The Matron : Yes.

Mr. Procter : Did you find any marks?

The Matron : I did not notice anything, and then I suggested that the Honorary should be communicated with.

Mr. Procter : It is said that there were marks on the left axilla.

The Commissioner : The evidence of the Honorary is that the child was struck on the right. The nurse says that she saw the blows struck on the left. You saw no marks anywhere?

The Matron : No.

Mr. Procter : You examined the child's back?

The Matron : Yes.

Mr. Procter : And saw no marks at all?

The Matron : No.

Nurse Sharp was then recalled.

The Commissioner : We have to ask you just another question. According to Dr. Shepherd's letter the Matron accused him of having obtained information from outside sources as to the blows to the child. I have asked the Matron what proof she had of that, and she says that you told her that inquiries had been made by Dr. Shepherd as to whether anything had happened to the child, before, I mean, the marks were actually discovered by Dr. Shepherd himself. Is that so or not?

Nurse Sharp : No, I don't remember saying so at all.

Mr. Procter : Did you speak of anything else beside these particular marks on this particular child?

The Commissioner : I don't quite know about that. A nurse may speak to a doctor about various things.

Mr. Procter : Were inquiries made from you by Dr. Shepherd about other things?

The Commissioner : I should think that carries it rather far. No, Nurse, I don't think you need answer that question. (To Mr. Procter) : Does that conclude that incident so far as you are concerned?

Mr. Procter : I think so.

### THE CHARGE OF MIS-CHARTING.

The Commissioner: The next paragraph is this:

"Also in the same ward the temperature of a case had been wilfully mis-charted. . . . Complaint had been made by us to the Matron previously about similar occurrences in the same ward, and no notice had been taken of it."

The letter which bears upon it is this: It is October 14, 1912, a letter from Dr. Macqueen:—

"To the Medical Board:

"Gentlemen,—I wish to call your attention to the case of Margaret Mahan in Victoria Ward—one of Dr. Micklethwaite's cases. One evening as I was doing my rounds I noticed this child's chart, and found that on October 1 her temperature had been charted at 101.6. This had been scratched out and re-charted as 100.6. On asking the staff nurse why such a mess was made of the chart, I was informed that she, on that particular day, had taken the temperature herself, and that it was 101.6, but that the sister of the ward, without herself taking the temperature, had erased this and charted it as 100.6.

"I have on a previous occasion laid a complaint before the Matron of similar occurrence in the same ward, and as it has again happened I think it is time the matter was looked into by the Medical Board. At the same time may I point out that on many of the charts in the same ward there will be found no record of pulses or respirations except on the day of admission, even though the case may have been in the ward for several weeks? One case in particular in which I think the pulse is of importance—namely, a case of osteomyelitis of the arm, which has not had the pulse or respiration recorded for seven weeks. In this child I had to open another abscess yesterday."

What have you to say about that, Mr. Procter?

Mr. Procter: That also, I think, has been investigated. Well, it does not indicate much, because it was all we had to deal with.

Mr. Baker: I will call Dr. Macqueen.

Dr. Macqueen was then examined by Mr. Baker.

*Q.* Can you tell me the date of this case?—*A.* The 1st of October, 1912.

*Q.* Do you remember going into the ward on that morning?—*A.* Well, the date of the alteration of the chart was October 1. It was several days after that when I noticed it.

*Q.* October 1 was the date when the temperature was entered on the chart?—*A.* Yes.

*Q.* And you noticed the erasure several days afterwards?—*A.* Yes.

*Q.* Did you inquire of the nurse? Do you remember which nurse it was?—*A.* I think it was Nurse Sharp.

Mr. Procter: It was the same nurse as before. I don't know why we are going into this.

The Commissioner: Yes, but I think you are responsible yourself for Mr. Baker going into it. I wanted him to prove the case. If you had not thought that that was necessary, it seemed to me it was sufficiently answered in the report of the special meeting of the Medical Board on October 16.

Mr. Procter: I quite agree if that is the case mentioned here in this charge. I don't see any object in going into it unless you do.

The Commissioner: I only want to report that the matter was investigated by the Medical Board, and Sister Victoria reported that it was not her custom to report high temperatures unless they were such as from the condition of the child she would expect, her custom being to have the temperatures taken again after some lapse of time, and to record them at lower temperature. If that is accepted, there is no use pursuing this any further.

Mr. Procter: I quite agree, Sir.

Mr. Baker: Of course, the point of the allegation is the alteration—the erasure, as I understand it—the charge there made is one that it was done with the express purpose of avoiding duty in the ward; that the reason it was done was this—I can ask this question of Dr. Macqueen if necessary—that the chart was altered in order that the nurse might say: "This is not a serious case; I need not stay here in the ward to look after it." I understand it was dealt with because the nurse was discharged.

The Commissioner: I think it was dealt with, and it appeared from the evidence of the sister herself that it was not her custom to record high temperatures, but practically she did record temperatures which were lower than the highest temperatures. Unless you wish to raise any point on it, I think we can pass on. Now we come to the statement that in many cases there are no records of pulses or respirations, even in the case of osteomyelitis. Will you, Dr. Macqueen, give us sufficient particulars of that case of osteomyelitis to identify it, if necessary?

Dr. Macqueen: I really forget the name of the patient.

The Commissioner: Which ward was it in?

Dr. Macqueen: In the Victoria Ward.

The Commissioner: (To Mr. Procter) Do you think it worth while to pursue that question?

Mr. Procter: Well, I don't know whether the same sister is implicated in this.

The Commissioner: It is in Victoria Ward. It was a case of osteomyelitis. Who was the surgeon concerned in this case?

Dr. Macqueen: I think it was Mr. Gostling. I could not be quite certain.

### THE HONORARY PHYSICIAN'S PART.

The Commissioner: What strikes me as a physician is that, if I was going round my ward, and found no pulse or respiration recorded for seven weeks, I should rather observe that fact myself, without finding it necessary to ask the house physician how it was.

Dr. Macqueen: I really forget the name just now.

Mr. Procter: Do you remember the date? Mr. Procter, continuing, mentioned a case of osteomyelitis and also the name of the hon. surgeon and of the patient, but witness said that was not the case.

Mr. Procter: Then what is the name?

Mr. Baker: Might he see the charts?

Mr. Procter: You are making serious charges against the management of the hospital. I think he ought to remember the name.

The Commissioner: I don't think you could expect any house surgeon or house physician to remember the name. He might remember the disease, but it would be very unusual to remember the case he had seen some time ago.

Mr. Procter: Can you remember the time?

Dr. Macqueen: It was in the beginning of October.

The Commissioner: This letter is dated October 14.

[Mr. Procter mentioned two other names, one of which Dr. Macqueen thought was the case referred to.]

Mr. Procter: Is it the same sister?

Dr. Macqueen: Yes.

Mr. Procter: I have nothing to say about it, Sir. I don't want to go into it any further.

Mr. Baker: It was said all along that it was the same ward and the same sister.

Mr. Neden: The penalty for the crime has been meted out; there is nothing further to say about it.

The Commissioner: Then there is nothing further to say about that. It practically comes to this: That it has been dealt with. The next statement is:

### "THE NIGHT STAFF INADEQUATE."

"That in our opinion the night staff of the whole hospital was inadequate for the treatment of the patients; besides there being no emergency night staff to take theatre or special duty if required. In support of this inadequacy two cases were quoted : In one ward there was a surgical case which required a great deal of attention. While the ward nurse was attending to this case, a patient in the other division of the ward got up, slipped on the floor, and broke his leg."

We might take that case first, then. Can you give us further particulars of that?

Mr. Baker (to Dr. Macqueen) : Do you remember when that was? I think the patient was not intended to get up.—The patient should not have got up at that hour of the morning.

*Q.* The patient got up whilst the nurse was away?—*A.* In the other division of the ward. The patient got up, slipped on the floor, and broke his leg.

The Commissioner : This is the paragraph, I think, in your letter that deals with it : "There is no emergency night staff who can undertake theatre or special duty when required. As a result of this, let me give one instance : In Ward 6 there was a bad case of chorea, and at the same time in Ward 7 there was a case of splenectomy. There was only one nurse to do 'special' between these two cases. As a result of this, the irregular nurse in Ward 7 had practically to act as 'special' to the splenectomy man, and while she was doing this a patient in the other division of the ward got up and slipped on the floor and broke his femur."

Dr. Macqueen : Yes.

### A PATIENT'S THIGH FRACTURED.

*Q.* (by the Commissioner). Will you tell us more particulars about that case? What was the man in for?—*A.* (Dr. Macqueen). Sinus of the knee.

*Q.* Was it that thigh that broke?—*A.* It was the same thigh.

*Q.* Had he been allowed up?—*A.* He had been up in a chair, and I think he had tried to walk with crutches.

*Q.* But that was not a patient whom you would suggest that it was necessary to have a "special" on, anyhow, was it?—*A.* No; but my point is that the nurse of the ward was acting as "special" at the time, and therefore that there was no nurse to look after the rest of the patients.

*Q.* Yes; but in the case of this particular unfortunate man who broke his leg, I understand from you that he was almost a sitting-up patient—that he had been allowed about on a chair?—*A.* Yes, but he should not have been getting up on his own accord, to attempt to walk without crutches.

*Q.* Yes; but his mental condition was quite good?—*A.* Yes, quite good. He was I forget how old—about sixteen or seventeen.

The Commissioner : Is the Surgeon in attendance?

Mr. Procter : Oh, yes, and the Nurse, too.

*Q.* (Mr. Procter) You were not present, I suppose, when he did get out of the bed?—*A.* No, I was not present. It was during the night or early morning.

*Q.* Is it a fact that he was convalescent?—*A.* He was.

*Q.* And due to go away from the hospital the next day after this happened?—*A.* He was going out in a few days.

*Q.* Is it a fact that he was going out next day?—*A.* I don't quite remember that.

*Q.* Do you know whether he had been walking about in the ward on the previous day?—*A.* Yes, with crutches.

*Q.* You say that he ought not to have got out of bed for any purpose?—*A.* He should not have got up by himself.

*Q.* I suppose he would know that, wouldn't he?—*A.* Well, I don't know.

*Q.* Do you know for what purpose he got out of bed?—*A.* No.

*Q.* I will call evidence. I suppose you don't suggest that there could be a special nurse for each patient in the ward?—*A.* No.

Mr. Watson : I understand the only point you make is that unfortunately the hospital was very busy, and the nurse was not at this particular patient's bedside, whether necessary or not.—*A.* Well, she was not in that division.

*Q.* I mean everybody was very busy?—*A.* Yes.

Mr. Baker : Do you know how many nurses there were on night duty?—*A.* One in each ward. The wards are divided into two divisions.

The Commissioner : Yes, and I am hoping to have an opportunity of seeing them.

Mr. Watson : He has answered it very frankly. He says the only suggestion he makes is that unfortunately the hospital was very busy at the time.

Mr. Procter : It was arranged that the special nurse for these two cases should have charge of both?—*A.* It was done without my permission.

Mr. Procter : I am instructed it was with your permission.

### TWO CASES DO NOT HOLD TOGETHER.

The Commissioner : Might we deal with this case first, and then go on to the other?

Mr. Procter : Really, they seem to me to hang together. The suggestion is that they were dividing the attention of the nurse.

The Commissioner : As a matter of fact, they do not hang together, because if it is established that this patient was a convalescent patient, in the same condition as you or I, and he got out of bed without leave and broke his thigh, it is very unfortunate; but we know nursing arrangements could not prevent that, and that is why I want to hear your Honorary as to the condition of the patient.

(It was explained that the Honorary in question—Mr. Hughes—was not in the hospital. Dr. Macdonald said Mr. Gayner and himself investigated the case, and their evidence could be given, but the Commissioner decided to hear Mr. Hughes at another time.)

The Commissioner : Now we can hear about this splenectomy.

Mr. Procter : Mr. Hughes is also in the splenectomy case.

The Commissioner : Yes, but the point is not the same. (To Dr. Macqueen) : Will you tell me about this case?

Dr. Macqueen : There was a special nurse who was looking after this case of splenectomy, a special day nurse and a special night nurse. This occurrence happened at night. The night nurse was sent to look after the case of chorea in Ward 6—the night special for the splenectomy case. I believe she was supposed to go down and see to the other case; but the two wards, I may say, were on different floors. She was supposed to go down and see the splenectomy case now and again.

The Commissioner : There was only one nurse to do "special" between these two cases, and they were on different floors.—*A.* Yes. *Q.* What was the age of the patient of chorea?—Dr. Shepherd : Seventeen. *Q.* Was it a severe case of chorea?—Dr. Shepherd : Chorea insaniens.

The Commissioner : We should all agree that chorea insaniens was a case which required very special attention because a patient might be out of bed or anywhere; and in a case of splenectomy—when was the operation done in splenectomy?

Mr. Procter : The 13th of November.

The Commissioner : But you see my point, Dr. Macqueen? Of course, a splenectomy that was going on perfectly well might have required very little special attention.

Dr. Macqueen : Yes, I quite see that, but the case did get septicæmia afterwards, and had to be re-opened.

The Commissioner : Was he in such a condition as to require the almost unremitting service of a "special"?

Dr. Macqueen : He was.

Mr. Hughes was in attendance and examined by the Commissioner : There is this case of a man who had synovitis, and having got out of bed broke his leg. He was under your care, and really the point we are concerned with is this: it is alleged that the incident shows some insufficiency in the number of the nursing staff, and I want you to tell us as to the condition of this patient, whether he was convalescent, whether he was sane and likely to know what he was doing, and generally whether the allegation, according to your opinion, is sustained on the evidence as regards this particular case.

Mr. Hughes: He is a man with synovitis of the knee whom I had allowed to get up on the couch with an idea of his going home in a day or two. He had come in with an old contusion of the knee joint, which had subsided, and I told Sister he might get on the couch and then go home in a day or two. Of course, it certainly meant he should be looked after, and I did not want him to walk about as an ordinary patient, because he had this weak knee.

The Commissioner: And his mental condition was quite sane?—Perfectly sane. And he took it into his head to get out of bed for some reason or other, and broke his thigh.

*Q.* It was not a case that you would have thought required a special nurse?—*A.* No. Of course, if he got out of that bed and was allowed to go to the lavatory he would have to have somebody in attendance to help him. I did not mean him to walk about the ward.

Mr. Baker: Didn't you order a "special" to look after this man?—Not this man.

Mr. Baker: Perhaps I am thinking of another case; I beg your pardon; then on this case I have no question.

The Commissioner: Now we come to the splenectomy case, a case of a serious operation, and for that case I think we have had in evidence that you ordered a "special"?

Mr. Hughes: Yes.

The Commissioner: And the difficulty seems to be that apparently at the last one "special" had to do for two serious cases, but so far as you were concerned you ordered a "special"?—Yes.

*Q.* Did you give permission for this "special" to be taken away?—*A.* No.

*Q.* Nor were you a party to any arrangement by which the "special" was shared with another case?—*A.* No.

Mr. Procter: Did it come to your knowledge that as a matter of fact the "special" was shared with the chorea case?—I don't think I heard that at the time.

*Q.* Have you heard it since?—*A.* Yes.

*Q.* Did you seen anything improper in it. Would you have had any objection to it at the time?

The Commissioner: I don't think that really is a proper question to put to Mr. Hughes. That perhaps is a thing on which I have to form an opinion. Mr. Hughes tells us that in his view the splenectomy case required a "special," and with that I think anybody who knows the severity of an operation for splenectomy, and the complications that would probably follow it, would coincide.

### MR. GAYNER AGAIN.

Mr. Procter: I should like to call Mr. Gayner, who had charge of the chorea case.

Mr. Gayner: The responsibility of the chorea case did rest with me to a considerable extent. It was my case. I was asked, on the morning before the night on which the child fell out of bed, whether I would order a "special" for the case, and I said "No," that I thought the patient would be kept in bed by means of tying a sheet, and that it would be better not to order a "special." I gather that the patient became much worse, and arrangements were made by the hospital physician to procure a nurse: if I had ordered a "special" in the morning of course it would have been better, but it is easier to get "specials" if you have an hour's notice.

The Commissioner: Is that your view? Do you remember that incident, Dr. Macqueen?

Dr. Macqueen: The chorea case was under the care of Dr. Shepherd.

Dr. Shepherd: What is the exact question?

The Commissioner: The exact point is this: Mr. Gayner saw the patient in the day, and did not regard the patient's condition as so serious as to require a "special," and he thought the patient might be kept in bed by tying a sheet. Well, then, the patient is said to have got very much worse in the evening, and arrangements by which one "special" was provided for the chorea insanens and the splenectomy are supposed, Mr. Gayner suggests, to have been made by you.

Dr. Shepherd: Well, I made arrangements that my case was to have a "special" as it required it. This would be between 10 and 12 at night. It was generally worse from 12 up to 2 or 3 in the morning.

The Commissioner: Where are these special nurses provided from?

Dr. Shepherd: Well, they could be provided, I suppose, from the ordinary hospital staff.

The Commissioner: Then your interests were confined to secure a "special," if you could, for the chorea?

Dr. Shepherd: Yes. The chorea was so very ill that I expected the child would die.

The Commissioner: Was there any request for a "special" for each case?

Dr. Shepherd: Well, I requested a "special" for that case.

The Commissioner (to Dr. Macqueen): Then your case was the splenectomy?—My case had a "special," and I did not give permission for the "special" to be taken off.

The Commissioner (to Dr. Shepherd): Then you did not get your "special," you got half of one?—Yes, evidently the "special" was halved.

The Commissioner (to Mr. Gayner): Is that so?—I never clearly understood. There was a confusion of evidence, and I don't know quite what arrangements were made as to the "special." Of course, the orders were verbal; they are not in writing, and I am not quite sure what happened.

The Commissioner: Whom would you ask for a "special"?

Dr. Shepherd: The Matron.

The Commissioner: Then perhaps she can tell us whether she had a request?

Mr. Gayner: I expect so.

Mr. Procter (to Dr. Shepherd): Perhaps you can tell us, Dr. Shepherd, when did the chorea case actually fall out of bed.—I think that night: I cannot be exactly certain on the point, but I think the girl fell twice out of bed, because she remembered it, and mentioned it afterwards.

*Q.* According to my information, it was in the daytime. —*A.* It may have been once in the day.

*Q.* Do you say she fell out in the night time?—*A.* Well, I myself have seen her body three-quarters out of bed.

The Commissioner (reading from letter): It is said here that "The chorea case in 6, before the 'special' was obtained for her, twice fell out of bed. This fact she remembers and made mention of, and will in all probability do so again when she returns home."

Mr. Procter: I understand these occasions were in the daytime, when the charge here is in regard to the inadequacy of the night staff.

Dr. Macqueen: The chorea case fell out of bed in the night.

### THE MATRON AGAIN.

The Matron was then called.

The Commissioner (to the Matron): I have to ask you about two things; one was a case of chorea insaniens, severe chorea, and the other was a splenectomy. It appears that there was a "special" on the splenectomy case, and that a "special" was requested for a chorea case. It was not requested by Mr. Gayner, who has told us that he did not think it necessary when he saw the patient, but later in the evening the case became very much worse. Did you get, as a matter of fact, any request for a "special" from Dr. Shepherd?

The Matron: No, I have no recollection of that.

The Commissioner: The evidence is that there was a "special" divided between the two.

The Matron: Yes, I think I had permission for that to be done. I think we had Mr. Macqueen's permission for the "special" to be shared. I have no order for a "special" for the chorea that night. The child got much worse.

The Commissioner (to Dr. Shepherd): Did you ask for a "special" for the chorea?—I did.

The Commissioner (to the Matron): Did that reach you?

The Matron: I have no request from Dr. Shepherd for a "special."

Dr. Shepherd: It is possible I might have asked the night sister to ask for a "special"; I cannot recall the exact incident at the moment, but I know I asked for a "special" for a case that was required most urgently.

Mr. Baker (to the Matron): Did a request come from Dr. Shepherd, directly or indirectly?

The Matron: The only thing I recollect is an arrangement being made that the "special" should work between the two cases.

Mr. Baker: At what time was that?

The Matron: I really cannot remember.

Mr. Procter: What you remember is an arrangement for a "special" to be shared.

The Matron: Between the two cases. I should not remove a "special" without permission.

Mr. Procter: By whom was that arrangement made or agreed to?

The Matron: I think between the night sister and the residents.

Mr. Baker: Both Dr. Shepherd and Dr. Macqueen?

The Matron: It would have to be, because one was a medical case, and the other surgical.

Mr. Baker: You think they made the arrangement between them with the night sister?

The Matron: I know it.

The Commissioner: As a matter of fact, this particular question did not come before you, but before the night sister. Could the night sister have got two "specials"?

The Matron: Well, of course, late at night—it was about midnight that the child was much worse.

The Commissioner: Would it have been practicable for her to get a "special"?

The Matron: If they are ordered they are almost bound to get them.

The Commissioner: Even at midnight?

The Matron: They could make an arrangement.

The Commissioner: Where would you have got them from? Your regular staff?

The Matron: I must have done at that time of night.

Mr. Baker: You had one "special"; it was a question of getting a second?

The Matron: Yes.

The Commissioner: But the night sister would have had to take her from your own staff?

The Matron: Yes.

The Commissioner: Then, perhaps we might see the night sister.

### THE NIGHT SISTER WAS THEN CALLED.

The Commissioner: The point I have to ask you about is with regard to "specials" on a certain night. There was a case of severe chorea, and a splenectomy. I understand that the splenectomy had got a "special"?—Yes.

The Commissioner: Dr. Shepherd says that he asked for a "special" for his chorea. Did any request for a "special" for the chorea come to you? And then, further, to make quite clear what we are at, there are suggestions further made that perhaps there was some arrangement by which the "special" should be shared between the two cases.—Yes; I think the house physician and the house surgeon said that could be done.

The Commissioner: Your recollection is that under the circumstances, knowing how difficult it was to get a "special" at twelve o'clock at night, or some time like that, they consented to do with one between the two?—Yes.

Mr. Baker: Is your recollection of that quite clear about this incident?—I think so. I am perfectly certain.

Mr. Baker: And you say that no request came to you from Dr. Shepherd for a "special"?—No; I can't remember any.

Mr. Baker: And you say that later on both Dr. Shepherd and Dr. Macqueen consented to having one between the two cases?—It was mutual. They were both in the ward at the same time.

Mr. Baker: And they agreed to that?—Yes.

Mr. Baker: I think it only fair to tell you that they both deny that. They say that they never consented to do with one nurse between the two. Are you quite sure?—I feel perfectly certain about what Dr. Shepherd and Dr. Macqueen said, and they said it quite nicely too.

Mr. Baker: Oh I am sure they would say it nicely. I am not suggesting they would quarrel; but was it a case of doing the best they could? I want you to be careful about this, because they say they were not at all satisfied with having one nurse between the two cases. Of course, if it was impossible to get another nurse they would have had to do with one?—It did not appear so; they appeared quite satisfied that that could be done.

The Commissioner: I think Dr. Macqueen and Dr. Shepherd should have an opportunity of saying point blank that they did not make this arrangement.

Dr. Shepherd: I remember asking Dr. Macqueen if such a thing would be possible, and he emphatically said "No, that his case could not do with that."

Mr. Baker: Dr. Macqueen, do you remember that being said to you?

Dr. Macqueen: I remember giving permission for the "special."

Mr. Baker: No; but you have heard Dr. Shepherd's question. Did you say to him you could not get on without your "special"?

Dr. Macqueen: Yes; I said the case was so bad that "special" could not be taken off.

Mr. Baker: Was that in the presence of the night sister?

Dr. Macqueen: I don't know.

The Commissioner: Have you any recollection of being in the same ward with your colleague and the night sister, and discussing the matter together, because the sister tells us the arrangement was made in an amicable and friendly manner between the three?

Dr. Macqueen: I remember seeing the case of chorea, along with my colleague, but no arrangement like that was discussed.

The Commissioner: We cannot carry that any further. Then we come to the next paragraph:—

"As nothing further was done, we, on December 27, 1912, sent a report to the House Committee, of which the following is an extract:—

### "AN ABSENCE OF CONTROL."

"Ever since the time of our separate appointments we have been more than dissatisfied with the general administration and supervision of matters in this house. Some months ago it was reported to the Matron that the condition of affairs in Victoria (Children's) Ward was extremely unsatisfactory, that the sister of the ward was . . . failing in her duties as a nurse; but the Matron, instead of taking such report from a Resident, as a Matron should, wished to argue the matter, and deliberately shut her eyes to the delinquencies involved. The sister was afterwards caught red-handed, and, on this being reported to the Matron, the latter was exceedingly insolent." In the original letter that appears to be "exceptionally insolent."

"(For further details of this we refer you to the Medical Board before whom the case came.) This is a concrete instance, but there are many other derelictions of duty which exist, and are not so easily brought home."

Do you wish to say anything on that? As it stands, it is hardly a matter one can investigate.

Mr. Baker: It was Dr. Macqueen's case. I might just ask him one question.

The Commissioner: I mean we know all about the sister. We know she has gone. It only concerns the behaviour of the Matron.

Mr. Baker: I don't think it was in connection with the sister who has gone.

The Commissioner: It says so here. Shall I read you the report?

"We, the undersigned, being the resident medical officers of the above institution, wish to make a report on the condition of affairs existent in this hospital."

This letter is undated, but it appears to have been on December 27, 1912.

"As was pointed out at the last meeting of your committee if we wished to complain we should do so formally in writing to the House Committee, we do now in pursuance thereof make formal report and complaint.

"Ever since the time of our separate appointments we have been more than dissatisfied with the general administration and supervision of matters in this house. Some months ago it was reported to the Matron that the condition of affairs in Victoria Ward was extremely unsatisfactory, that the sister of the ward was deliberately failing in her duties as a nurse; but the Matron, instead of taking such report from a resident, as a matron should, wished to argue the matter, and deliberately shut her eyes to the delinquencies involved. The sister was afterwards caught red-handed, and on this being reported to the Matron the latter was exceptionally insolent (for fuller details of this we would refer you to the Medical Board before whom the case came). This is a concrete instance, but there are many other derelictions of duty which exist, and are not so easily brought home."

Mr. Baker: The point would rather seem to be not only a complaint against the sister who has gone, but a complaint against the Matron for not reporting.

The Commissioner: The complaint is clearly against the Matron, but it is not clear what the complaint against the Matron is, except that when the Resident said something to her and reported a sister as failing in her duties, she did not take the report as a Matron should.

Mr. Baker: But I think there is a specific complaint of not forwarding charges.

The Commissioner: I don't see that.

Mr. Baker: I think it dealt with various incidents. I agree it is a general allegation.

The Commissioner: I cannot see what we are to charge the Matron with except not receiving the Resident's report as a Matron should.

Dr. Macqueen: I may explain that prior to the complaints to the Matron on October 13 I had reported, or asked the Matron, about charting in the Victoria Ward, and she started to argue the matter.

(At this point the Matron was sent for again.)

The Commissioner remarked to Dr. Macqueen: Your language is quite strong. (Reading:)

"While dealing with the staff we may add that the general question of the nursing staff requires serious consideration, and if the general public became aware of the callous ineptitude of the head of the nursing staff, it would demand the resignation, not only of the defaulting individuals, but also of the House Committee, who must be held responsible."

### DR. MACQUEEN AND THE MATRON.

The statements are very strong, and I think Dr. Macqueen should desire to give some evidence. (To the Matron): Matron, I have got you here to listen to Dr. Macqueen's allegations as regards your own conduct. It is the first time I have heard of it, and I think you ought to hear it.

Dr. Macqueen: Dealing with the first matter, prior to the statements in this letter of the 14th, I had complained to Matron about mis-charting the Children's Ward, and about the children being tied down in bed, and Matron argued the matter.

The Commissioner: Give us a little more detail as to arguing the matter. Do you mean she said the charts were correct, or what line did the argument take?

Dr. Macqueen: She said that the sister of the ward was a thoroughly capable woman who had been well recommended to her, and that she did not see how such things could be.

The Commissioner: That is as regards charting?

Dr. Macqueen: Yes.

The Commissioner: We will take the tying of children in bed. (To the Matron): Is that a correct account?

The Matron: Yes.

The Commissioner: And of course subsequently you found out that this sister, at least by her own confession, had been charting improperly?

The Matron: I did not know she confessed to improper charting. She confessed to striking the child.

The Commissioner: I am quoting from the statement of the Medical Board:

"Sister Victoria reported that on December 6 she struck the child, and that it was not her custom to record high temperatures unless they were such as from the condition of the child she would expect, her custom being to have the temperatures taken again after some lapse of time, and to record any lower temperatures."

You could hardly call that correct charting, could you?

The Matron: I see.

Dr. Macqueen: I made this complaint to Matron, and I never heard any more about it.

The Commissioner: What did you expect to hear about it?

Dr. Macqueen: That something had been done concerning the matter.

The Commissioner: How long were these incorrect chartings complained of before the whole question came out in October?

Dr. Macqueen: I think this was some time in August.

The Commissioner: Do you mean that instead of inquiring into the fact whether these charts were incorrect the Matron met your complaint by saying that she was a very capable, well-recommended sister, and she had no reason to suppose she was incompetent, or doing anything of that sort? Do I summarise your view properly?

Dr. Macqueen: Yes. That is the answer I got from Matron. I never heard afterwards whether she had done anything in the matter.

The Commissioner (to the Matron): What have you to say to that?

The Matron: It is right, Sir. It was in September, sometime, that it was mentioned to me.

The Commissioner: And you were satisfied that the sister was competent and you pursued the question no further?

The Matron: I did report the matter.

The Commissioner: Whom did you report it to?

The Matron: To Dr. Evelyn.

The Commissioner: But the Resident did not know really whether anything or nothing had happened.

The Matron: I told him I would look into it.

Mr. Watson: This letter is a very serious charge of corrupt administration.

Mr. Procter: Before we leave this, might I have this: Was this purely a general charge, or did he bring specific instances of mis-charting to the notice of the Matron?

Dr. Macqueen: This complaint in this letter was purely general.

The Commissioner: As regards this ward?

Mr. Procter: I am speaking of the mis-charting.

Dr. Macqueen: I did not mention any particular instance.

Mr. Procter: You did not show her any charts or mention any particular instances?

Dr. Macqueen: No.

The Commissioner: I suppose you said you had an impression that the charts were wrong? Of course, it would not have shown that the charts were wrong if you had shown her any particular chart?

Mr. Procter: What do you actually mean by mis-charting?

Dr. Macqueen: That there was temperature taken and it had not been put on the chart.

Mr. Procter: Do you mean correctly or not at all?

Dr. Macqueen: That it had been put down incorrectly.

Mr. Procter : Had you specific instances in your mind? Had they come to your knowledge?

Dr. Macqueen : Yes.

Mr. Procter : Had you a record of them?

Dr. Macqueen : They had been reported to me.

Mr. Procter : Who had reported them?

Dr. Macqueen : The staff nurse.

Mr. Procter : Not the sister?

Dr. Macqueen : No.

Mr. Procter : Did you make any note of these specific instances?

Dr. Macqueen : No.

Mr. Baker : In one case he did.

Mr. Procter : In one case, but prior. (To Witness) Did you regard it as a serious matter, this charting or omission to report?

The Commissioner : Oh, I think one can say that at once. It is a very serious matter. I don't think any doctor would deny that.

Mr. Procter : Why did you not make notes of the cases that came to your knowledge?

Dr. Macqueen : Well, they were reported to me after they were mis-charted. It was a sort of general intimation to me that mis-charting had been taking place in the ward.

Mr. Procter : Surely it would be possible to get specific instances? Would it be your duty, or whose duty would it be?

Dr. Macqueen : Well, the Matron is in charge of the Sisters.

Mr. Procter : You had the general control, surely, of the ward, hadn't you?

The Commissioner : Yes, but the routine would be that if Dr. Macqueen had any complaint against the Sister or a nurse he would make that complaint to the Matron, so that that would be quite in order for him to make a complaint in that fashion. I suppose what happened was that you heard from some source, perhaps the nurse, that these charts were not correct. You mentioned the matter to the Matron, and you would have liked her to take it up at once and investigate it, and the complaint against her is that she did not investigate it as far as you know, or at any rate that you don't know what she did; but she might out of courtesy have told you what the result of her investigation was?

Dr. Macqueen : Yes.

Mr. Procter : Then can we get anything like a time when he first mentioned this to the Matron? The Matron says September was the first time.

Dr. Macqueen : It might have been. It was some time previous to the letter.

### SEVERAL CHILDREN TIED DOWN IN BED.

The Commissioner : Now, you have made another statement there, Dr. Macqueen, just now, about having children tied down in bed. It is mentioned later on, but perhaps it is as well to take it here. Will you let us know something about that? The paragraph is 17 :

"When we first went to the hospital we found it was customary to tie the children down in bed, no matter what the case might be. The methods of tying was by bandage round the shoulders, wrists and ankles."

Can you give us some particulars?

Dr. Macqueen : This refers to the same Sister.

The Commissioner : But you have told us that, having found this out, you reported it to the Matron, and that, I understand, is one of your objections to the way in which the Matron behaved towards you.

Dr. Macqueen : I did tell her about it.

The Commissioner : What did she say?

Dr. Macqueen : She did not say anything.

The Commissioner : When was this habit of tying the children down given up? You say that it was customary to tie the children down in bed.

Dr. Macqueen : My first day in the hospital, I remember going round the Children's Ward, and I found four or six cases on that occasion, and the ward was only half full.

The Commissioner : Did you know of this habit of tying the children down, Matron?

The Matron : Yes; some of the children were tied down. The matter of tying children down was not one of the complaints made to me.

Mr. Watson : I don't think you say in the letter there about tying children down.

The Commissioner : No, that is why I am taking it now. There is no evidence except the evidence here.

Mr. Baker : On this case, if you like, Dr. Shepherd will speak too.

The Commissioner : When you came to the hospital you found that the children were tied down; at any rate, six of your cases were tied in bed?

Dr. Macqueen : Yes, the first morning.

The Commissioner : But did you call attention to it?

Dr. Macqueen : I asked the Sister to cut the strings. In several cases afterwards I found the children tied down.

The Commissioner : I don't see how the Matron comes into the charges at all, because she had nothing to do with it. He told the sister to cut these things.

Dr. Macqueen : Well, I should be reporting this as an occurrence in the ward.

Mr. Baker : Did you make a general complaint about the tying of the children to the Sisters?

The Commissioner : To the Sister Victoria.

Mr. Baker : Expecting it to be called to the attention of the Matron? Did you never complain about the children being tied down?

Dr. Macqueen : I did.

Mr. Baker : And you told Sir Cooper that you ordered the strings to be cut. Did you not also make a general complaint that it should not be done?

Dr. Macqueen : I made a complaint, yes, to the Matron.

Mr. Baker : Oh, you did make a complaint to the Matron, and after that were they tied any more?

Dr. Macqueen : On one or two occasions I found they were.

The Commissioner : I cannot quite see what your complaint against the Matron is as regards this particular matter.

Mr. Baker : I will ask Dr. Shepherd about this.

Dr. Shepherd : I found two children tied down in the period in which I was resident in the hospital. On that occasion I simply cut the tapes and made the remark to the Sister that it was never to occur again.

Mr Baker : That was all you said. You made no more definite complaint than that?

Dr. Shepherd : Yes.

Mr. Baker : That was the first occasion; was there any other occasion?

Dr. Shepherd : There was no other occasion. That was made long after Dr. Macqueen had ever complained.

Mr. Baker : You, I think, first came in September?

Dr. Shepherd : Yes.

Mr. Baker : And, do you remember, was it early in your visit that you found children tied?

Dr. Shepherd : Yes, I should say it would be within the first month.

Mr. Baker : Then I ought to have asked Dr. Macqueen the first occasion he found the children tied.

The Commissioner : Dr. Macqueen says when he first came into residence.

Dr. Macqueen : That was in May.

Mr. Baker : Did you make any definite complaint to the Matron about children being tied?

Dr. Shepherd : No.

Mr. Baker : Or did you simply call attention to it?

Dr. Shepherd : I simply called the attention of the sister to it.

The Commissioner : You seem to have adopted quite a good way of calling her attention to it by cutting the bandages.

Dr. Shepherd : I thought it would be the most effective.

The Commissioner : But it is not denied, Matron, that the children were tied down?

The Matron : Some of them.

The Commissioner : To your knowledge that was so?

The Matron : Yes.

Mr. Procter : I want a general question. Is this a surgical or a medical matter?

The Commissioner : It may be medical or surgical.

Mr. Procter : Dr. Shepherd, are you saying that under no circumstances is it proper to tie a child down in bed?

Dr. Shepherd : I maintain it was not. It is contrary to all asylum rules, and therefore I think it holds in the case of sane people as well.

Mr. Procter : Why?

Dr. Shepherd : Why should a child be tied down?

Mr. Procter : Don't ask me; I am not a medical man.

Mr. Baker (to Dr. Shepherd) : You are entitled to give your opinion, as you have been asked why.

Mr. Procter : You say it is your considered opinion that it is improper in any circumstances to tie a child down in bed?

Dr. Shepherd : Yes, after the fashion of the two cases I saw tied down.

Mr. Procter : Do you mean to say it might be justified in a certain way and in certain circumstances?

Dr. Shepherd : If a child was one of those very excitable children that liked to play about its cot a lot, and was very well, I believe that sometimes in certain hospitals there is a wooden rail round the child's body, and from that there is a band which is attached to the crib or cot, so that the child cannot get too much movement or hang out.

### IMPROPER METHODS OF TYING CHILDREN.

Mr. Procter : Is it your complaint that the method adopted of tying down these children was improper?

Dr. Shepherd : Yes.

The Commissioner : The method of tying was by bandage round the shoulders, wrists, and ankles?

Dr. Shepherd : Yes.

Mr. Procter : In every case?

Dr. Shepherd : In the two cases which I saw.

Mr. Procter : I am reading from The Hospital. "When we first went to the hospital we found that it was customary to tie the children down in bed no matter what the case might be." Is that a correct statement?

Dr. Shepherd : There was no reference to the two cases which I saw. I did not see any necessity for their being tied.

Mr. Procter : This suggestion——

Dr. Shepherd : If it is Dr. Macqueen referring to what occurred when he first came, I was not there at that time I came subsequently.

Mr. Procter : And you only speak of two cases?

Dr. Shepherd : I only speak of two cases which occurred in my time.

Mr. Procter : Who was the Honorary in charge of those two cases?

Dr. Shepherd : I cannot answer that question.

Mr. Procter : You don't remember?

Dr. Shepherd : No.

Mr. Procter : I suppose he would know?

Dr. Shepherd : He would know if he had been in the ward.

Mr. Procter : It would have been done, of course, with his knowledge?

The Commissioner : You would agree that it would have been done with his knowledge?

Dr. Shepherd : I cannot speak for any other body.

Mr. Procter : That would be the ordinary course of procedure?

### TIED DOWN THE WHOLE DAY.

The Commissioner : Were they tied down the whole of the day so far as you know?

Dr. Shepherd : So far as I know it was the intention for them to be left tied down.

The Commissioner : So that if he had gone round——

Dr. Shepherd : I expect he would have seen them.

Mr. Procter : Will you tell us what these cases were?

Dr. Shepherd : I can't remember them. Except I do remember one now. One was that child James Merrick.

The Commissioner : The empyema child?

Dr. Shepherd : The other I cannot remember.

Mr. Procter : What was the case?

Dr. Shepherd : Empyema.

The Commissioner : That is the case we have already investigated. Was he tied down before being operated upon?

Dr. Shepherd : I don't know. He was operated on before I came.

Mr. Procter (to Dr. Macqueen) : Do you agree generally with Dr. Shepherd's views upon tying down children?—I do.

Mr. Procter : Would you object——

Mr. Baker : If you are going to call evidence on it, hadn't you better put specific instances prior to the time when Dr. Shepherd was there?

Mr. Procter : He is giving his general opinion that it is improper in any circumstances.

Dr. Macqueen : Tying them down in the manner adopted there.

Mr. Procter (to Dr. Macqueen) : Then you don't express the opinion that in any circumstances and any manner it is improper?—No.

Mr. Procter : But you object to the way in which it was done?—Yes.

Mr. Procter : I think you said more than half your patients were tied down on the first day you came?—*A.* There were over six.

*Q.* Do you remember what sort of cases they were?—*A.* I don't quite remember the cases, but they were cases in which there was no necessity to have the children tied down.

*Q.* Not at all?—*A.* Not at all.

*Q.* I put this to you—in a case of operation for hernia do you consider that a proper case to tie down?—*A.* Not in the manner in which they were tied here.

*Q.* In a different manner?—*A.* Yes.

*Q.* I suppose your cases would be under the care of some honorary officer?—*A.* Yes.

*Q.* And he would know of the tying down?—*A.* Not unless he examined the child. If the child was covered up with the bedclothes, how could he see it?

Mr. Baker : I understand that when you found the children in that state you cut the strings yourself?—I did.

*Q.* And also the bedclothes might prevent the house physician seeing that the child was tied?—*A.* Yes.

*Q.* Do you, in fact, know whether it was ever brought to the knowledge of the house physician that these children were tied?—*A.* I was house physician when I came here, and I found them tied.

Mr. Procter : Did you report to the honorary officer your opinion about tying down?—No; it seemed to be the custom here, so I did not report.

*Q.* You did not report that you had cut the bandages?—*A.* No.

Mr. Baker : You cut the bandages straight away, did you?—Yes.

Mr. Procter : And he did not report what he had done?—No.

Mr. Watson : Do I understand that neither you nor Dr. Shepherd reported to anybody that you had discovered these children tied down?—Yes, I discussed it with some members of the staff.

*Q.* Do you mean to say you did not report it to anybody at all?—*A.* Well, I was informed by the Resident here when I came——

*Q.* But would you answer the question? Did you report that fact on your first day? Did you ever report that breach of duty, or whatever it was, to anybody?

Mr. Baker : Because he said he was informed that it was the practice.

### CONTINUANCE OF TYING DOWN CHILDREN.

Mr. Watson : I don't want to know that. I want to know whether he reported it.

Dr. Macqueen : No, I did not report it, except afterwards, when I made a general complaint to the Matron.

*Q.* Well, how long after was that?—*A.* Perhaps two months.

*Q.* Therefore the practice was still going on?—*A.* Yes.

*Q.* But you would not allow what you considered a distinctly wrong practice to continue because the outgoing surgeon told you it was the practice?—*A.* No; I started by cutting the bands.

Mr. Watson : Well, I asked you whether you reported it.

Mr. Procter : There is a general question I want to apply to a number of these charges, so perhaps it will be convenient if I put it now?

The Commissioner : Well, won't you finish this one?

Mr. Procter : I want to give evidence on this, but I want to ask Dr. Macqueen or Dr. Shepherd generally about a number of the charges.

The Commissioner : Well, you know what your question is going to be.

Mr. Procter (to Dr. Macqueen) : You remember the date before you left the hospital you saw Dr. Macdonald and Mr. Gayner?—Yes.

Mr. Baker : When was this? Before his final departure?

Mr. Procter : Yes. Were you asked if you had reported all the complaints you had to make against the hospital?—I don't remember being asked that question.

*Q.* Well, will you say that you were not asked the question?—*A.* No, I won't say so.

*Q.* You had not, as a matter of fact, made this among other complaints at that time?

Mr. Baker : What, about tying?—No, I don't think I had.

Mr. Procter : The first time this complaint is made it appears in the columns of The Hospital?—No, I had made a mention of it.

*Q.* You have no record of it? You told us you did not report it to the honorary staff?—*A.* I have discussed it with members of the honorary staff.

*Q.* Did you mention it to Dr. Macdonald and Mr. Gayner that day when they asked you if you had anything else to complain of?—*A.* No, there were so many complaints.

### THE MATRON EXAMINED.

(The Matron was then examined on this matter.)

The Commissioner (to the Matron) : Did Dr. Macqueen mention to you about tying the children?—He mentioned mis-charting, that the children were dirty, and that the sister could not give information about cases.

Mr. Baker : Do you never remember his having spoken to you about the children being tied down?—No.

*Q.* Neither Dr. Macqueen nor Dr. Shepherd?—*A.* Dr. Shepherd never did, I am quite sure; and I think Dr. Macqueen's complaints were confined to mis-charting, that the children were dirty, and that the sister could not give information about the cases.

(Dr. Shepherd was then examined on the same question.)

Mr. Procter (to Dr. Shepherd) : Do you remember seeing Dr. Macdonald before you left?—Yes.

*Q.* Was the question put to you?—*A.* I cannot recollect at the present moment, there were so many questions put to me.

*Q.* As to whether you had any further complaints to make? Was that question put to you?—*A.* I don't know.

*Q.* Will you say it was not?—*A.* I will not say it was not, and I cannot say it was.

*Q.* As a matter of fact had you, with regard to this tying down, made any complaints before to anybody?—*A.* Well, I did not think the thing was of such a nature that I would refer it to anybody. I thought that if people knew they would not allow such a thing done themselves, and therefore I took what I considered the best method, and simply cut the bands.

*Q.* You never spoke to the Honorary about it?—*A.* No.

*Q.* You had three months' experience which you were putting against the experience of the honorary officers of this Institution?—*A.* No; I am putting experience of other hospitals, and what I know about them. I know that such a thing should not be done.

Mr. Baker : Do you know what was the practice of the previous Residents? Do you know if a set of rules had been drawn up by them as to tying children?—I believe I have heard something mentioned during the residency of Dr. ————.

Mr. Baker (to the Matron) : Were there a set of rules drawn up, Matron?—Not that I am aware of.

*Q.* You don't remember any?—*A.* I am not aware of any rules of that sort.

The Commissioner : It could not have been the duty of the Residents to draw up rules. If such rules were drawn up it must have been done by the Medical Board.

Mr. Procter : We haven't any, Sir.

Mr. Neden : I have not seen any rules with regard to tying up. It must be a regulation of their own.

Mr. Baker : I was suggesting it as a regulation of their own.

### DR. EVELYN'S REPORT ON CHILDREN TIED DOWN.

Dr. Evelyn was then called by Mr. Procter.

Mr. Procter : You have heard what has been said, Dr. Evelyn? I think I had better leave you to make your own observations.

Dr. Evelyn : The custom has been in vogue in this hospital of what is termed tying down children ever since I have been connected with the hospital. It sounds possibly to the lay mind rather a severe measure to take in connection with the control of children, but I have found in my experience in the hospital here that it is necessary at times to restrain the movements of the children, and the method in vogue here is by means of a simple band with two armlets attached. (Dr. Evelyn produced one of the arrangements in question, and proceeded to describe its use.) This band is put over the little child, and its arms are brought down here, and then this band is brought behind the child's back and brought through this armlet on one side, and through the armlet on the other, so that you see the child's body is in between these little bands, and these bands are tied on either side of the cot, so that the child is kept down partly in its cot. It can use its hands freely, but it cannot get up to cause itself any harm. I have personally countenanced this condition of things in cases of acute eczema, a condition that any gentleman present here having suffered from knows is a most distressing thing, and one which causes you to scratch, scratch, scratch. It is only natural that a little child will not understand it must not scratch, and the inability to provide nurses to look after every child that has eczema or has recently been operated upon necessitates the use of some restraint. I should very much like to know how, with a staff of one sister and three nurses in a ward of 22 children you are to restrain children who have been operated upon from doing themselves harm, or children who are suffering from irritable diseases doing themselves harm also. I know that the tying down of children may appear to the lay mind to be a very serious thing indeed, but I think after what I have shown you there is nothing very serious in the plan. I have considered the evidence of the two doctors —Macqueen and Shepherd—and I must say this, that I don't ever remember either of them having drawn my attention to the fact that any of the children under my care were being tied down. If they had done so, I should most certainly have said what I thought on the matter, and if I thought the child ought not to have been tied down I should have given orders for it to have been released. If I thought it ought to remain tied down, I should have said so.

The Commissioner : The point I was just going to put to you is this. The evidence of Dr. Macqueen and Dr. Shepherd is that these children were tied down in an improper manner. What they say here is that the method of tying is by bandage round the shoulders, wrists, and ankles. That, of course, is not the method you are demonstrating to us at all, and that is the reason I have handed those gentlemen these bandages to know whether that is really the thing that is in use.

Dr. Evelyn: That is the thing which is in use, and there are many others of them in the drawer in Victoria Ward. I think I am right in saying that there is one child now on the surgical side that is in this way restrained in its actions. I dare say in the case, for instance of a child with general eczema, of which I have seen two or three recently, there are bandages applied to the feet to keep the child from kicking his feet about. First a layer of cotton-wool is put over the child's ankles, and then a bandage with a slip knot is put over the ankle, and that is tied lightly to the side of the cot, the child thereby being restrained in its actions, but not tied so that it cannot move hand or foot. It is not tied so that it cannot move hand or foot.

The Commissioner: That would be a method that you would regard as applicable to eczema, but not necessarily for any less irritable disease?

Dr. Evelyn: No.

Mr. Procter (to Dr. Evelyn): Is it correct to say that it was customary to tie the children down in bed, no matter what the case might be?—Oh, no, perfectly incorrect.

Mr. Watson: What age is referred to?—Children in Victoria Ward are taken up to seven.

*Q.* And up to what age would they be tied?—*A.* The older the child the less need there is for restraining it, because you can bring reason to bear on a child beyond six or seven.

Mr. Procter: I should just like to ask you, Dr. Evelyn, how long have you been in practice?—Thirty-six years.

*Q.* And how long have you been an officer of this institution?—*A.* Since 1900. It will be thirteen years in March.

*Q.* With all your experience, what is your opinion generally about tying down?—*A.* I am convinced that it is the only thing you can do if you cannot provide a sufficient staff of nurses to look after each child that requires attention.

*Q.* Has there been any tying down in a manner that you consider improper?—*A.* No, never.

### ALL THE CHILDREN TIED DOWN.

Mr. Baker: I understand all the children who come into this ward are tied down in the way you first of all described?—Yes.

*Q.* All of them?—*A.* As far as I know, all of them are.

Mr. Baker: I wanted to know.—All that we think are necessary to be tied down. I had already answered that question.

*Q.* Well, I had not followed it. You need not say a thing because I would like it. You say it is only in cases of eczema, or severe eczema, that they are tied in any other way?—*A.* Yes.

*Q.* How many children are there on an average in this ward?—*A.* The ward is supposed to be over full if there are over twenty-two.

*Q.* Then there was one sister and three nurses for twenty-two children?—*A.* One sister and four nurses. [Dr. Evelyn had previously said there were three nurses, but he never corrected this.]

*Q.* Do you agree with me that it is because—no doubt for very good reasons—the hospital cannot supply more nurses than that, that it is found necessary to tie the children?—*A.* Certainly.

### INSUFFICIENT NURSES.

*Q.* You agree with that?—*A.* They are tied down there because we cannot provide the ward with sufficient nurses.

*Q.* In the cases of eczema, you say that the children generally have their hands and their ankles tied?—*A.* If it is general eczema, yes, but not if it is only in connection with the upper part of the frame.

*Q.* Do you know whether cases of severe eczema have been treated by your colleagues, I think, in which there was no tying at all?—*A.* I am not aware of the fact.

Mr. Baker: I do not know whether Mr. Gayner is going to be called on this. My instructions are—perhaps Dr. Shepherd would get up now?

The Commissioner: Have you finished with Dr. Evelyn?

Mr. Baker: Yes. I wanted to ask Dr. Shepherd just the one question about tying ankles. We had not dealt with that before.

Mr. Baker (to Dr. Shepherd): Have you had in the wards to deal with a case of a child with severe eczema?—Yes.

*Q.* Have you dealt with that child without tying its hands or ankles?—*A.* I have.

*Q.* Was the result successful?—*A.* Perfectly successful, so far that the case was comparatively fit to go out in a few days.

*Q.* Was that, do you remember, a case of Mr. Gayner's?—*A.* Yes, it was.

*Q.* Did you ever find in cases of children whose bandages you cut that complaints were made that they had been cut.—*A.* No. There was no complaint made to me of having cut them.

*Q.* Or that having cut the bandages, they had to be put on again?—*A.* No, they were never put on again after I had cut them. The method adopted in this hospital is the first time I have seen it.

The Commissioner: That is the point I want to inquire about. Your evidence is that there is a right and a wrong way of tying children down if they have to be tied? Dr. Evelyn has demonstrated an apparatus which perhaps you think satisfactory. I have handed it to you for that purpose that you might give an opinion on it. Have you had an opportunity of looking at it?—I have. But I should like to see the range of movement the child had with the apparatus.

*Q.* You don't commit yourself to any statement about it?—*A.* No.

*Q.* The real point is this, as a matter of fact, is the apparatus that is used in the hospital of this character?—*A.* I have never seen it before.

*Q.* During the time you were Resident, whatever children were tied down were not tied down or secured by that apparatus?—*A.* Yes.

The Commissioner (to Dr. Macqueen): What do you say to that?

Dr. Macqueen: I have seen it used by the present sister, but never by the sister to whom the complaints refer.

Mr. Watson (to Dr. Shepherd): You say you cured a case of eczema on a child without any apparatus?—Yes.

*Q.* Where was the eczema of that child?—*A.* From the crown of its head to the sole of its foot.

*Q.* How many children were there in the ward at that time?—*A.* The ward was full.

The Commissioner: Of course, Dr. Evelyn's evidence must relate specially to the cases that were under his own care. He cannot possibly know what particular method is used——

Mr. Baker: I want to ask Dr. Macqueen: In the cases where you cut the bandages did you find any cotton wool as Dr. Evelyn described round the ankles or wrist?

Dr. Macqueen: There was no cotton wool round the ankles or wrist.

Mr. Baker: In any of the cases?—In any of the cases.

Mr. Gayner: I have had a number of my cases kept in bed in this way.

The Commissioner: By that method? There are two ways.—I have never had it used except it was so arranged that the child could sit up in bed. It is to prevent it falling over the side. I have never had them fastened down on the Medical side. When I was on the Surgical side, immediately after an operation I had the child kept down.

*Q.* You will agree that a method of bandaging by means of the shoulders, wrists, and ankles is a method which should only be resorted to in very exceptional cases?—*A.* I have never seen it used on the Medical side.

### THE COMMISSIONER ASKS FOR EXPLANATIONS.

The Commissioner: I am afraid I must ask your attention, Dr. Macqueen and Dr. Shepherd, to a paragraph in this letter which I have already read. It is the undated letter, but it is referred to as of the 27th December 1912. The paragraph is this:—

"We may add that the general question of the nursing staff requires serious consideration, and if the general public became aware of the callous ineptitude of the head of the nursing staff it would demand the resignation not only of the defaulting individuals, but also of the House Committee, who must be held responsible for the corrupt administration. (Here again we must refer you to the Medical Board.)"

Of course to charge a governing body of a hospital with corrupt administration requires some explanation.

Mr. Baker: It is strong language.

The Commissioner: It is strong language even for young men.

Mr. Procter: Which The Hospital were judicious enough not to print.

Mr. Baker (to Dr. Macqueen): When you referred to "callous ineptitude," were you referring to anything which happened in the hospital?

Dr. Macqueen: I was.

*Q.* What incident were you referring to?—*A.* I was referring to the case of Collier, a man who was found alive in the mortuary.

The Commissioner: I did not raise the question because I had in my mind quite clearly that probably Dr. Macqueen's explanation of "callous ineptitude" would refer to the case we are coming to, but it is really rather serious that he and his colleagues should speak of the House Committee as being responsible for corrupt administration. "Corrupt" is not a word to use unless there is something very serious.

Mr. Baker: Have you any explanation you would like to give as to the use of the word "corrupt" with regard to any particular persons? I don't want to suggest anything.

Dr. Macqueen: Well, an explanation could be given, but I would rather not. This part was left out of The Hospital purposely. We did not send it to The Hospital, and the letter, of course, written to the House Committee was a confidential letter.

Mr. Baker: If it becomes a question of discretion, under those circumstances I should not go into it.

The Commissioner: I think you would probably advise them to say nothing more.

Mr. Baker: Certainly.

The Commissioner: I think we might pass from that. Then we come to the case "A Forecast and Warning" on page 646. That we need take no note of, I think, and there is a "Final Appeal to the Committee" which raises no new points. The next question is this question of a patient who was removed from the mortuary.

Mr. Procter: May I interrupt for one moment? As there is a general comment, I desire to point out to you that this so-called appeal of January 11 was sent after the two Residents had received notices that their engagements would be determined. I have no doubt you will appreciate the significance of that.

The Commissioner: It appears to me to be more important to get at the truth than the particular circumstances in which a thing is written. It may be highly desirable to recognise the fact, but I don't think it will alter the course of our inquiry.

Mr. Procter: No; but it will be taken notice of.

### PATIENT WHEN ALIVE REMOVED TO MORTUARY.

The Commissioner (reads): "(1) On September 22, 1912, a patient named Collier was discovered about 11.30 A.M. by the House Physician in the mortuary whilst still alive. A ticket pinned upon the shroud had written upon it that the patient died at 7.30 A.M., and yet at 7.50 A.M. the House Physician was asked concerning the case, and gave his orders thereanent, which orders, he afterwards found, were not carried out; nor was he informed of the 'death' until after the patient had been removed to the mortuary.

"Upon the discovery of this fact we decided to remove the patient to the Isolation Ward, and informed the Matron of our decision, but were met with the astounding reply, 'What advantage is there in having him removed now?'

"The patient ultimately died in the Isolation Ward at 4.45 P.M."

And then, I think, that case is dealt with in other letters. This is a letter of September 27. It is addressed by Dr. Shepherd to the House Committee, I presume; I am not sure:

"*Re* Collier, who died Sunday, the 22nd, at 4.45 P.M. in the above hospital. Collier suffered from chronic nephritis, and for some days previous to his death had manifested slight symptoms of uræmic coma. On Sunday, September 22, at 7.50 A.M., Nurse Hunt came to my bedroom and said Collier did not look so well, and that his pulse was failing. Knowing his condition, I ordered him a hot pack. At 8.5 A.M. Sister Hutchins came to me with a similar report, and I told her I had ordered a hot pack, after which I heard nothing further until 9 A.M., when the porter informed me that Collier was dead and had been removed to the mortuary.

"At 11.45 I went over to the mortuary to have a look at the body, which was laid out in the usual manner. I was struck by the warmth and general appearance of the body, and led to make further examination, which fully convinced me that Collier, though in deep coma, still lived.

"I immediately summoned Dr. Macqueen, who at once recognised that life was not extinct.

"Together we removed Collier to the Isolation Ward, where, however, he did not improve, and finally expired at 4.45 P.M.

"The wife had already been informed of his death, but I had little difficulty in explaining to her the real situation, and as she did not, to my knowledge, know he had been in the mortuary, she expressed herself as quite satisfied, and a death certificate was duly given to her."

There is another letter, but unless you desire it read I won't read it.

Mr. Baker: No; it is Dr. Shepherd's case. You don't require me to take him over particulars?—One general question on it. Is that a correct account, Dr. Shepherd, of what took place that day?

Dr. Shepherd: It is; quite.

Mr. Watson: There is one question strikes me as important. You wrote a letter with regard to this—the letter that has been read by Sir Cooper?

Dr. Shepherd: Yes.

*Q.* And you did not say anything in the letter about a very important point; that is, that you told the Matron about it, and she said, "Never mind," or words to that effect?—*A.* I did not.

*Q.* Why did you leave that out? It is a most important part of the case.—*A.* I left it out of the letter because the letter was a report, really, upon the condition of the man and finding him. My own feelings at the time were not turned to those things. They had nothing to do with the point of the man being there.

The Commissioner: Who was this report written to?—That really went to Mr. Gayner.

*Q.* It was not sent to the House Committee?—*A.* It was a report to him as Honorary-in-Charge.

Mr. Baker: Of course, I have not seen both letters. Was the letter from which this was omitted the letter in which you reported to the Honorary-in-Charge?

The Commissioner: Yes; it is the letter to the Honorary Physician, and, of course, there was no need in reporting the professional circumstances of the death to say anything about the Matron's conduct.

Mr. Baker: You were not then making any complaint?—No; I simply wrote that as a statement of what occurred.

### DR. SHEPHERD EXAMINED.

Mr. Procter: Now, Dr. Shepherd, I want you, in the first place, to explain how it was that at 7.50, and again at 8.5, when you were called to this case and told that it was in a serious condition, that you did not get up and see to it. First of all, at 7.50, Nurse Hunt came to you?—Yes.

*Q.* What did she say to you?—*A.* She said that this man did not look quite so well, that he had shown signs of his pulse failing a little, and of looking as if he were going to have a fit of some kind. Knowing his exact condition—as I had seen him, I should say, between 2 and 3 A.M.—I ordered him a hot pack.

*Q.* But Nurse Hunt told you he was dying?—*A.* She had not told me he was dying.

*Q.* In about a quarter of an hour the Night Sister came?—*A.* Yes.

*Q.* And what did she tell you?—*A.* She simply told me the same thing. She was then going off. She told me the exact facts Nurse Hunt said. She told me this man Collier did not look quite so well, that he had symptoms of looking as if he was going to have a fit, and that his pulse was failing.

*Q.* What exact orders did you give about the hot pack?—*A.* I said to Sister that I had ordered a hot pack, and that was to be got on; the man was in a serious condition, and that not very much could be done for him.

*Q.* What I want you to explain, if you can, is why didn't you go and see the man?—*A.* I did not go and see the man until I had allowed such time as a hot pack could be given.

*Q.* When did you go to see him?—*A.* I would have gone when I was told by the porter that he was dead and had been removed to the mortuary.

*Q.* What time was that?—*A.* That would be between 9 and 10 o'clock.

*Q.* Then you consider that you were discharging your duty by remaining in your room and giving orders?—*A.* Yes, in this case I do.

*Q.* Don't you consider that if you had seen him this incident in all probability would not have happened?—*A.* I do consider that, but I consider another point as well; the orders which I gave were not carried out.

*Q.* Do you mean that the hot pack was not given?—*A.* It was not.

*Q.* When did you first hear that?—*A.* I was told.

*Q.* When?—*A.* Afterwards.

*Q.* After he was supposed to be dead?—*A.* After he was supposed to be dead.

*Q.* What was the reason given as to why they were not carried out?—*A.* Someone must have taken it upon themselves to say the man was dead.

*Q.* Well, now, what was the time when you went into the mortuary?—*A.* Between 11.30 and 11.45.

*Q.* Did you go in consequence of anything that was said to you?—*A.* I did not. I went in consequence of the fact that I was going to do some work down there, and also of the fact that I always see a dead body before I sign the death certificate.

*Q.* Was it the custom at the time for a medical man to see a dead body before its removal to the mortuary?—*A.* I could not say. I had only been there a fortnight or three weeks.

*Q.* You would say it was desirable?—*A.* I should.

*Q.* You did not do it?—*A.* I did not, because I had not any intimation.

*Q.* You went to the mortuary and found him there, and, as you have told us, you came to the conclusion that he was alive?—*A.* Yes.

*Q.* What were the indications?—*A.* His heart continued to beat, and he had a pulse at the wrist; although it was extremely weak, nevertheless it was a pulse.

*Q.* Did you take any other steps to find out?—*A.* I did. I called Dr. Macqueen, and he made an incision over the radial artery, with the result that whenever the light touched the skin there was the usual result. There was a steady pumping of blood, which could only have occurred in a living person.

*Q.* Is it not possible for that to occur sometimes in the case of a dead person?—*A.* Not in the case of a person who has been dead, according to the statement which I could prove to you, for the period I was told.

*Q.* Do you say that he was alive? I suppose that he was in a state of deep coma?—*A.* He was.

*Q.* Was it possible that he might have awakened?—*A.* It was possible.

*Q.* Assuming that he was alive?—*A.* There is no assumption about it.

*Q.* You are quite certain?—*A.* I am.

*Q.* Was it possible that he might have awakened?—*A.* It is quite possible that he would have lived, but, of course, you don't appreciate the treatment possibly of uræmic coma, and probably you don't know that cold does not improve the condition.

*Q.* When you say it was possible he would have lived, what do you mean?—*A.* He might have lived for another twenty-four hours; he might have lived for another forty-eight hours. You can't speculate on these things.

*Q.* Then can we go back to this—that if you had seen him at eight o'clock in the morning he probably would have lived twenty-four hours?—*A.* I don't admit that.

*Q.* I put it to you, it is possible?—*A.* All things are possible.

*Q.* It is probable?—*A.* All things are also probable.

*Q.* Is this probable?—*A.* It may or may not.

*Q.* You are not answering my question. You are really fencing. I ask you is it probable that if you had seen him yourself at 8 o'clock in the morning, when you were called twice, that he might have lived longer?—*A.* It is probable that he would have lived some hours longer, at all events.

*Q.* You opened this artery and you also told us that the heart was beating and the pulse working?—*A.* Yes.

*Q.* What was the need for opening the artery if you found the heart beating and the pulse working?—*A.* The peculiar circumstances of the case demanded that there should be absolutely no doubt about the fact.

*Q.* Of course, I am not a medical man, but surely if the heart is beating, is not that——?—*A.* It is an indication.

*Q.* Isn't it a sufficient indication?—*A.* It is quite a sufficient indication, but you had better have something to substantiate your facts upon.

*Q.* It didn't seem to be necessary here?—*A.* It was not necessary.

*Q.* On that point, it didn't seem to be necessary to open the artery if the heart was beating?—*A.* It was not. But you have to convince some people.

*Q.* I want to know what steps you took when you came to the conclusion that he was alive?—*A.* I immediately told Dr. Macqueen.

*Q.* What conclusion did he come to? Did he agree with you?—*A.* He perfectly agreed with me.

*Q.* And what did you do?—*A.* We went immediately up to see Matron and to see that the man should be put in the Isolation Ward and have some treatment carried out, as it was not possible to carry him back to the ward.

*Q.* Why?—*A.* It was not considered advisable for the sake of the other patients.

### REMOVAL FROM MORTUARY.

*Q.* How long was it before you got him moved into the Isolation Ward?—*A.* I should say between twenty minutes and half an hour.

*Q.* What time would it be when he was removed?—*A.* That would be between 12 and 1 o'clock.

*Q.* Who actually removed him?—*A.* Dr. Macqueen and myself.

*Q.* Had there been any arrangement that you should do that?—*A.* We arranged it ourselves.

*Q.* You two?—*A.* Yes.

*Q.* What time do you actually say it was?—*A.* Between 12 and 1 o'clock, possibly 1 o'clock, I should say.

*Q.* Was it before you had your lunch or after?—*A.* Before.

*Q.* You say about 1 o'clock?—*A.* Yes.

*Q.* Well, I understand that he was in the mortuary still at 1.30 while you were actually at lunch. Now, is that correct?—*A.* That is not. Emphatically no. Dr. Macqueen will probably remember the facts.

*Q.* All right. I am only putting it to you. I shall have some evidence to call. Who helped you to take away the body from the mortuary?—*A.* Dr. Macqueen.

*Q.* Was anybody else there?—*A.* There was nobody else there when we removed him, but the persons who saw him were Sister Guy, Sister ——, also Sister Chapman.

*Q.* Was Sister Guy there at the actual removal?—*A.* She was there; she was there in any case.

*Q.* I think I have already asked you if Nurse Hunt told you in the morning that the man was dying?—*A.* Yes.

*Q.* What do you say?—*A.* She didn't tell me he was dying. She didn't put it in that wording; at least, I took it not. I was told that he showed signs of a fit, but I was not told that he was dying.

*Q.* Before bodies are removed from the wards, there are certain preparations to be made, I understand?—*A.* Yes.

*Q.* Do they take some time?—*A.* Yes.

*Q.* Do they consist partly in the patient's nose being wrapped in cotton wool? *A.*—There were bundles of cotton wool in the nostrils, but they were not put in with any power, and when I got there they were half out.

*Q.* Was the mouth also tied up?—*A.* The mouth was not tied up.

*Q.* When you found it in the mortuary?—*A.* No.

*Q.* It is a bit difficult to understand how a man can breathe and be alive with that in his nose?—*A.* He could breathe through some cotton wool; there is no great difficulty. His mouth was open, and that was sufficient.

*Q.* I suppose it is very difficult indeed for anyone, even for a medical man or surgeon, to tell whether a man is actually dead or alive in this state of coma?—*A.* It is difficult under certain circumstances, and to those not accustomed to seeing many cases of such a nature a difficulty might be there.

*Q.* There might be, but you have no doubt he was alive when you saw him?—*A.* Certainly, I have no doubt whatever.

*Q.* Had there been a bandage round the mouth at all?—*A.* Not to my knowledge.

*Q.* It was not there when you went into the mortuary?—*A.* I didn't see it; it might have been put on in the wards, and in removing the man, it might have come off and got amongst the sheets; for that fact I cannot speak.

*Q.* Was there any possibility of this man living beyond a certain limited time?—*A.* No possibility of his living beyond a limited time, so far as a medical man could assume.

*Q.* I understand there was some suggestion of giving him in the mortuary a hypodermic injection?—*A.* Yes.

*Q.* Is that correct?—*A.* Yes.

*Q.* What was your idea? What sort of injection?—*A.* To stimulate him—to stimulate his heart a bit.

### DOCTOR'S ORDERS NOT CARRIED OUT.

The Commissioner (to Mr. Baker): Do you wish to put any questions to Dr. Shepherd?

Mr. Baker: I think I must.

The Commissioner: I suggest that you should put them, and then we will adjourn.

Mr. Baker: I must do so, although it may be travelling over ground already covered. As far as I could follow it, there were suggestions in the course of the investigation that amounted almost to a countercharge of negligence against Dr. Shepherd. As I understand it, Dr. Shepherd, your first complaint in this case is that you were never told of the serious condition of this man?—Yes.

*Q.* That when information was brought you at 7.30 in the morning of his condition, you gave certain orders?—*A.* Yes.

*Q.* That those orders were never carried out?—*A.* Yes, that is so.

*Q.* No one informed you that the body had been removed to the mortuary?—*A.* No, except the hall porter.

*Q.* Well, I am coming to that. I mean nobody in the ward.—*A.* No.

*Q.* You learnt only casually from the porter that the body had been removed?—*A.* Yes.

*Q.* Were you surprised at that?—*A.* I was.

*Q.* I think a suggestion was thrown out that had you seen the man at 7.30—— What time did you retire to rest the night before?—*A.* I should say between two and three o'clock. I had been around in the wards about that hour.

*Q.* And you were awakened at 7.30 in the morning?—*A.* At 7.50.

*Q.* Had you then got up out of bed and gone into the ward to see this man, was there anything else that you would have ordered to be done except a hot pack?—*A.* Nothing.

*Q.* Then I understand there was some suggestion made about this man being dead? Have you the slightest doubt whatever that he was alive in the mortuary?—*A.* No doubt whatever. He was alive.

*Q.* I think there was some suggestion about your being at luncheon?—*A.* Yes; when he was removed.

*Q.* Is there any truth in that?—*A.* No.

The Commissioner: Is the suggestion that they left him—that having found him alive in the mortuary, they left him and went to lunch?

Mr. Procter: Yes, that is so, Sir.

Mr. Baker: When did you go to lunch?—After we had removed him and seen that he had the necessary accommodation and he was in bed.

*Q.* In bed—where?—*A.* In the Isolation Ward.

The inquiry was then adjourned for luncheon.

On resuming, Mr. Baker said: There is just one question I should like to put to Dr. Shepherd in re-examination. It is in evidence, I think, that on the morning of September 22, 1912, at 7.50, the nurse reported to you that Collier's condition was more serious?—Yes.

*Q.* That at eight o'clock the Sister corroborated that statement?—*A.* Yes.

*Q.* I wanted to ask you this. When you first found him in the mortuary, what time was that?—*A.* I found Collier in the mortuary between 11.30 and a quarter to twelve.

*Q.* Did you find anything pinned on him?—*A.* I did.

*Q.* What was that?—*A.* This ticket which I hold which says that Collier had died at 7.30 A.M.

Mr. Baker: That is all, thank you.

The Commissioner: That is the ticket you actually took from the man?—Yes.

The Commissioner: We have concluded the examination of Dr. Shepherd, and we are now going to ask Dr. Macqueen's account.

Dr. Macqueen was examined by the Commissioner.

*Q.* Was it some time in the course of the morning that Collier's case was brought to your notice? About what time?—*A.* Yes; it was between 11.30 and 11.45. Dr. Shepherd informed me that he had seen this patient in the mortuary and he was still there. I accompanied him to the mortuary and took a knife with me, and there I found the patient and found the heart beating.

*Q.* Did you find the heart beat by putting your hand on the chest, or by the pulse?—*A.* The pulse could be felt very slight, but it could be felt. I cut the radial artery to corroborate that he was alive, and also for treatment to allow a quantity of blood to escape.

Mr. Procter: Did you notice that there were any plugs of cotton wool in the nose?

Dr. Macqueen: As far as I can remember there were no plugs of cotton wool in the nose, but I think Dr. Shepherd had probably taken them out.

### BEFORE OR AFTER LUNCH?

The Commissioner: Later, did you assist Dr. Shepherd to remove Collier to the Isolation Ward?

Dr. Macqueen: Yes.

The Commissioner: Before you had lunch?

Dr. Macqueen: Yes, before lunch.

The Commissioner: You removed him to the Isolation Ward as soon as it was possible to do it?

Dr. Macqueen: He was in the Isolation Ward before a bed was procured.

Mr. Procter: Can you tell us what time he arrived in the Isolation Ward?

Dr. Macqueen: I do not remember that.

Mr. Procter: Can you tell me about what time?

Dr. Macqueen: Before lunch.

Mr. Procter: What does that mean?

Dr. Macqueen : Before one o'clock.

Mr. Procter : He was in the Isolation Ward before one o'clock?

Dr. Macqueen : Yes.

The Commissioner : Was your lunch a fixed meal time?

Dr. Macqueen : Yes; this was Sunday, and we had fixed hours then.

Mr. Procter : Have you any more evidence on this case?

Mr. Baker : Not on this case.

The Commissioner : We should like to have everybody connected with this case, including the Porter who took the body down.

Mr. Procter : I should like to know how Dr. Shepherd comes to be possessed of that ticket now (producing ticket taken from Collier in the mortuary).

Dr. Shepherd : I took it off the sheets.

Mr. Procter : Why did you take it away from the hospital?

Dr. Shepherd : Well, if you had to deal with anything under similar circumstances would you not have removed the chief piece of evidence?

Mr. Procter : It was belonging to the hospital.

Dr. Shepherd : It might have belonged to the hospital, but it belonged to me to prove my statements.

Mr. Procter : You did take it away?

Dr. Shepherd : I did.

The Legal Assessor : I should like to ask you this with regard to this man. He must have died anyway in a short space of time.

Dr. Shepherd : He would have died.

The Legal Assessor : In a short space of time? Clearly understand me. I am not trying to extenuate in any way; I am only wanting to know for my own information.

Dr. Shepherd : He would have died.

The Legal Assessor : He felt absolutely nothing?

Dr. Shepherd : I don't suppose he did.

The Legal Assessor : There is no question about it?

Dr. Shepherd : I cannot answer that.

The Legal Assessor : Have you any doubt, as expert?

Dr. Shepherd : I do not suppose so.

The Legal Assessor : But if such a thing is true that he was taken to the mortuary in a state of coma, he never felt anything at all? He never knew anything?

Dr. Shepherd : I do not suppose so.

The Legal Assessor : That is what you want us to believe?

Dr. Shepherd : I believe it is the general thing.

Mr. Baker : I should like to ask Dr. Macqueen if he has ever had a case which lived?

Mr. Procter : After being taken to the mortuary?

Mr. Baker : In a similar case of coma.

Dr. Macqueen : Yes; the case was treated by a hot pack and venesection.

The Commissioner : Of course it is an experience with all doctors that a patient in a state of coma may recover.

Dr. Macqueen : From what I saw of Collier, I should think it quite possible that if he had been treated he would have recovered.

### NURSE BEWLAY CALLED.

Nurse Bewlay was then called and examined by the Commissioners.

*Q.* Will you tell us, Nurse Bewlay, about this unhappy case of Collier? Tell us all you know about it in your own way.—*A.* From the morning?

*Q.* Yes.—*A.* The first thing I did was I took the patient's temperature.

*Q.* And what was it?—*A.* I cannot remember it; I think 97.4; something like that, sub-normal.

*Q.* What time did you take his temperature?—*A.* About 7.10, as near as I can say, and then I washed the patient.

*Q.* You washed him in the ordinary way as a ward patient? No question of a pack or anything of that sort?—*A.* No. And then I made his bed and had gone away from his bed. I think we had made another patient's bed. I am not quite sure what I did, but I turned round and noticed that Collier was cyanosed, and then he immediately became convulsed. I sent for the Night Sister, but she could not be found, and I asked the Night Nurse to go for Dr. Shepherd.

*Q.* What time would that be?—*A.* As near as I can say about ten minutes to eight.

*Q.* About 7.50 the patient became cyanosed, and you thought he was going into convulsions?—*A.* Yes.

*Q.* Was that the time you sent for Dr. Shepherd?—*A.* Some few minutes elapsed before the night sister could be found.

*Q.* And then you sent for Dr. Shepherd?—*A.* Yes.

*Q.* What time would the message reach him?—*A.* About 7.55.

*Q.* Do you know anything about that ticket which was pinned on to that sheet?—*A.* I do not remember it; it is the first time I have seen it.

*Q.* Is it your writing?—*A.* No.

*Q.* Have you any idea whose it is?—*A.* Sister Chapman's, I believe.

*Q.* She was sister of the ward?—*A.* Yes.

*Q.* Is that an ordinary sort of ticket which comes down with a body?—*A.* Yes.

*Q.* Because obviously if the ticket is genuine it says that Thomas Collier died at 7.30, and you have already accounted for sending for Dr. Shepherd at 7.55?—*A.* Yes.

The Commissioner : And he certainly was not dead then?

Nurse Bewlay : No, Sir.

The Commissioner : Now carry it a little further. You sent for Dr. Shepherd and I suppose got certain orders. What were they?

Nurse Bewlay : He said he could do no good if he came, and told us to put the patient in a hot pack.

The Commissioner : Who did the hot pack?

Nurse Bewlay : It was not given, Sir. It was all got ready and not given.

The Commissioner : Why was it not given?

Nurse Bewlay : Because the patient appeared to die.

The Commissioner : You were there when the patient appeared to die?

Nurse Bewlay : Yes.

The Commissioner : And who else?

Nurse Bewlay : By that time the Night Sister was on.

The Commissioner : Then you had Sister Chapman there, yourself, and the Night Sister. Who is the Night Sister?

Nurse Bewlay : Sister Hutchins.

The Commissioner : And you all thought that the patient was dead?

Nurse Bewlay : Yes.

The Commissioner : You then laid the patient out?

Nurse Bewlay : Yes; we began almost immediately.

The Commissioner : And what time did you finish laying him out? We have got so far as this; we reached 7.55, when Dr. Shepherd was told of the condition of the patient, he tells you to give him a hot pack, and that message has to come back, and then the patient dies and you begin to lay him out almost at once, satisfied that he is dead. When did you complete laying him out?

Nurse Bewlay : I believe about nine o'clock.

The Commissioner : As long as that; that is the best part of an hour?

Nurse Bewlay : Yes; of course we had to get him ready for his hot pack, and he was stripped ready for it.

The Commissioner : But that facilitated your subsequent proceedings? Have you any knowledge who sent for the Porter to take him to the mortuary? I suppose they are taken by a porter?

Nurse Bewlay : I think I sent word.

The Commissioner : And what time did the Porter take him to the mortuary.

Nurse Bewlay : Between 9 and 9.30.

The Commissioner : Is it your custom to get bodies out of the ward very quickly after death?

Nurse Bewlay : Well, no; we leave them for an hour.

The Commissioner : In this case you hardly left the body so long?

Nurse Bewlay : No, we started immediately.

*Q.* And from that point, of course, you know nothing more of the case? After the body went to the mortuary you were finished with him; and is it a fact that you were all satisfied that he was dead, you three ladies?—*A.* Yes.

### PATIENT'S NOSTRILS PLUGGED.

The Commissioner: Did you put your hand upon him?

Nurse Bewlay: Yes, sir, and I felt his pulse. I could feel his pulse when it first started; it was just a flicker and then afterwards nothing. He had had some hæmorrhage for a few days before, and I plugged his nostrils well and his mouth. He had been vomiting a good deal of the blood that he had swallowed.

The Commissioner: I see. So when you sent him to the mortuary, or when he was sent down, his nostrils were well plugged and his mouth full of cotton-wool?

Nurse Bewlay: Yes.

The Commissioner: And the object of that was to check the hæmorrhage from his nose?

Nurse Bewlay: Yes.

The Commissioner: Did you put your hand over his chest?

Nurse Bewlay: Yes, Sir.

The Commissioner: And you could feel no pulsation?

Nurse Bewlay: No, Sir.

Mr. Procter: Did you send more than one message to Dr. Shepherd?

Nurse Bewlay: No, Sir.

Mr. Procter: Do you know anything about a second message?

Nurse Bewlay: The sister remained all the time, and came to the mortuary and was going to send up.

Mr. Procter: About 8 o'clock in the morning?

Nurse Bewlay: I do not know.

Mr. Procter: You only know about one message?

Nurse Bewlay: Yes.

### NURSE HUNT CALLED.

The Commissioner: We are now inquiring about this poor fellow Collier. You were in the ward with the nurse who has just given evidence, were you not?—I was the night nurse.

*Q.* What share had you in the business? What did you do?—*A.* I went for the doctor.

The Commissioner: Do you know what time the nurse sent you for the doctor?

Nurse Hunt: I asked her if I should go.

The Commissioner: What time did you go?

Nurse Hunt: It would be between ten and five minutes to eight.

The Commissioner: Dr. Shepherd did not come himself; he gave directions. Is that so?

Nurse Hunt: Yes.

*Q.* What did he tell you to do?

Mr. Procter: May we have first what she told him?

Nurse Hunt: I said that the man was taken very ill, and looked as if he were dying.

*Q.* And the doctor did not come with you; he gave instructions. What did he tell you?—*A.* The doctor said that he would not get up, but the patient was to have a hot pack.

*Q.* And you came back with that message to the ward?—*A.* Yes.

The Commissioner: Did you help to prepare for the hot pack?

Nurse Hunt: No.

The Commissioner: Did you see him when he was apparently dead?

Nurse Hunt: I saw him when he looked as if he were dying.

The Commissioner: What time did you go off duty?

Nurse Hunt: I had nothing to do with him after 8 o'clock.

The Commissioner: You cannot follow it any further than that?

Nurse Hunt: No, Sir.

Mr. Procter: Did you go more than once to Dr. Shepherd?

Nurse Hunt: No, Sir.

### SISTER HUTCHINS CALLED.

The Commissioner: How about this poor fellow Collier? What had you to do with the case?—I was on night duty.

*Q.* I understand that Nurse Bewlay found out about 7.50 he was getting cyanosed and that he would very likely be convulsed. She then sent for you as the night sister, but you were not immediately forthcoming, and she then sent the night nurse to see Dr. Shepherd. Is that so far right?—*A.* Yes.

The Commissioner: And what happened after you came on the scene?

Sister Hutchins: At 8 o'clock I came into the ward quite by accident and found they had been looking for me. I went to Ward 3 and met Nurse Hunt, and she told me that Collier had had a fit or something, and as she could not find me she had been to Dr. Shepherd, and he ordered a hot pack. I went to the patient's bed and found him, as I thought, sinking very quickly, and as they were getting the hot pack I thought something might be done, and went to Dr. Shepherd and told him that Collier was dying, and his people lived somewhere below York, and if anything could be done for him.

The Commissioner: You were trying to keep him alive till they came?

Sister Hutchins: Yes.

The Commissioner: He said he was dead, sister?

Sister Hutchins: He said he was as good as dead. I said, could nothing be done; could brandy be given him? He said no, nothing could be done; he expected him to go like that. He said he had told Nurse Hunt that he was to have a hot pack, and on getting back Nurse Bewlay said Collier was dead. I told Sister Chapman, and she, with myself and Nurse Hunt and a night nurse, all went to the sister's table, where I told her what had happened to Collier, and we all believed he was dead, and I asked if his only address was Copmanthorpe, as I had sent a telegram.

The Commissioner: Did you see him when he was practically dead? Your information that he was actually dead came from Nurse Hunt through Nurse Bewlay?

Sister Hutchins: Yes.

Mr. Baker: You were the day nurse?

Sister Hutchins: No, night nurse.

Mr. Baker: You were going off duty at what time?

Sister Hutchins: 8.15.

Mr. Baker: And one of the last things you did was to go and see Dr. Shepherd and repeat the message about Collier?

Sister Hutchins: No; I went to see him on my own account to see if anything could be done for him, as the man was dying quickly and his people lived some distance away.

Mr. Baker: Are you quite certain that you used the word "dying" at all to Dr. Shepherd?

Sister Hutchins: Oh, yes.

Mr. Baker: Perfectly certain?

Sister Hutchins: I said, "He appears to be dying," and he said to me, "He is dead," and I wondered at the time how he got the information that he was dead.

Mr. Baker: Are you perfectly certain about that?

Sister Hutchins: Perfectly certain.

Mr. Baker: It is the first time it has been suggested. It has not been put to Dr. Shepherd that he said he was dead. His story is that the man was alive until late in the afternoon?

Sister Hutchins: Yes; but I am perfectly certain when I told Dr. Shepherd that he was dying he said, "The man is dead," and I said, "No," and he said, "Well, as good as dead; nothing can be done for him." There was more said about this case, but I did not wait to listen to it.

Mr. Baker: Have you ever had any quarrels with Dr. Shepherd?

Sister Hutchins: None whatever.

Mr. Baker: Never?

Sister Hutchins: No.

Mr. Baker: Always perfectly good friends?

Sister Hutchins: Well, once I told him his behaviour was not what it ought to be; he was rather stupid. But it was in quite a friendly way.

Mr. Baker: When was that?

Sister Hutchins: I have not kept exactly an account of all that I said to him.

Mr. Baker: It was quite a usual thing for you to tell a Resident that his conduct was stupid?

Sister Hutchins: No, it was not usual.

Mr. Baker: Did it make no impression on your memory? You know I am suggesting that you are telling deliberate lies?

Sister Hutchins: Yes.

Mr. Baker: You can realise that?

Sister Hutchins: I know you are trying to make me contradict myself.

Mr. Baker: I am not setting traps, but I say you deliberately invented that 8 o'clock Sunday morning story.

Sister Hutchins: Well, why did Dr. Shepherd say it would be better not to say it?

Mr. Baker: Oh! When did he say that?

Sister Hutchins: There was an inquiry here about this matter.

Mr. Baker: But when did he say that?

Mr. Procter: It is in the report by Drs. Macdonald and Gayner.

The Commissioner: Would you let me see that report?

Mr. Baker: Was this soon after Collier's death?

Sister Hutchins: It was just as Dr. Shepherd was going away.

Mr. Baker: When was he going away?

Sister Hutchins: About a week after, as far as I can remember.

Mr. Baker: Shortly after Collier's death?

Sister Hutchins: Yes.

Mr. Baker: Did you give evidence before Mr. Gayner?

Sister Hutchins: Yes.

Mr. Baker: Did Dr. Shepherd?

Sister Hutchins: I suppose so.

Mr. Baker: You don't know; you were there all the time?

Sister Hutchins: No.

Mr. Baker: I think you told the Chairman what Dr. Shepherd said before that inquiry came on. I should like you to repeat what it was. I understood he tried to agree with you?

The Commissioner: Have we any record of that inquiry, because I do not seem to have it among my papers? I have this letter of Dr. Shepherd's, but nothing more.

### HOUSE COMMITTEE'S MINUTES.

Mr. Procter: If you will refer to the House Committee's minutes, you will find a reference to it.

The Commissioner: Yes, but what was sent me was an extract from the House Committee minutes. Perhaps I may as well read it.

"January 14, 1913 (which, of course, is a very considerable time after the date of the incident).

"Two further letters were read from the Resident Officers in which they referred to the case of a man named Collier, who had inadvertently been removed to the Mortuary in a moribund condition before death had actually taken place. Mr. Gayner had had charge of the patient, and he explained the circumstances to the Committee. The man had suffered from chronic nephritis, and early on the morning of the date of his death, September 22 last, he had a seizure and fell into a state of weak coma. The House Physician, who was in bed, was notified, and he ordered a hot pack, but while the nurses were preparing to administer it it appeared to them that death had taken place. The matter had been reported to the Medical Board and Mr. Gayner had been deputed to hold an inquiry, and as a result a by-law had been added to the Resident Officer's rules, that no death certificate must be issued without an inspection of the body. In the case of the man Collier, it was pointed out that only a medical man was competent to judge if death had occurred, and in order to prevent a recurrence of the incident it was decided to add a new by-law that an inspection of the body must be made before it is removed from the ward. It was not considered necessary to reply to two letters; these were the further letters from the Resident Officers, and the Secretary was instructed simply to acknowledge their receipt."

The Commissioner: Of course, that is a minute of January 14, dealing with an incident of September 22. Mr. Gayner explains to the House Committee, but I have no account of it.

Mr. Neden: These are rough notes of an inquiry held by Mr. Gayner on October 1.

The Commissioner: You did not give those to me?

Mr. Neden: They were simply rough notes of evidence.

The Commissioner: How was it so very long elapsed between this incident and the report of the House Committee?

Mr. Neden: Dr. Macqueen wrote a letter to the House Committee, and the Medical Board had already dealt with it.

The Commissioner: Was it not an incident which would only have been reported to the House Committee?

Mr. Neden: Well, the Medical Board considered they had done all that was necessary at the time.

The Commissioner: Well, I am afraid I cannot examine on this inquiry, as I have no information upon it.

Mr. Baker: There is only one question I can put on this, which I have never seen before.

The Commissioner: I have not seen it.

Mr. Baker: You did not make any statement of this kind about Dr. Shepherd—that he said Collier was dead—before this inquiry, did you?

Sister Hutchins: Yes, to the Matron.

Mr. Baker: Before this inquiry, you did?

Sister Hutchins: Yes, I told Matron.

Mr. Baker: Before Mr. Gayner, I don't see it. You gave evidence before this inquiry?

Sister Hutchins: No, I simply gave the letter and report as I do every morning, and I told her what I said to Dr. Shepherd.

Mr. Baker: It is difficult to pursue this; I thought this was evidence of an inquiry. Did you say nothing at that inquiry of what you have said to-day about Dr. Shepherd having said that Collier was dead?

Sister Hutchins: No.

The Commissioner: You had told the Matron in your usual report on the incidents of the night, and you told the Matron that you had been to Dr. Shepherd and reported this conversation; but when the matter was considered by Mr. Gayner, you did not bring that out in the inquiry which was held by Mr. Gayner and the Secretary?

Sister Hutchins: Dr. Shepherd asked me if I would mind not saying it. He said it would not sound well; we had better not say it.

Mr. Baker: Did the Matron give evidence at this inquiry?

The Legal Assessor: Where did this conversation with Dr. Shepherd take place?

Sister Hutchins: In his bedroom.

The Legal Assessor: But before the inquiry he told you to keep your mouth shut?

Sister Hutchins: No.

The Legal Assessor: Not in those words, perhaps, but meaning the same?

Sister Hutchins: He wished me not to say that he said Collier was dead or as good as dead, and there was nothing to be done for him.

The Legal Assessor: Where did that conversation take place?

Sister Hutchins: On the night round; I cannot exactly say when.

The Legal Assessor: How long before the inquiry?

Sister Hutchins: Only the day before; just when we knew the inquiry was coming on.

The Legal Assessor: During the time that he was doing duty at night?

Sister Hutchins: This case was being talked about a lot, and we were very distressed, and I said to him one day, "Do you want me to say what you said that morning in your room about Collier being dead, or as good as dead, because it does not sound very nice?"

Mr. Baker: You had to give evidence before the inquiry. It was not your business to ask the Doctor what you should say.

Sister Hutchins: He seemed so distressed about it, and he had been up all the night before worrying and writing out papers and he seemed rather worried, and I thought it was not a very nice thing to say.

Mr. Baker : But you told the Matron about it?

Sister Hutchins : Yes.

The Commissioner : Yes, she told the Matron in the ordinary way of the incidents of night duty, which report is generally verbal, and I suppose it is so here?

Sister Hutchins : Yes.

The Commissioner : Of course, we can inquire of the Matron if she has any recollection of having received that information. Of course, there was nobody present at this conversation between you and Dr. Shepherd?

Sister Hutchins : No.

The Commissioner : It was when you met him on the night round?

Sister Hutchins : Yes.

The Commissioner : And nobody was present?

Sister Hutchins : No, but I also saw Dr. Shepherd since, and he said it did not matter if I did say it.

The Commissioner : Do you recognise that writing at all? It was the ticket which Dr. Shepherd states he found on the body of Collier. We hear from another witness that it seems like Sister Chapman's writing. Is it your writing or Sister Chapman's?

Sister Hutchins : It is not mine; it looks like Sister Chapman's.

Mr. Procter : Before you sent to Dr. Shepherd, had you seen Collier yourself?

Sister Hutchins : Yes.

Mr. Procter : And were you satisfied he was then living?

Sister Hutchins : He was living then.

*Q.* There was no question about that?—*A.* No.

*Q.* On your examination?—*A.* Yes.

*Q.* Do you know anything of the report which Dr. Shepherd drew up in regard to this case?—*A.* No; he was making out some statement about it, but I do not know what it was.

*Q.* Did you ever see any statement?—*A.* I saw him writing one, but I did not actually see it. He asked if Nurse and I would sign it.

*Q.* You did not know what was in it?—*A.* No.

*Q.* Did you decline to sign it?—*A.* We said we would not sign it then.

*Q.* And you have not signed it?—*A.* No; I have not signed it.

Mr. Baker : Has this statement been read?

Mr. Procter : I do not know; I have never seen it. The witness says there was a statement drawn up which Dr. Shepherd asked her and Nurse Hunt to sign.

The Legal Assessor : Did she read the statement?

Sister Hutchins : No; Dr. Shepherd had it in his hand.

*Q.* Did he read it to you?—*A.* I do not remember.

The Commissioner : Did you decline to sign it before you had heard it?—I declined to sign it altogether. Dr. Shepherd said he would put that we were willing, but we said we would see about it later on.

Mr. Baker : Do I understand that Dr. Shepherd asked you to sign it before it was read?—No; I declined to sign it before it was read.

Mr. Procter : She declined to sign any statement, is not that so?—Yes.

The Commissioner : Such a statement has never come before the notice of anybody here. Would you (Mr. Baker) prefer to examine Dr. Shepherd in the presence of this witness?

### DR. SHEPHERD RE-EXAMINED.

Mr. Baker : I think it would be better. Dr. Shepherd, you have heard what the Sister has said, that she came to you on this morning. Where were you?—In bed.

*Q.* About what time was it?—*A.* Five minutes past eight.

*Q.* Then it was about five or ten minutes after you first received the message about Collier?—*A.* Fifteen minutes after, by my watch.

*Q.* Do you remember exactly what words were used?—*A.* She told me that she had seen Collier, that Nurse Hunt had been already down to me, and that Collier had some kind of convulsion, and asked was there any message. I simply stated that little or nothing more could be done for the man until the hot pack had been got. I do not think that I stated anything else.

Sister Hutchins : Oh, Dr. Shepherd, you cannot say that.

Mr. Baker : Wait a minute, please. Is it true to say that you said to her "Oh, Collier is dead," or words to that effect?—No. How could I say the man was dead when I had not seen him, and had ordered a hot pack and had had no further communication concerning him?

*Q.* Passing from that incident, it has also been said that before the inquiry held by Mr. Gayner you approached Sister Hutchins with a view to concealing the fact that you had made this confession, as I might call it, about having made this statement that the man was dead?—*A.* There is no truth in it; I never said the man was dead; I did not know.

*Q.* Have you ever discussed what should be said about Collier's case by Sister Hutchins?—*A.* I never discussed what evidence she should give, because I did not know what evidence could be given.

Sister Hutchins : May I ask him a question?

Mr. Baker : Did you draw up a report which you asked Sister Hutchins to sign?—I drew up a report regarding the times Nurse Hunt and Sister Hutchins came to my rooms concerning Collier. I asked them about signing it.

*Q.* Was it before Mr. Gayner's inquiry?—*A.* Yes; I really wanted it for him. As they did not appear willing to sign it, I simply destroyed it and made another. It was entirely in the interest of finding out what had really happened.

*Q.* You did not ask Nurse Bewlay or Sister Chapman to sign it?—*A.* No.

*Q.* Just these two, and as they refused you tore it up?—*A.* Yes.

The Commissioner : And wrote this instead?—Yes.

The Commissioner (to Sister Hutchins) : You said that you wished to ask Dr. Shepherd a question.

Sister Hutchins : I want to ask Dr. Shepherd if he really thinks that he never said that Collier was dead and he could do nothing, and that he expected him to go that way?

Mr. Baker : I have already asked him that.

The Commissioner : Of course there is considerable difference between saying that he is as good as dead and really dead.

Sister Hutchins : He said he was dead first of all, and I said "No," and then he said "Well, he's as good as dead."

### SISTER CHAPMAN CALLED.

The Commissioner : Can you tell us what you had to do with this case?—I came on duty at 8 o'clock, and understood that the patient had been taken ill and had died in a fit at 7.30.

The Commissioner : The evidence we have is that it was 7.50 before he became cyanosed and looked as if he was going into convulsions.—Yes; I understood that he had died after the fit had occurred, but I was under the impression, when I was asking about it, that the patient had died at 7.30.

The Commissioner : Did you record that on that ticket?—Yes, that is my handwriting.

*Q.* But you must have been rather out as to the time?—*A.* Yes; I was very much surprised when I heard it was 7.50. I was taking it from my report that he died at 7.30.

*Q.* When did you come on duty?—*A.* At 8 o'clock, with the other sisters.

*Q.* But surely you must have been in the thick of it, because that was the time when the preparations were being made for this pack which another sister had sent for. You yourself saw the man?—*A.* Yes, he was laid on the bed.

*Q.* And you judged him to be dead?—*A.* I did not take the judgment that he was dead, but I understood that he died at 7.30. I looked at him as the patient of whom I had a report that he had died at 7.30. I did not look at him with the idea that I could do anything more for him, but I had received a report that he was dead, and I went to look at him, but I did not have any idea that I should be asked whether he was dead or not, or that I could do anything more for him.

*Q.* It remains that you were very much mistaken as to the time?—*A*. It seems so, but I was not aware of the fact that morning.

*Q*. You did not come on duty that morning before the laying out?—*A*. He was laid flat on the bed; the laying out had not begun then.

*Q*. It was about 9 o'clock when the laying out was finished, is that right?—*A*. Yes, sir; it would be possibly a little after 9.

*Q*. Did you see the removal to the mortuary?—*A*. Yes, sir; I sent the order for the porter, as I always do.

*Q*. But as a matter of fact, then, you sent the order, but you took it on trust that the man was dead?—*A*. Well, of course, I took it from the night report that the man was dead; I did not question the night report.

*Q*. Did you see Nurse Bewlay, or did you see Nurse Hunt?—*A*. I saw Nurse Hunt first.

*Q*. Was she the night nurse?—*A*. Yes, in charge of the ward.

*Q*. And it was from her report that you learned he was dead?—*A*. Yes.

Mr. Procter: Was that a verbal or a written report as to the death?—It was a verbal report; Nurse Hunt told me he died at 7.30.

The Commissioner: No; she assumes the patient died at 7.30. Will you tell us from whom you got the report?—As far as I understood it, from Nurse Hunt; I always take my report from the nurse on night duty.

Mr. Procter: Was that a written report?

The Commissioner: Oh, no; that was impossible; a nurse going off duty tells the Sister of the incidents of the night; they could not write such a report every morning.

Mr. Procter: Then you mean that Nurse Hunt said he died at 7.30?—I understood it from her.

*Q*. But did she say so?

The Commissioner: I don't suppose Sister can pledge herself as to that.

Sister Chapman: I certainly understood from Nurse Hunt's report that she meant that.

The Commissioner: The best proof that you understood it was your writing of that ticket?—Yes, certainly; I should never have written such a note had I not understood that the man was dead.

### THE MATRON AGAIN.

The Matron (Miss Tute) was then called and examined by the Commissioner. The first question I have to put to you is this: We are told by the night sister, Sister Hutchins, that when she made her morning report to you she told you that she had been to Dr. Shepherd and had asked him to come and see poor Collier, and that he refused to go, saying that Collier was dead.—That is so, sir.

*Q*. Did the Sister tell you anything else, any other details of her interview with Dr. Shepherd?—*A*. That he said he was dead, or as good as dead, and that nothing more could be done for him.

*Q*. So that you understood from her that that was the second visit that Dr. Shepherd had had about that—first that Nurse Hunt had been and got instructions as to a hot pack, and then Sister Hutchins had been and come back with this report, and she told you that Dr. Shepherd had told her that Collier was dead?—*A*. Yes.

*Q*. Can you say definitely whether what she said was that he said he was dead or that Dr. Shepherd said he was as good as dead?—*A*. She said that Dr. Shepherd said "Collier's dead."

*Q*. I am trying to see how the misunderstanding could have arisen, because there is all the difference in the world in saying a person is dead or as good as dead, because that could be said of anybody in a state of coma, but it is extremely difficult to understand how Dr. Shepherd could say Collier was dead when he understood he was not dead.—*A*. I understood that the Sister had said in reply to his remark, "Oh, is he?" That was what the Sister said to me.

*Q*. You see my difficulty; Dr. Shepherd had had a message from Nurse Hunt that this man was very bad, what could be done for him, and then comes the Sister to him, and suddenly, having had no information between, he says "Collier is dead." How could he have had the information that he was dead? He might have said he is as good as dead.—*A*. He might have gone on to say that, but I recollect the first remark quite well.

Mr. Baker: I took down the first answer the Matron made as to what reply Dr. Shepherd made: "He was dead, or as good as dead, and no more could be done"; is that right?—Yes; the first remark was "Oh, he's dead."

*Q*. But it is not the same.—*A*. And then the Sister said, "Oh, is he?" and then he probably went on, the remark is fixed in my mind from the very beginning that Dr. Shepherd said "Oh, he is dead."

*Q*. I am not asking you about probabilities, but I will leave it at that.

By the Legal Assessor: At the inquiry held by Mr. Gayner you gave evidence?—I did.

*Q*. Did you make any statement about this statement made to you by Sister Hutchins?—*A*. I really don't remember.

### MR. GAYNER AGAIN.

The Commissioner: Is Mr. Gayner here?—Yes.

Was any such statement made?

Mr. Gayner: Sister Hutchins made it very clear that she pressed strongly for some treatment, and that Dr. Shepherd said it was of no use.

The Commissioner: But she made no statement that Dr. Shepherd said, "Collier is dead."—No, it was no use.

Can you recollect if the Matron said anything about that?

Mr. Gayner: I do not think that was asked.

The Commissioner: I should like to know exactly how the body got to the mortuary. Was it on Sunday morning?

The Matron: Yes.

### THE PORTER (J. SMITH).

The Porter (J. Smith) was then called and examined by the Commissioner: I only want to ask you at what time of the morning you took down the body of this poor fellow Collier?—I should think about 9 o'clock.

The Commissioner: Nurse thinks it would be about 9.30?—It would be about that time.

How long have you been acting as mortuary porter?—About three years six months.

So that you have removed a good many bodies to the mortuary in that time?—Yes, since I have been here.

Was the body entirely swathed or covered up?—It was all covered up.

So that you had no opportunity of judging whether the man was alive or dead?—No.

The Commissioner (resuming examination of the Matron): I have to read this letter from the residents. After the patient was taken to the mortuary and found to be alive, upon the discovery of this fact, say the residents, Drs. Macqueen and Shepherd, "We decided to move the patient to the Isolation Ward, and informed the Matron of our decision, but were met with the astounding reply, 'What advantage is there in having him removed now?'" The patient ultimately died in the Isolation Ward at 4.45 P.M. That is practically, I think, identical with the account which appeared in The Hospital newspaper. What have you to say to that?—It was some time after one o'clock when Dr. Macqueen came to me, and I was naturally very surprised and very distressed. I recollect asking him at once if the man was conscious, and he said, "Oh dear no," he was practically dead but not legally dead. I have no recollection of any remark about the Isolation Ward. The only remark I can remember is asking if the man was conscious.

### THE MATRON'S REMEMBRANCES.

The Commissioner: When the patient was removed to the Isolation Ward; are the Isolation Wards often in use here?—Not very often.

Are they furnished as wards?—There is furniture there, but we have to take in a bed when it is wanted.

Was it your duty to provide some sort of attendance for the poor fellow during the time that he was in the

Isolation Ward?—We had to see about a mattress being taken in first, and some bedclothes, which I sent at once.

The Commissioner: Was it necessary to send the nurse who was in charge to the Resident at that time?—Owing to the change of Residents, I had not been asked for nurses, and I set about getting things into the Isolation Ward.

*Q.* Your reason for taking him to the Isolation Ward was because it was near?—*A.* Yes, quite near.

*Q.* Obviously you could not take him back where he was supposed to have died.—*A.* I have no recollection, but it was so thoroughly impressed on me that the man was practically dead but not legally dead, and that he was not conscious.

*Q.* Can you give in conversation what happened?—Did you make any remark to the Resident?—*A.* I expect I did. We discussed the thing as to what was the best thing to be done and how best to arrange.

*Q.* This was a conversation with both Residents?—*A.* No, only Dr. Macqueen.

*Q.* Was anybody else there?—*A.* No; he was at my room door. There was someone in the room, but she could not hear.

Mr. Procter: Were you present at the inquiry held by Mr. Gayner and the Secretary?—I came in and gave evidence.

*Q.* Was anything said about this observation?—*A.* Not then. Of course, I set about getting the Isolation Ward ready then, and as soon as it was ready informed the Residents. It was a quarter to two when I informed them.

Mr. Baker: I did not ask about that statement. I think you said it was outside your room that this conversation took place with Dr. Macqueen?—Yes, just at the door.

*Q.* I think you had been speaking to him for a minute or so when Dr. Shepherd came up?—*A.* I do not think so. I think I saw him afterwards only.

*Q.* They say that he came up and they both told you their version, and that you made the reply, "What advantage is there in having him removed now?" in the presence of both of them.—*A.* I could not say now, I only recollect Dr. Macqueen's remark about not legally dead.

*Q.* Are you quite certain about "not legally dead"?—*A.* Absolutely certain.

*Q.* Who said that?—*A.* Dr. Macqueen. I very well remember it because it was a remark I was not familiar with, and which impressed me considerably. Of that word I was absolutely certain.

*Q.* It seems a very peculiar remark for a doctor to make.

The Commissioner: No; because every doctor learns medico-legal evidence of life or death, and what Dr. Macqueen meant was that the man had signs of being alive which would satisfy medical experts, but that he was a dead man as regards consciousness, showing only the animal function of respiration.) If you used the remark I take it that is what you would mean.

### DR. MACQUEEN: I DID NOT USE THE REMARK.

Mr. Baker: You did not use it at all?

Dr. Macqueen: No.

The Commissioner: And it was a very unusual remark indeed to come into the mind of a Matron.

The Matron: It was a remark I had not heard before.

Dr. Macqueen: It was a remark made by the doctor afterwards.

Examined by Mr. Procter:

Mr. Procter: It was shortly after one o'clock when Dr. Macqueen mentioned the matter to you?—Yes.

*Q.* Was that the first you heard of Collier being in the mortuary?—*A.* Yes.

*Q.* Did you know that the body was then in the same mortuary?—*A.* It would be.

*Q.* Did you go there then?—*A.* No, I set about at once getting things ready in the Isolation Ward.

*Q.* Did you get there eventually?—*A.* Not until I went after a quarter to two.

*Q.* Did you see Collier's body in the mortuary?—*A.* No.

*Q.* When did you see it in the Isolation Ward?—*A.* I suppose soon after it was moved there.

*Q.* It was a quarter to two?—*A.* Yes.

*Q.* And when Dr. Macqueen spoke to you the body was still in the mortuary?—*A.* Oh, yes. When the things were ready in the Isolation Ward I told the doctors in the dining-room that the Isolation Ward was ready. It was a quarter to two. I was due upstairs then.

*Q.* Did they say anything to you about it being removed?—*A.* No, they said "Thank you."

*Q.* Did they say they had removed it or not?—*A.* Well, they had not, because I came straight from the Isolation Room to tell them it was ready.

*Q.* It seems to be clear that the body was in the mortuary at a quarter to two?—*A.* Certainly.

The Commissioner: Is the bearing of this question on the luncheon question?

Mr. Procter: Yes.

Mr. Baker: Had you had your luncheon?

The Matron: Yes.

Mr. Baker: You came out of the room at one o'clock?—Yes, and into my room; and I was sitting in the chair when Dr. Macqueen came and told me.

Mr. Baker: I understood you to say that between that time and when you knew the body was in the mortuary you were getting the bed ready?—As soon as Dr. Macqueen told me I set to get things ready.

*Q.* Is that all that was done in that three-quarters of an hour?—*A.* It was not three-quarters of an hour. I had been a little time in my room, and sat there for a short time before Dr. Macqueen came. I do not know the exact moment he came, I could not say.

*Q.* What is there that helps you to remember the time at all?—*A.* I know absolutely, because I had that three-quarters of an hour free time between finishing my lunch and being due in the Chapel to play the harmonium at a quarter to two. That was my only free time, and it was in that time that Dr. Macqueen came. I cannot say the exact moment he came, but it was after one o'clock.

*Q.* My difficulty is to find out how you place it so late as a quarter to two.—*A.* After I finished getting the things ready—I had to get a mattress down from another ward down the fire-escape staircase, and had to arrange the bed-linen, and a little time we were discussing it together as to the best thing to do.

*Q.* And that is how the three-quarters of an hour were filled up?—*A.* It would hardly be three-quarters of an hour.

*Q.* That is what I am suggesting.—*A.* It might have been a quarter or twenty minutes past one. I have not the absolutely correct moment, but I know I had to hurry to get the arrangements made in order to get ready for chapel at a quarter to two. As a matter of fact, the chaplain was waiting for me.

*Q.* It might have been a quarter or twenty minutes past one, or five minutes past one?—*A.* No, I think a little later than that, because I had been sitting in my room.

Dr. Macqueen, examined by Mr. Baker:

*Q.* Do you remember seeing the matron about Collier on this occasion?—*A.* Yes.

*Q.* What time?—*A.* Before one o'clock.

*Q.* Do you remember that clearly?—*A.* Yes.

*Q.* Is there anything which makes you sure?—*A.* It was before lunch.

*Q.* Does the Matron have her lunch at any time?—*A.* I don't know.

*Q.* Do you remember what you said to her?—*A.* I told her that this patient was alive in the mortuary. She asked me if he were conscious. I said, No; he was unconscious, but I did not expect that he would get over it; and she made this remark——

*Q.* What remark?—*A.* "What advantage is there in having him removed now?"

*Q.* At that time was anybody else present?—*A.* Dr. Shepherd was with me at that time.

*Q.* I think the patient died ultimately at 4.45?—*A.* Yes.

The Legal Assessor: How did Dr. Shepherd come to be there? How did you happen to be together when the statement was made by the Matron? You had found the man and went to report to the Matron?—He came up from the mortuary immediately behind me.

*Q.* Did you take him to the mortuary?

The Commissioner: No, it was Dr. Shepherd's case. He had been there, and from that time they had been working together.

By Mr. Baker: You went expressly to tell the Matron?—*A.* Yes.

### DR. SHEPHERD EXAMINED BY MR. BAKER.

*Q.* Do you remember arriving upon the scene when Dr. Macqueen was talking to the Matron about Collier?—*A.* I do.

*Q.* How much of that conversation did you hear? Do you remember?—*A.* I heard Dr. Macqueen discuss the point that the man was alive at the time, but he was unconscious—totally unconscious—and I also heard the remark as to moving the body to the Isolation Ward, and "What advantage is there in removing him now?" I was standing with my back close to the door.

*Q.* Did you hear Dr. Macqueen say "not legally dead"?

The Commissioner: No, he didn't say that. (To Dr. Shepherd) Did you say it?—I said that the man was virtually dead, not legally dead.

By Mr. Baker: Did you say it before or after the Matron's remark about what advantage in moving him now?—Before.

*Q.* Do you know what time it was?—*A.* It would be either one or a few minutes after.

*Q.* How do you fix the time?—*A.* Because I went to the mortuary about a quarter to twelve, and, finding the man alive, tried to get something done for him. That would bring it to that time.

### BEFORE OR AFTER LUNCH?

*Q.* Had you had your lunch? Is lunch a punctual meal on Sunday?—*A.* Not very punctual; it varies fifteen minutes perhaps.

The Commissioner: You are quite clear that you had moved him to the Isolation Ward before you had lunch?—Certainly, because I washed my hands after—before lunch.

*Q.* (by Mr. Procter): Can you fix the time exactly when he was moved?

The Commissioner: Lunch on Sunday is at one o'clock for 1.15 perhaps, and it is clear they got the body to the Isolation Ward before 1.0 or 1.15.

Mr. Procter: How do you account for the Matron's statement that at 1.45 the body was not in the Isolation Ward?—I cannot account for it.

*Q.* Do you say it is untrue?—*A.* I would not go so far as to say it was untrue.

The Commissioner: But it cannot be true if your account is true?—No, it cannot be.

Mr. Procter: Have you any witnesses who will bear out your statement as to the time?—There was no one else present as far as I know.

The Commissioner: Being Sunday, you two gentlemen worked together and took the body yourselves without assistance?—Yes.

### WHY MOVE HIM NOW?

Mr. Procter: With regard to this statement that the Matron said "What advantage is there in moving him now?" when do you say that she said that? After you expressed the opinion that he was virtually, if not legally dead?—I think it was after that, but I cannot be certain. I know I made the remark, and I know I heard the Matron's observation.

Mr. Procter: Assuming that the observation was made and it is not admitted——

Mr. Baker: It is not denied.

Mr. Procter: The Matron said she had no recollection.

Mr. Baker: Quite so.

Mr. Procter: Supposing she did say it, and she said it after you had made your remark, where do you see any callousness?

The Commissioner: The words are, I think, "callous ineptitude."

Dr. Shepherd: I don't consider that a question which can be fairly answered by me.

Mr. Procter: Do you think it was callous?

Dr. Shepherd: No one likes to think of a man lying alive on a stone slab; that is my opinion.

*Q.* At the inquiry by Mr. Gayner did you say that the Matron had made the observation?—*A.* No.

*Q.* Why not?—*A.* Because I was simply asked where I had been and answered the question.

*Q.* I suppose you were there to give an account of the whole affair?—*A.* No, I was not.

Mr. Baker: Were you questioned by Mr. Gayner?—*A.* Yes.

*Q.* Asked questions, or asked to make a statement?—*A.* I cannot trust my recollection.

The Commissioner: I presume you would not be asked many questions because you had sent in this report?

Dr. Shepherd: Just a few questions to support it.

Mr. Gayner: Dr. Shepherd was asked to tell us what he knew of the case.

The Commissioner: As a matter of fact he had written that account. You stood close to the Matron's door during the conversation?—Yes.

The Commissioner: She states that there was somebody in the room at the time. Is that evidence available?

Mr. Gayner: I think the Matron said she did not know who it was.

### SISTER GUY, ASSISTANT MATRON, CALLED.

*Q.* (by Mr. Procter): You know something about the Collier case, Sister Guy?—*A.* Yes.

*Q.* How were you concerned in it?—*A.* Mr. Shepherd reported to me, when I was in the mortuary, that he did not consider he was dead.

*Q.* He reported it to you as Assistant Matron?—*A.* Yes.

*Q.* Did that take place in the theatre?—*A.* No, I was in the theatre at 10.30, when Dr. Shepherd said Collier was dead, and I remarked he must have gone very suddenly, and he said, "Yes, oh yes; he had a fit and went." And nothing more was said.

*Q.* When did you see Dr. Shepherd again?—*A.* He came to my room to report that the man was in the mortuary at twenty minutes to one.

*Q.* Was that after he had been to the mortuary?—*A.* Yes.

*Q.* Did you afterwards see both doctors, Macqueen and Shepherd together, going towards the mortuary?—*A.* It was before then. I had seen them go past the store between a quarter and half-past twelve.

*Q.* You were in your room about twenty minutes to one and Dr. Shepherd came?—*A.* Yes.

*Q.* What did he report?—*A.* He said, "Do you know the man from Ward III.? He has been taken to the mortuary. He is not dead." I then said "Not dead? What did you mean when you said in the theatre that he was dead?" After that he said "Well, he was practically dead, but he could not give a certificate because Dr. Macqueen had cut an artery and he was still alive." I asked him if he had seen him, and he said "No," and I said "How could you say he was dead if you had not seen him?" and he said "I know it is entirely my own fault, and that I should have seen the patient."

*Q.* Did he say anything else?—*A.* That is all I can remember now.

*Q.* Then he asked for some dressings?—*A.* I went to the casualty room and got some dressings, and we both went together.

*Q.* And Collier was there?—*A.* Yes.

*Q.* What was happening then?—*A.* Dr. Macqueen had hold of the patient's artery.

*Q.* Was there some fluid?—*A.* Yes, running to the floor. I asked him if he considered the patient was alive because the patient appeared to be dead.

*Q.* What did Dr. Shepherd say to that?—*A.* He could not possibly be dead as the artery was running, the body was still very warm, and the eyes not dilated.

*Q.* And the limbs?—*A.* Not rigid. I asked him what he was going to do. He said "Only give him a hypodermic," and I said it would be no use taking him to Ward III., because he would be dead by the time we got there. I suggested, as the Isolation Ward was ready, he could be taken there, and he said "Yes." I held the arm while Dr. Macqueen put in stitches and bandaged it up, and later Dr. Shepherd went to the casualty room and I gave him some hypodermic tabloids and a syringe, and by that time Sister Chapman had come in from dinner, and I said to her, "Dr. Shepherd says that Collier is not dead. Will you go and see to the isolation? They want it ready; and get a mattress," and Dr. Shepherd and I left Sister Chapman to go to the mortuary and I went for my dinner.

*Q.* What time?—*A.* Sisters come out from dinner about one, and it would be 1.5 when I spoke to Sister Chapman.

*Q.* Did you go to the mortuary after that?—*A.* Later on I went.

*Q.* What time?—*A.* One-thirty, when I came out from nurses' dinner; then I had to go to the store-room to get stores out, and after that I went to the mortuary, and found Collier still in the mortuary.

*Q.* Are you quite sure about the time?—*A.* Yes, quite sure.

*Q.* How long were you at the stores?—*A.* It would be about a quarter to two.

*Q.* You would get to the mortuary about a quarter to two?—*A.* Yes, between twenty and a quarter to two when I went to the mortuary.

*Q.* And you saw Collier?—*A.* Yes; and I wondered why, if he was considered not dead. I went into the Isolation Ward and saw Sister Chapman waiting, and as the body had not come I went to see the doctors.

*Q.* What did you say to them?—*A.* I inquired in the hall, and the porters said they were at lunch.

*Q.* Did you go into their room?—*A.* Yes, and they were having lunch, but not finished.

*Q.* And the body was still in the mortuary?—*A.* Yes; I left it in the mortuary. I said, "We have not got the body out yet, and the patient's wife is coming at two o'clock, and if they did not come soon the patient's wife would be there before the patient was removed from the mortuary to the Isolation Ward. We all three went at once, and I held the stretcher while the body was put on it, and walked by the side while it was taken to the Isolation Ward from the mortuary.

*Q.* Did Dr. Shepherd make an examination of him at this time?—*A.* When we got to the Isolation Ward he examined his heart, and I asked him if he could hear anything, and he said "Yes, he could still hear a faint flicker, but it was much less than what it was when he listened in the mortuary," and he handed me the stethoscope, and I listened, and I said, "With no imagination can I hear anything."

*Q.* Did you hear anything?—*A.* I could not hear any signs.

*Q.* Did you try the pulse?—*A.* Yes.

*Q.* Then what?—*A.* I asked him if there was anything he wanted doing, and he said "No," and I remained with the patient until Sister Chapman came to relieve me.

*Q.* What time was that?—*A.* Between three-thirty and a quarter to four.

*Q.* You are quite sure about your times?—*A.* Yes, I am quite sure about the times.

*Q.* And quite sure that you left Collier in the mortuary and went to the Residents' room and found them at lunch?—*A.* Yes; I waited there till they came along.

The Commissioner: Before a question is put to you for the other side, you said you were there when Dr. Macqueen opened the radial artery and that you saw some fluid. About how much did it amount to?—When I went, some was running on the floor.

*Q.* The artery was bleeding on to the floor?—*A.* Yes.

*Q.* How much blood was there, would you say?—*A.* One or two ounces, from what I could gather there was on the floor.

The Commissioner: That would be an excessive amount of blood from the radial of a dead man?—Yes.

*Q.* In what position was the arm? Like this? (folding arm across chest)—*A.* No. Mr. Macqueen had it stretched right out from the side.

Mr. Baker: I do not think I quite followed what happened in the morning. You had several interviews with Dr. Shepherd, as far as I can understand. What was the first time you saw him?—I was in the theatre between 10.30 and 11 in the morning. I am on duty in the theatre when the theatre sister is off.

*Q.* And on Sundays you are on duty?—*A.* Yes, in case there are any cases. I take the operations when the theatre sister is off.

*Q.* And you were waiting there?—*A.* I was not waiting there. I was just calling in and Dr. Shepherd came in for something he required for one of the wards.

*Q.* You say you just called in?—*A.* I was in when Dr. Shepherd called in for something he wanted.

*Q.* You were only just come in?—*A.* Yes, that was all.

*Q.* What did Dr. Shepherd say to you?—*A.* He said Collier is dead.

*Q.* Are you quite certain?—*A.* Yes, I am quite sure he said that Collier is dead.

*Q.* Did he come all the way into the theatre to say that he was dead?—*A.* No, it was during the time I was reaching him out from the stock cupboard what he required.

*Q.* What did he require?—*A.* I think it was the tubing that he was using in one of the wards.

*Q.* For Collier's case?—*A.* No, in another ward.

*Q.* He came for tubing?—*A.* Yes.

*Q.* And while you were giving it him he gave the information that Collier was dead?—*A.* Yes.

*Q.* And what was the time?—*A.* Between 10.30 and 11.

*Q.* And what time did you go to the mortuary and find him alive?—*A.* About a quarter to one.

The Commissioner: The importance of the evidence is the question of the luncheon hour—I am quite sure it was a quarter to two.

Mr. Baker: You said that it was on the second occasion or the third that you saw Dr. Shepherd.

*A.* Which do you mean?

Mr. Baker: When he told you about the removal of the body, you placed—as I gather your evidence—you placed the time when you saw the body still in the mortuary—I think you told us it was a quarter to two—you placed that hour in reference to other things done previously?—I came from dinner at 1.30.

*Q.* You cannot say that the clock said a quarter to two?—*A.* It was exactly ten minutes to two when we went across to the mortuary.

*Q.* I understood you to calculate the time by the things you had done before?—*A.* Yes, when going to the doctor's room, but when going from it across to the mortuary it was ten to two by the clock.

*Q.* But if you saw it was ten to two, why the necessity for telling the long story you have done to bring the time to a quarter to two?—*A.* It was a quarter to two when I went to them, and ten to when they came out of the room, because I said it was ten to two.

*Q.* What room?—*A.* Out of the dining-room. I went to the dining-room for them.

The Commissioner: She found them at lunch while the body was still in the mortuary, and she puts the time at ten to two when they finished their lunch.

Mr. Baker: But this was after they had spoken to the Matron?

Sister Guy: I don't know anything about them speaking to the Matron. The Matron went to chapel and I never saw her between one and when I went for them to take them to the mortuary. I did not know that the Matron had been and told them at that time.

The Hospital Secretary: The lady who was present in the Matron's room is the present Matron of the Torquay Hospital.

### A CUSTOM AT YORK.

Mr. Gayner: I think it would be a convenient opportunity to explain to you (the Commissioner) that it is the custom in this hospital for the night reports to be written, and there is a book in each ward in which night reports are written for the day sister. Sister Chapman is not one of our sisters here. She was trained at Edinburgh, but doing local duty, and she was used to a verbal report.

Sister Chapman was recalled, and examined by

Mr. Procter: You remember the isolation ward being got ready for Collier to be taken there?—*A.* Yes, sir.

*Q.* Were you there when he was actually taken into the hospital?—*A.* No.

*Q.* Can you tell us when he was taken there?—*A.* I could not tell you exactly, but I returned to my own ward and came down when I came off duty.

*Q.* How near can you tell us?—*A.* I helped to take the mattress to the Isolation Ward about twenty to two.

*Q.* He was not in the ward then?—*A.* No, because I took the mattress there then—at twenty to two.

*Q.* And is that the last time you were in the ward?—*A.* I returned to it when I came off duty.

*Q.* What time was that?—*A.* Possibly twenty minutes or half past three.

*Q.* He was there then?—*A.* Oh, yes, he was there then.

*Q.* At twenty to two he was not there?—*A.* Well, I did not get the mattress there till then.

*Q.* And you have no doubt about the time?—*A.* Well, I did not leave dinner till one o'clock, and I went to the laundry to see about a mattress and returned to my own ward for blankets and other things, so I am quite sure it would be quite twenty to two when I went to relieve nurse and to apologise to nurse for keeping her so late for dinner.

Mr. Baker: That is how you fix the time, because you had to do all these things?—*A.* Yes, that is how I fix the time with regard to the meals.

### PATIENT WHEN ALIVE REMOVED TO MORTUARY (*cont.*).

Second Day's Sitting, April 19, 1913.

Mr. Gayner examined by Commissioner:—

*Q.* First, I should like to ask you this morning, Mr. Gayner, with respect to the case of the man Collier. You held an inquiry on October 1, as I judge from the rough notes?—*A.* I expect that is correct. These rough notes were made on the day.

*Q.* I do not wish to pin you to the day. I am taking that date from the inscription on the notes.—*A.* That would be correct, then.

*Q.* Then there are certain rough notes there of the evidence about which I shall have to ask you a few words later, and here we find a report of the Committee, and I want to know first whether a fair copy of this has been made, or whether this is all that exists?—*A.* It is all that exists, so far as I know. I do not know of anything else.

*Q.* That report was brought to the notice of the Medical Board. What happened to it?—*A.* The information was given me by Dr. Shepherd that Collier had been found alive in the mortuary on a day on which the Medical Board was sitting, and I handed Dr. Shepherd's report on to the Medical Board. The Board asked me to investigate the matter. I asked for Mr. Neden's help as amanuensis, and on that I drew up that report, which was presented at the next meeting of the Medical Board.

*Q.* So that this report as it stands has been before the Medical Board?—*A.* Yes.

*Q.* That is in accordance, then, with the statement in the House Committee minutes of January 14, that the matter had been reported to the Medical Board. You reported it to the Medical Board, but it came back to you in that way?—

*Q.* You held that inquiry?—*A.* Yes.

*Q.* You reported to the Medical Board?—*A.* Yes.

*Q.* And then made certain recommendations as to alterations of the by-laws?—*A.* Yes.

*Q.* Then I take it that this was your report?

### A REPORT OF AN INQUIRY.

The Commissioner read the report, as follows:—

"*Re* Removal of Living Patient in the Mortuary.

"This incident appears to have come about by reason of a suggestion from the Senior Day Nurse to the Night Nurse that the patient Collier had died. It would not have occurred if either the Day Sister had endeavoured to get a clear report from the night report as to what had occurred, nor if the Senior Day Nurse had obtained from the Day Sister definite instructions to refrain from carrying out treatment ordered by the House Physician. It must be remembered that the facts then investigated occurred seven or nine days before the period of investigation. On the whole the investigation has shown a high standard of work throughout the hospital. No attempt to shuffle off work and no attempt to mislead was shown during the inquiry. It is suggested that, with a view to preventing such an accident in the future, it be a rule:—

"1. That no Residents give a death certificate in respect of the death of an in-patient without an inspection of the body.

"2. That in the event of a death occurring in a ward, that death shall be entered by a Sister who has inspected the body in the Report Book, and that the final stage of laying-out a body be not commenced until one hour after the entry of the death in the Report Book.

"3. In cases in which it is inadvisable to carry this out it will be notified to the Honorary Officer who has had charge of the case in a report by the Ward Sister, countersigned by the Matron."

The Commissioner: Were these recommendations accepted by the Medical Board?—Yes.

*Q.* And have they been adopted by the House Committee—in other words, has a rule been included in the new by-laws for the Residents, or were they included with modifications?—*A.* As I understand, the present rule is that the Resident shall inspect a body in the ward before it is taken to the mortuary. That is the present rule so far as it affects the Residents, but that also covers nursing procedure.

*Q.* Perhaps Mr. Neden can tell us what the actual rule is. The actual rule as modified by the report of this Committee?

Mr. Neden: With particular regard to a body after death, first of all the body shall not be removed until the patient has been seen.

The Commissioner: But are these not in print?—Yes.

*Q.* Well, let us have them, please. I want to know briefly whether these new rules have been brought to the notice of the Residents.

### WHO WAS RESPONSIBLE?

(To Mr. Gayner): There are one or two points in the report and in the evidence to which I want to call your attention. It appears that as a result of your inquiry, and very much more from what was told us yesterday, that the person responsible for the opinion that the patient was dead was the Day Nurse?—*A.* The Senior Day Nurse.

*Q.* Yes; the Day Sister.—*A.* I do not know what "responsible" means. I think the Senior Day Nurse originates the idea that Collier is dead.

*Q.* The idea so originated is conveyed by the Senior Day Nurse to the Day Sister?—*A.* To the Night Nurse, and, I gather, from the Night Nurse to the Day Sister. Then I gather the Day Sister and the Senior Day Nurse meet. Something is said, and I understood that the Senior Day Nurse asked if she should give a hot pack, but I understood that that impression was not received by the Day Sister. The impression received by the Day Sister was should the Senior Day Nurse proceed with the laying-out of the body? That is the impression I gathered from the evidence of all of them. That is the impression made on my mind.

*Q.* But you see we have had in evidence in the Day Sister's own writing that she inscribes this ticket to the effect that he died at 7.30?—*A.* Yes; there is no doubt that Senior Day Nurse, Night Nurse, and Night Sister, and Nurse Seale all knew the man was alive at 7.50. They none of them wished to give any other impression than that he was alive, but that impression was not received——

*Q.* By whom?—*A.* By the Day Sister.

*Q.* The impression was received that he had been dead some time and she wrote it down.—*A.* Yes, most improperly. I think it is a case of dulness and inertia.

*Q.* Then to turn from that there is a note on your notes of evidence that Sister Hutchins, the Night Sister, had actually been spoken to by Collier at 7.40.—*A.* Yes.

*Q.* That I do not think came out in evidence yesterday.—*A.* Our inquiry yesterday did not take cognizance prior to 8.45.

*Q.* Oh, no, no; we began after 7.50.—*A.* I beg your pardon; I meant 7.45, but the question of when Night Sister last saw Collier was not raised yesterday.

*Q.* Certainly not. I knew nothing of it until I read it in these notes, but it comes out actually that Collier spoke to her at 7.40. At 7.50 Nurse noticed he became cyanosed and showed signs of convulsions, but it makes it all the more remarkable that his death should be assumed.—*A.* In spite of the fact that four people perfectly well knew he had been alive.

*Q.* I think we ought to see Sister Hutchins as to whether that was so. The note is: "Sister H. told Sister C. that Collier had spoken to her at 7.40, and that Dr. S. had ordered a hot pack." I want to call Nurse Hutchins on that. There is yet another point. "The following evening at 8 o'clock Sister learnt what had occurred from Nurse Hunt, but failed to believe it." Which Sister is that?—*A.* That would be the Night Sister. She had been off during the day.

*Q.* It is obscure, rather.—*A.* She would not accept it.

### WHAT SISTER FAILED TO BELIEVE.

*Q.* She failed to believe what?—*A.* She failed to believe that the man had died.

Dr. Macdonald : She could hardly realise it.

*Q.* The statement is this: "The following evening at 8 o'clock Sister learnt what had occurred from Nurse Hunt, but failed to believe it." We had better ask her exactly what that phrase meant. Then, Mr. Gayner, you were good enough to tell us yesterday that there was an excellent practice in the hospital that these reports of the Night Nurse to the Day Sister were written, and I see this amongst your rough notes: "Sister H. reports" (Sister Hutchins that must be) "that the last three lines in the night report *re* Collier September 21 she believes were inserted on September 23, these lines being marked off from the night report proper." Passing from that, has the Medical Board any definite constitution or functions under the rule of the hospital? So far as I have got at present from Mr. Neden it has the appointment of residents, the medical staff sitting in conjunction with the House Committee. I am asking you because I have not had time to study all these questions. In fact, I have not had the literature supplied to me whether the Medical Board has any duties under the constitution.

### SOME NEW RULES.

Mr. Procter : I think you have had a copy of the rules handed to you.

The Commissioner : I have had them handed to me just this moment.

Mr. Procter : Rule 47 is the one.

The Commissioner (after reading rule) : Then the Medical Board is not an executive body. It is a consultative body.

Mr. Procter : A consultative body except that they appoint two members to the House Committee.

The Commissioner : Yes.

Mr. Procter : And they also appoint on the removal of them.

The Commissioner : We have already heard that they took no part in the removal of the officers. That we heard from Mr. Neden yesterday.

The Commissioner (to Mr. Baker) : I think you ought to have the opportunity of asking Mr. Gayner any questions.

### DEATH CERTIFICATES.

Mr. Baker : On the death certificate first of all. I understand you made a recommendation that death certificates were never to be given by the Residents——

The Commissioner : Pardon me; I ought to read what has actually been done. The rule now stands, No. 25; it is not in print, but it has been altered to read : "The Resident Officer shall not give a death certificate in respect of the body of any patient without an inspection of the body before its removal from the ward, and such removal shall not take place without an interval—until an interval of two hours has elapsed since the death occurred."

Those are the steps taken by the House Committee to give effect to the recommendation.

Mr. Baker : When you made that recommendation, Mr. Gayner, was it in your mind at all that either of these two gentlemen whom I represent had been in the habit of signing death certificates without seeing the body? Was the imputation against them?—*A.* Not in the least.

*Q.* Not in the least?—*A.* No.

*Q.* As to the laying-out one hour after death in the report, were you thinking of any other cases except Collier's case when you made that recommendation?—*A.* No.

The Legal Assessor : There is an allegation, No. 15, in The Hospital. Is that question directed to that?

Mr. Baker : I am not going to refer to any other cases, but I am suggesting that this recommendation of the Board of the new rule——

The Legal Assessor : Bears out the suggestion here that that was a proper recommendation to make?

Mr. Baker : Yes, as regards there being one case, but No. 15, "It seemed to be customary to lay out bodies and remove them to the mortuary as soon as possible," can go.

Mr. Gayner : One case has not happened with our staff. It was an outside sister in the case which occurred.

The Legal Assessor : It looked as if there were several other cases.

Mr. Baker : I quite accept that there was no other case. I only wish to point out that the recommendation is coincident with the suggestion by Drs. Shepherd and Macqueen in their letter.

Mr. Gayner : I do not know that the recommendation implies any change in custom, but it is putting in writing what has been a matter of oral tradition.

*Q.* Of course, that does not affect my point, which is that if it was worth while to renew the rule, then at any rate there were grounds for suggesting that it had not been observed.

The Legal Assessor : With regard to Collier?

Mr. Gayner : With regard to No. 15.

The Legal Assessor : Yes, but I should like to ask a question that was not before you when you made the investigation.

Mr. Procter : Had this particular allegation been made by Drs. Shepherd and Macqueen when you made your investigation?

Mr. Gayner : No. In my investigation I tried to find out what was the custom in the absence of a written rule.

### WAS COLLIER CONSCIOUS?

The Commissioner : You were in charge of this poor fellow Collier?—Yes.

*Q.* I think it will probably be to the satisfaction of the public, at any rate, if we could hear from you as to his condition, and whether it was at all likely that he was conscious that he had been removed to the mortuary?—*A.* I think it is not in the least likely.

*Q.* Mr. Procter's examination yesterday was somewhat directed to the point that after all he was dead.

Mr. Procter : That he might be dead. I did not wish to go further than that.

The Commissioner : Have you formed any opinion on the evidence which has been given you by Drs. Shepherd and Macqueen?

Mr. Gayner : At my inquiry I did not investigate that point. I occupied myself with what happened in the ward and with the custom of the hospital with regard to laying out bodies. I, as you will see from the notes, investigated the subsequent fate of the patient very briefly.

The Commissioner: Another point I should like to ask you is this: In making your investigation did you consider at all this allegation which is now strongly supported—namely, that the Residents actually left Collier in the mortuary and had their luncheon before ultimately they removed him to the Isolation Ward. Did that come before you?—It did not come before me at all. Unfortunately I did not call the sister.

*Q.* It is not a question upon which you could give us any more information than the evidence yesterday?—*A.* No.

Mr. Baker: Sister Guy corroborated the story of my clients.

The Commissioner: She said that she saw one or two ounces of blood on the floor.

### SISTER HUTCHINS RECALLED.

The Commissioner: There are one or two points I want to ask you about with regard to Collier. In the rough notes of Mr. Gayner's inquiry there stands the statement that Sister Hutchins told Sister Chapman that Collier had spoken to her at 7.40. Was that so?

Sister Hutchins: Yes.

*Q.* Then did you leave the ward shortly after that?—*A.* I was walking out of the ward.

The Commissioner: Shortly after that they were sending for you and you were not immediately available?—No.

*Q.* And I understood you to say that you did not yourself investigate whether Collier was dead?—*A.* No, Sir, I did not.

*Q.* Then there is this rather obscure note: "The following evening at eight o'clock Sister learnt what had occurred from Nurse Hunt, but failed to believe it." What is the meaning of that?—*A.* I was so astonished; we could not understand it.

*Q.* Astonished that he was dead or alive?—*A.* That he was alive. It seemed an impossible thing to me that the man could be taken down to the mortuary and be alive.

*Q.* Then on this in nurse's report there is a note there that says that "Sister H. reports that the last three lines in the night report *re* Collier, September 21, she believes were inserted on September 23, these lines being marked off from the night report proper"?—*A.* Well, I saw this put in.

*Q.* On the 23rd?—*A.* Yes, Sir, on the Monday morning.

*Q.* Then the note reads rather obscurely in view of the facts. "Nurse H. reports that these three lines she believes were inserted on September 23." As a matter of fact, you saw them actually written?—*A.* Yes.

*Q.* Did you tell Mr. Gayner that you actually saw those lines written yourself on the 23rd?—*A.* I cannot remember now.

*Q.* Were you present, Mr. Neden, yesterday?

Mr. Neden: I think myself Miss Tute's evidence was about the addition of these lines. I am inclined to believe so; I am speaking from memory. I know she had something to do with those lines.

The Commissioner: Perhaps you can find it. It is curiously phrased, if the sister actually saw the lines written. The entry is unimportant; it represents what was given in evidence by the nurse. Will you read the Matron's remarks, Mr. Neden?

Mr. Neden: Miss Tute reports that on the 23rd, considering the night report to be imperfect, she instructed Nurse Hunt to fill in history of case of Collier up to 8 A.M. Miss Tute drew a line across the page marking off the portion added on the 23rd. That is shown in the book.

*Q.* Who was examined first, sister or Miss Tute?—*A.* Sister.

*Q.* So the note was put in that guarded manner, although, as a matter of fact, the sister had seen these lines written on the 23rd?

Mr. Procter: That closes the Collier case.

### TREATMENT OF CASE OF SURGICAL SHOCK.

Mr. Procter: May I ask you to interpose the evidence of Dr. Charles, who is here from Ipswich, and especially wishes to return by the train leaving at 4 o'clock?

The Commissioner: Who is Dr. Charles?

Mr. Procter: He was a former Resident, and he can speak to the case of Thomas, page 647, No. 4.

Mr. Baker: Would you like, before we leave Collier's case, to hear Dr. Macqueen? I was proposing, if they wanted to put any questions to him on this case, to put questions to him quite generally to give them the opportunity of cross-examination if it is wanted?

Mr. Procter: I have some witnesses.

The Commissioner: We shall deal more fully with Collier's case, I am sure. (To Mr. Procter): It is the treatment of surgical shock?

Mr. Procter: Yes.

The Commissioner: How does Dr. Charles come into it?

Mr. Procter: Because he was the House Surgeon at the time, and had charge of the case.

The Commissioner: They were not cases that had anything to do with Dr. Macqueen?

Mr. Procter: Dr. Macqueen, I believe, was then House Physician and Dr. Charles was House Surgeon.

Mr. Baker: Which case is this?

Mr. Procter: No. 4, the Treatment of Surgical Shock.

The Commissioner: Very well, we will hear Dr. Charles. We are passing for the moment from the case of Collier to the case No. 4 on page 647, Treatment of Surgical Shock.

"A patient who had a prolonged abdominal operation was taken to a cold ward where the fire had been newly lit, where there was only one hot-water bottle in the bed, and was left while under the influence of the anæsthetic upon a trolley, with no one to look after her."

We are taking that case first, or, at any rate, some evidence concerning it, because Dr. Charles, who was then in residence here, has specially come up to give evidence and wants to get away as soon as he can, so that if he is here we will listen to him.

Mr. Procter: I don't know whether Dr. Macqueen wishes to say anything about that in support of it.

The Commissioner: Well, I should have rather liked to have Dr. Charles here.

Mr. Procter: Dr. Charles has made a report to the management which perhaps it will be convenient if I read in his presence.

"It is the case of Frances Thomas, aged 55, admitted to Ward No. 6 Medical on August 22, 1912. On the morning of the 24th August I saw the patient for the first time at the request of Dr. Macqueen, who was then House Physician. I advised an immediate operation, and consulted Mr. Jalland with that object. He agreed with my diagnosis, and an extensive operation was performed by Mr. Jalland, at which I assisted. The operation was resection of gut, 14 inches removed. The patient was very ill on the operating table, and stimulants were given. Before she left the theatre I gave orders that a bed was to be prepared with hot blankets, hot-water bottles, etc., in Ward No. 1. I walked down the stairs and met the patient as she was being brought out of the lift and accompanied her to the ward. As the patient was in a most critical condition, I had her placed in front of the fire, screened around, and I personally ascertained that she was properly provided with all that was necessary in the way of hot blankets, water bottles, etc., and I gave her a hypodermic injection of oil of camphor and pituitary extract. I visited her several times within an hour, and by my orders a "special" was in constant attendance, and the patient was not left for an instant until death occurred in the evening. As I was obliged to go out, I requested Dr. Macqueen to look after the patient, and to give intravenous saline, which order was duly carried out. When I returned to the hospital, Dr. Macqueen reported to me that the case was still in a critical condition, time about 6.30. I immediately went to see the patient; she was then practically dying, and death eventually took place about 7.30. The statement published in The Hospital of the 15th March that this patient was left on a trolley unattended is totally untrue, as I accompanied her to the ward, where I remained for at least half an hour. Dr. Macqueen had no personal knowledge of what took place when the patient reached the ward, as he was taking lunch at the time.

"(Signed) R. Charles,
"Late Senior House Surgeon at the County Hospital, York.
"7.4.13."

"There were two fires in the ward the whole of the previous day, and on the morning of the patient's removal to the ward there was a fire from 7 a.m. until the patient was received at 1.30 p.m.

### DR. CHARLES' EVIDENCE.

Mr. Procter: Do you confirm that statement, Dr. Charles?—That is correct according to my knowledge, which I think is almost accurate in detail. It is some time since the patient was operated on, and one cannot be perfectly accurate as regards everything. As in many cases emergency operations occur when one leaves the theatre and goes from the theatre with the patient to the ward, you are liable at times to mix things up a little bit, but to my best knowledge conscientiously, I thing that that is a true statement. One other thing which I have been thinking over since, and that is whether Dr. Macqueen accompanied me to the ward or not. He was at lunch, I know, part of the time. As regards whether he came to the ward immediately the patient was admitted, I am not prepared to say definitely, but I think my best belief is that he did not accompany me at that time, but that he was still at lunch.

The Commissioner: Dr. Macqueen, will you give us your account? I suppose you have only seen this just at this moment.

Dr. Charles: This is, to my best knowledge, some time after the occurrence, you see.

Dr. Macqueen: Mr. Jalland was never present at the operation, as far as I can remember.

The Commissioner: Do you remember who did perform the operation?

Dr. Macqueen: Mr. Charles performed the operation himself. The patient was under my care before she was taken to the theatre.

The Commissioner asked what the case was, and Dr. Charles mentioned the complaint and said the symptoms were intestinal obstruction.

### WHO PERFORMED THE OPERATION?

The Commissioner: And the operation was done by yourself?

Dr. Charles: No; I am afraid Dr. Macqueen is wrong in that statement. That I am perfectly sure of.

*Q.* Your statement is that Mr. Jalland operated?—*A.* Yes; that I am almost sure.

The Commissioner: Then we can have that from Mr. Jalland.

Mr. Baker: Is this a very peculiar complaint?

The Commissioner: We can have that after. The first point of complaint is, who performed the operation. (To Dr. Macqueen): You say that Dr. Charles did it?

Dr. Macqueen: Yes; Mr. Jalland didn't see it.

The Commissioner: Have you any notes of this case?

Dr. Charles: No.

The Commissioner: There must have been notes in a case of this seriousness.

Dr. Charles: It was an emergency case.

Mr. Baker: The next thing is about going there. Dr. Charles, I think, said you were at lunch. What is your recollection of going into the ward?

Dr. Macqueen: The fact was that on that day they were starting to clean the ward to which this patient would have gone—namely, Ward 7—and twenty minutes before the end of the operation a message was sent to the ward asking for a bed to be ready for the patient.

The Commissioner: Ward 7?—7.

Dr. Charles: No; Ward 1.

Dr. Macqueen: 7.

The Commisssioner: 7; yes.

Dr. Macqueen: About a quarter of an hour later a message came back from Ward 7 asking if we would have the bed prepared in Ward 1, as the patients were going to that ward that day.

The Commissioner: Because the other ward was being cleaned out?—Yes; the ward was Ward 1. A message was sent to get a bed ready in Ward 1.

Mr. Baker: Did you hear those orders given?—I did. This was just about five minutes prior to the end of the operation.

The Commissioner: I have not quite understood from your account whether Ward 1 was a ward that was in occupation?—Ward 1 was empty at the time. There was no patient at all.

The Commissioner: Is it a single-bedded ward or more beds than one?—There are more than one.

*Q.* Was the ward occupied by other patients?—*A.* There were no other patients. They were going that afternoon from Ward 7.

*Q.* This is leading up to the question whether there was a fire in the ward?—*A.* Yes.

*Q.* And your impression is?—*A.* That there was one fire which had been newly lit.

*Q.* This being the first case which entered the ward?—*A.* The first case which entered the ward.

*Q.* Then there is a discrepancy of evidence as to the fire. There is also the question as to the operator.—*A.* There were only two hot-water bottles in the bed.

Mr. Procter: Yes, that is true. There are two hot-water bottles now.

The Commissioner: In the statement in The Hospital there was one?—"where there was only one hot-water bottle"?

Dr. Macqueen: Well, it might be one or two.

The Commissioner: Then the other point is the question of this trolley. How long was she left on the trolley?—The patient naturally was coming out of the anæsthetic after the operation. I believe, by Dr. Charles' orders, she was left upon the trolley in front of the fire till the bed had been properly aired, and when I went into the ward there was no nurse attending the patient. That is the point which I wish to bring out. There was no nurse in the ward when I entered it, and the patient was there coming out of the anæsthetic on the trolley. Then, as regards the afternoon, Dr. Charles had to go out in the afternoon, and I gave an intravenous saline, but the patient was dead before Dr. Charles returned.

The Commissioner: Is that so, Dr. Charles?

Dr. Charles: Well, as I say, as regards the accuracy of the latter part of it, to my best belief, I have got the impression of walking to the ward and seeing the patient just a few minutes before her death. That is my recollection of the whole case. I think the whole thing can be very reasonably explained from top to bottom. I mean this: The patient was first in the Medical Ward. That is to say, when a patient is first in the Medical Ward, and a surgical operation has to be performed, then as soon as the operation is performed the patient is transferred to the Surgical Ward. The operation was performed, and she was immediately transferred to the Surgical Ward. The Surgical Wards are in the rooms No. 7 and No. 1. 7 was going under spring-cleaning, and the beds meantime during the operation in the morning had been transferred from 7 to 1. 7 was warm, and I personally can swear to a fire being in Ward 1 the day before. I had been in Ward 1 the day before. I can only say that the fire in Ward 1—well, I would not say it was newly lit; it had been lit for some time, but how long I would not like to say. My reason for transferring the patient from 7 to 1 was that the serious condition of the patient necessitated it. In other words, left in No. 7 when all the nurses were going to No. 1, she would be practically alone. I thought then it would be best, as the condition was so critical, that she should be transferred to Ward 1, where the surgical nurses were. The patient was put into the trolley on the lift, and I personally did not go down the lift myself; I walked down the stairs.

I met the patient at the lift here with Sister and Nurse; I then walked into the ward with the patient. The whole dispute as regards this case, as regards whether the patient was on the trolley or on the bed, is that we used a low trolley in the theatre. It was covered with blankets and was very like a bed. I made the mistake myself.

Mr. Baker: I do not think there is any dispute as to being on a bed or trolley. I think we are at one as to that. She was on the trolley?

Dr. Charles: She was coming from the trolley.

Mr. Baker: On the trolley?

Dr. Charles: Yes, but death did not take place on the trolley. It took place on the bed.

The Commissioner: How do you know that there was a "special" in constant attendance?—I asked the Matron in the dining-room to put a "special" on.

*Q.* I see your next remark is: "As I was obliged to go out, I requested Dr. Macqueen to look after the patient."—*A.* Yes.

Mr. Baker: What time was that?—About 3 o'clock. I should say between half-past two and three.

The Commissioner: Your statement that the patient was not left for an instant until death occurred is a thing which was not entirely within your knowledge?—Yes; but I asked for a "special" to be put on, and I am taking the goodwill of the Matron.

*Q.* You asked for a "special"?—*A.* And during my presence she was put on. I was standing in the dining-room at the time, and I sent a special note to the Matron to order a "special," and she came out from her lunch and ordered a "special."

*Q.* Did you actually see the "special" come into the ward to take charge of the patient?—*A.* The "special" was in the ward when I went to visit the patient previous to going out.

Dr. Macqueen: My point was that prior to that the patient was coming out of the anæsthetic shortly after the operation.

Mr. Baker: This was on August 24.

Dr. Charles: The date I cannot remember.

*Q.* Well, I have taken that from your statement. You begin by saying, "On the morning of the 24th August."—*A.* Yes, according to the book.

*Q.* Well, when did you prepare this report?—*A.* About a week ago. More; it is a fortnight ago.

*Q.* From the book, you say?—*A.* No one was present at all. I scribbled it out.

*Q.* According to your recollection?—*A.* According to my recollection, which, I think, is truthful.

*Q.* I do not doubt it being truthful so far as you can remember. I want to ask you what quite your recollection is in this case. Do you think, for one thing, that there can be any doubt we are dealing with the same patient?—*A.* Not a shred of doubt; that I am positive. Resections do not take place every day. This was an unusual case.

*Q.* How many do you suppose you dealt with during August—operations of this kind?—*A.* There were four of these resections during my period.

*Q.* How many were there in August?—*A.* That I could not say.

*Q.* Were there more than one?—*A.* I really could not say.

*Q.* Might there be more than one?—*A.* There might have been for aught I know. I know that there was an intestinary section—that of a man called Smith.

*Q.* Was that before or after?—*A.* That was before.

*Q.* Do you remember any case after this—in September?—*A.* Yes. An old man; performed by Mr. Goddard, I think.

*Q.* Is that the only case in September?—*A.* Really, I could not say.

The Commissioner: It would be very difficult.

Mr. Baker: I have no doubt it would be difficult, and that is rather my point.

Dr. Charles: I can assure you that I have not the slightest shadow of doubt about this case, because it is a case I took a great interest in. We discussed fully as to whether the case would be a strangulated hernia, internal hernia, or a kink in the bowel. I am rather pleased that we did find what was expected. We thought it might have been a growth, but it was acute strangulation.

### CONFLICTING STATEMENTS.

Mr. Baker: You know I mean that there is direct conflict of evidence between you and Dr. Macqueen about several incidents?—As regards this?

*Q.* Yes.—*A.* Well, I think it can all be reasonably explained.

*Q.* You are not suggesting that Dr. Macqueen is not speaking the truth?—*A.* No; from what I know of Dr. Macqueen, I can say this, that I think conscientiously what he has stated he believes to be true. I say the same of myself.

*Q.* And then you say you think you could explain?—*A.* When the patient was transferred from Ward 7 to Ward 1, and when——

*Q.* But you know it is more than that. The first direct conflict is as to Mr. Jalland—that Mr. Jalland did not see the case?—*A.* Oh! I am positive about that; that I can swear to.

*Q.* Your name is down in the book?—*A.* My name is recorded in the book as doing the operation. That is not true. That was a mistake on the part of the Sister which I asked her to correct. Dr. Evelyn was present, and was also at the operation.

*Q.* Then the second point, about the fire. Dr. Macqueen says he is quite clear that the fire in Ward 1 had been newly lit. I thought perhaps you would explain the discrepancy here, because I understood you to say that there was a fire the day before?—*A.* Yes; I was in the ward the day before.

*Q.* And you know that this one fire in Ward 1 had been lighted some little time before?—*A.* Well, no one informed me so, but I go by the look of the fire. When I went to the patient I thought the ward was not warm enough, and I immediately put the patient quite close to the fire. The bed was pulled along the fire, screens were brought there, and every available nurse was engaged filling hot-water bottles; and this is probably where the mistake is, where Dr. Macqueen has found the absence.

*Q.* Do you mean that they were engaged filling hot-water bottles outside the ward?—*A.* In the ward scullery.

*Q.* Would that explain Dr. Macqueen's statement that the patient was left on the trolley without anybody in attendance?—*A.* No; I was there myself at that time, because I personally superintended these hot-water bottles being put into the bed. I myself put two.

*Q.* And do you mean to say that at no period at all was the patient left unattended?—*A.* Not to my recollection.

*Q.* Do you think your recollection may be at fault?—*A.* I do not think so. Of course, as I say, it is a long time back, but I am personally responsible for the case and for the after-treatment. I think it would be a grave charge against me to say that I had no one in the ward, and left a patient on a trolley.

*Q.* I am not so familiar with these cases as you or Dr. Macqueen, but I suppose it is impossible for a doctor to be in attendance all the time?—*A.* Oh, yes.

*Q.* Would it necessarily have been a breach of your duty if you had left a patient?—*A.* Decidedly; if I put that patient into a cold ward I should look upon it as one of a serious kind.

*Q.* And you would not think it sufficient to leave her in charge of an experienced nurse?—*A.* I would. I gave her my directions.

*Q.* You would think that was sufficient?—*A.* Yes, but I would not think it sufficient to leave a patient in the ward alone.

*Q.* I understood you first to say you must be there yourself.—*A.* I must be there myself when I am giving directions as regards the treatment of the patient.

*Q.* But you told me that you were there the whole time?—*A.* Oh, that would be utterly impossible.

*Q.* Of course, I am speaking of the period which immediately followed her removal into the ward.—*A.* That I can almost swear to. That in fact, I will swear to.

*Q.* When did you first bring her into the ward?—*A.* I believe at 25 past 1, or between 1 and half-past. That is to my best knowledge. I did not consider two hot-water bottles quite sufficient, and I passed the remark to Dr. Macqueen.

*Q.* And what orders did you give?—*A.* I ordered four more bottles to be given. And the reason that there were only two——

*Q.* Did you give any orders about bottles before?—*A.* I did give orders for hot-water bottles to be put in the bed. In ordinary cases there are only two. The reason there was not more was that the Medical Sister and the Nurse usually accompany patients to the theatre and then cease to have anything to do with them. A Surgical Nursing Sister then takes the patient on. If the Surgical Nursing Sister had been in the theatre at the time, I feel certain, judging by the Sister of the Hospital, there would have been at least six hot-water bottles, and the patient would not have required to have been brought to the fire. It was owing to that she was brought to the fire and very quickly warmed.

*Q.* Did you make any complaint when you found there were only two bottles?—*A.* Yes.

*Q.* To whom?—*A.* To the Matron. I sent to her to get a "special," and to see about getting hot-water bottles.

The Commissioner: Did you mention in the note to the Matron the question of hot-water bottles?—No; that I personally superintended myself. That only meant a matter of a few minutes.

The Commissioner: I see that the entry in the operation register is "No. 410. Case 24. Thomas, Frances. 55. Married. VII."—unless that is intended to be I.

Dr. Charles (looking at the book): That is my writing.

The Commissioner: The operation was for intestinal obstruction caused by Meckle's diverticulum, and the surgeon is put down as Mr. Charles.

Dr. Charles: Well, that is wrong. That is the sister's writing. That is a mistake entirely. It is evidently confused with the case below.

Mr. Baker: You know that Dr. Macqueen says that the operation was performed by you, and there is the entry partly in your handwriting, and partly in the handwriting of the nurse?—Yes, and if I had performed it I would have put my name down.

*Q.* From which it appears that you performed the operation?—*A.* No, sir; that is not my writing of my name.

*Q.* Well, but that is what the entry says. What I would ask you on that is this: Do you back your recollection now against this entry and against the positive statement of Dr. Macqueen?—*A.* I personally called the attention of the Sister to that mistake.

*Q.* When did you do that?—*A.* About two days after.

*Q.* To which Sister?—Sister Sly.

*Q.* And can you explain why it was not altered?—*A.* I could not say.

*Q.* Is Sister Sly in the hospital now?—*A.* That I could not say.

### WHY DR. CHARLES GIVES EVIDENCE.

*Q.* How do you come to be here?—*A.* Mr. Neden wrote me, but previously to that I came personally to ask Mr. Neden, and I told him that as regards this patient, Thomas, I personally was concerned in that, and that I personally would like to come here because I looked upon it as a reflection upon myself, and that is why I am here, and that is why I know the inquiry is on at all.

Mr. Baker: Do you remember an occasion when you had a conversation with Dr. Shepherd at Leeds?—Yes.

*Q.* Did you say to him then that the nurse was not in attendance on the trolley?—*A.* No; I say I dispute that entirely, the incident as regards the trolley.

*Q.* Do you deny stating to him that the nurse was not in attendance?—*A.* Yes. I remember distinctly, and I say that in my last letter to Dr. Macqueen—that as regards the report very little concerns myself except this case, and in that, I said, I cannot agree with you. Dr. Macqueen has been kind enough to produce this letter. Let him produce this last one.

*Q.* I am speaking of Dr. Shepherd.—*A.* Dr. Macqueen was present. I don't think, in fact I am sure that, I did not. I said: "As regards the report, there is one thing on which I can't agree with you."

Dr. Shepherd was then examined by Mr. Baker with regard to this incident.

Mr. Baker: Do you remember an occasion in Leeds when you, in the presence of Dr. Macqueen, I understand, saw Dr. Charles?

Dr. Shepherd: Yes.

*Q.* When was that?—*A.* One Saturday night; about a month ago, I should say.

*Q.* Did Dr. Charles then say anything to you about this trolley incident?—*A.* He did. Dr. Macqueen and he were discussing it. He said he disagreed with the fact as to the moving of the patient to the ward, but he emphatically stated that there was nobody with the case at the time when he got into the ward. I can only say what I heard.

Dr. Charles: I am sorry to disagree, but that is my opinion.

Mr. Baker: I would ask the same question of Dr. Macqueen. Do you remember that incident at Leeds on a Saturday. Have you heard what Dr. Shepherd said?—Yes.

*Q.* Is it true?—*A.* Yes.

Dr. Charles: I remember distinctly well taking the report. As a matter of fact I had not even read all the report at that time, I was so busy with my own affairs. I said, "I believe there is one case about a trolley, a case that I am concerned in." I said all the other allegations made against the hospital were not concerning me, but I said "That is the one I disagree with." That is to my best recollection. And I said that in a letter to Dr. Macqueen.

Dr. Macqueen (producing another letter): Would Dr. Charles like this letter produced? It is marked "private"?

Dr. Charles: Yes, I don't mind a bit. Bring everything forward that can possibly throw light on anything.

Dr. Macqueen passed the letter to Dr. Charles, who read it. The gist of it was as follows: "I have just found time to write to you a few lines. I have carefully read The Hospital report, and as I said to you at Leeds I cannot support you in all the allegations. Most of them occurred during the house surgeoncy of ——. I agree with you as to the inadequacy of the house staff, also the time children are in bed, which I know was quickly put a stop to. In the other allegation which concerns myself—that is the trolley incident—you are quite wrong as regards the statement. Everything was done that could be done, so that I cannot agree with you; while you yourself were having lunch at the time. It is true there were only two hot-water bottles in the bed."

Mr. Baker: I think you will agree with me it was fair to Dr. Macqueen to read that letter.

The Commissioner: How long was there between the interview at Leeds and the writing of that letter?

Mr. Baker: This letter has only just been received. It is dated April 15.

The Commissioner: And when did this interview take place at Leeds?

Dr. Charles: Oh, about fourteen days ago.

Mr. Watson: Where did the interview take place?—In the hotel, in the lounge at the Queen's Hotel.

*Q.* Was it an interview over a lunch, or a serious discussion of the case?—*A.* Oh, a serious discussion.

Mr. Baker: This last letter was written after Dr. Charles knew that this inquiry was coming on.

The Commissioner: That is why I wanted to know what time elapsed between this interview and the letter.

Dr. Charles: About a fortnight.

Mr. Baker: And in between the Leeds incident and the writing of this letter you had had a letter——?—I said to Dr. Macqueen at Leeds, "I have not carefully read The Hospital report. After I have read it," I said, "I shall write to you."

*Q.* Yes. When did you get Mr. Neden's letter? On Thursday morning last?—*A.* Yes. And I said to Dr. Macqueen, "I have not carefully read The Hospital report. There is one thing I have read in which I cannot agree with you, and that is the trolley case." I got a copy of The Hospital report and read it. I then communicated with the Secretary about this case, which I thought personally concerned myself, and as soon as I had time I wrote Dr. Macqueen.

Mr. Procter : I should like to ask Dr. Macqueen how this interview at Leeds came about.

Dr. Macqueen : Well, I happened to hear that Dr. Charles was in Leeds, and called on him, as I had to be there the same day.

*Q.* Had you asked him to come to Leeds, or how was it?—*A.* No, I had not asked him to come.

*Q.* You heard accidentally that he was in Leeds?—*A.* Yes.

*Q.* And you went to him?—*A.* Yes.

*Q.* And what was the object of going to him?—*A.* Naturally, he having been a Resident here, I went to him to discuss the affair.

*Q.* To get information to support you, I suppose?—*A.* To get his views on all the matters.

*Q.* It was purely an accidental circumstance that he and you were in Leeds together?—*A.* Yes.

### DR. MACQUEEN EXAMINED.

*Q.* I should just like to ask you, Dr. Macqueen, how long you say the patient was left on the trolley without attendance. Do you know?—*A.* I don't know how long the patient was left.

*Q.* How long did you observe the patient without attendance?—*A.* For a few minutes.

*Q.* Did you wait and see if anybody came?—*A.* Yes, somebody did come.

*Q.* Who came?—*A.* I cannot remember now.

*Q.* Was it a nurse?—*A.* Yes, a nurse.

*Q.* How long were you there when she was unattended?—*A.* A few minutes.

*Q.* Did the nurse give you any explanation as to why she had been left?—*A.* No.

*Q.* Did you ask for any explanation?—*A.* No; the case was not under my charge.

*Q.* I thought you were in charge for Dr. Charles for the moment?—*A.* No; this was before Dr. Charles left the hospital.

*Q.* When you say a few minutes, how many? Ten minutes?—*A.* About five minutes.

*Q.* Was any inquiry made as to how it came about?—*A.* I told Mr. Charles about it.

*Q.* But not more than five minutes?—*A.* Not more than five minutes. But the patient was coming out of an anæsthetic, and might quite easily have fallen off the trolley in front of the fire. That was the point I wished to make.

*Q.* Was the patient on the trolley?—*A.* On the trolley in front of the fire at the time.

Dr. Charles : That is not correct, because I personally myself helped to take the patient from the trolley.

Dr. Charles added that before going he was quite willing to give every information that was possible.

The Commissioner : I think that is all.

Dr. Charles then left the witness-chair.

### MR. JALLAND CALLED.

Mr. Jalland said : Might I first say with regard to that book ("The Register of Operations"), when I saw in The Hospital about this inquiry and that particular case, I went up to find out which case was referred to. I went up to the theatre—some weeks ago now—and I referred to this case. I said, "But they have put Dr. Charles down as operating. It was not his case. I operated upon that." The Sister of the ward said, "I was not in charge at the time. There was a stranger here, and I suppose she saw Dr. Charles helping, and she put it down. That is how it occurred." I only wish to say that just by way of correction.

The Commissioner : Then you entirely agree with Dr. Charles that you were the operator?

Mr. Jalland : I was the operator. I had better explain it. He was assisting me at the operation. I made my preliminary incision, opened the abdomen, and found the strangulation. When you are going to remove a portion of gut you require two sets of hands. We clamped the gut up, and then Dr. Charles removed the portion, and we put a tube in.

The Commissioner : Do you agree with that, Dr. Macqueen?

Dr. Macqueen : No, Sir; Mr. Jalland was not even in the theatre.

Mr. Jalland : Well, I must say that is distinctly untrue. I was there; I operated, opened the abdomen.

Mr. Procter : Is it a fact that Dr. Evelyn was there at the time?

Mr. Jalland : Yes; he came in when we were in the middle of it.

Mr. Baker : When did you first call attention to the entry being wrong, Mr. Jalland?

Mr. Jalland : A few weeks ago, after I saw this in The Hospital, in the paper. I was looking over to see what these cases were that were referred to.

*Q.* After the end of January?—*A.* I cannot remember the date.

*Q.* Can you explain how it was that the entry was not put right?—*A.* I don't know at all. I pointed it out to the Sister. The Sister of the theatre had not charge of the theatre at the time this operation was done. It was not a matter of any moment at all, and she would never trouble about it. But I pointed out to her the mistake that had occurred.

*Q.* You did not suggest it should be altered?—*A.* No, there was no occasion for it. There was no necessity for it.

The Commissioner : I don't think there is any difficulty in understanding how a Sister might have entered the operation either under Mr. Jalland's name or Dr. Charles's name, because according to the evidence the operation was practically a joint operation. But what, of course, I entirely fail to understand is how Dr. Macqueen should have been wrong in saying Mr. Jalland was not there at all. That is impossible to understand. As to that point we shall have other evidence.

Mr. Watson : I suppose, Mr. Jalland, it is very unlikely you can possibly have made a mistake, and muddled the operations in your mind?

Mr. Jalland : Oh, no. It is not a common thing to make operations of this kind. It is a rare thing to get strangulation.

### DR. EVELYN'S STATEMENT.

The Commissioner : This seems an extremely simple question, whether Mr. Jalland was present in the theatre at a certain operation, or whether he was not.

Dr. Evelyn : Well, I am very sorry I cannot focus the question at all at present. The case presumably was under my charge as a medical case, to begin with, and then was transferred to the surgical side, and one does not follow all the cases up from the medical side to the surgical side. I cannot at the present moment honestly say that I remember seeing anything of the operation at all.

The Commissioner : Well, thank you very much; I don't think we have any more to ask you.

### NURSE MOULS CALLED.

The Commissioner : You are summoned to tell us about the operation which took place upon this Frances Thomas. There is a direct conflict of testimony as to who was present at the operation. Mr. Jalland and Dr. Charles say that they were both present, and both were responsible for the operation. Dr. Macqueen says that Mr. Jalland was not in the theatre at all.

Nurse Mouls : Mr. Jalland was present. They waited for Mr. Jalland.

Mr. Baker : Who gave the anæsthetic?—Dr. Macqueen.

*Q.* You are quite certain that Mr. Jalland was there?—*A.* Yes, quite certain.

*Q.* What part did he take in the operation?—*A.* He started operating, and Dr. Charles was assisting, and then Dr. Charles started operating. They changed sides of the table.

The Commissioner : Then they were both operating together?—Both operating together.

Mr. Baker : Can you tell me which Sister was present ?—Sister Drysdale.

The Commissioner : Is she here ?—Yes.

### SISTER DRYSDALE CALLED.

The Commissioner : We have to ask you about an operation which took place on Frances Thomas, a case of operation in which fourteen inches of intestine were resected. According to one account, Mr. Jalland and Dr. Charles were there, the operation being performed jointly by them, and according to another account Mr. Jalland was not even in the theatre. They cannot both be accurate, and we want to know what you have to tell us about it. Have you any recollection of who was in the theatre at the time that operation was performed ?

Sister Drysdale : Mr. Jalland was in the theatre.

*Q.* What part did he take in the operation, as far as you could see ?—*A.* I could not say exactly.

*Q.* You are clear that Dr. Charles was there ?—*A.* Yes, sir.

*Q.* And who gave the anæsthetic ?—*A.* I don't remember whether Dr. Macqueen gave the anæsthetic or not, sir.

*Q.* You don't know anything as to the case after the patient was taken from the theatre to the ward, I suppose ?—*A.* No, sir.

*Q.* You did not provide any nurse from the theatre ?—*A.* No.

Mr. Baker : Do you remember how many people were present at the operation ?—A nurse, the Ward Sister, and the ward nurse, Dr. Charles, Mr. Jalland, and Dr. Macqueen. I cannot remember whether there were any more.

*Q.* Do you remember how long Mr. Jalland was there ?—*A.* I don't.

### SISTER CHAPMAN CALLED.

Mr. Procter : I don't think she was in the operating theatre.

The Commissioner : What can you tell us about the operation which took place upon Frances Thomas, who was operated on for intestinal obstruction and fourteen inches of intestine were removed ? Can you tell us who operated ?

Sister Chapman : No; I only saw the patient when she came down to the ward. I received her when she came to the ward.

*Q.* Ward No. 1 ?—*A.* Yes.

*Q.* Were you with with the patient all the time after she came into the ward ?—*A.* Only until the "special" was put on, sir.

*Q.* But was the patient left at any time without a nurse ?—*A.* No, sir, never.

*Q.* Either you were there or the "special" was there ?—*A.* Either I was there or the "special" was there.

Mr. Procter : Do you know when she came out of the anæsthetic ?—Yes, sir; about ten minutes after she came down.

*Q.* Were you there at the time ?—*A.* Yes.

*Q.* When she came out ?—*A.* When she came out of the anæsthetic.

The Commissioner : Perhaps you will be able to tell us as to the question whether there were any fires in the ward ?—Yes.

*Q.* Was there any fire in the ward ?—Yes, sir.

*Q.* A good fire ?—*A.* Yes, a good fire.

*Q.* What was the object, then, of putting the patient just in front of the fire ? Was it because the ward was cold, or was there any reason ?—*A.* It was the doctor's orders that she was to be put in front of the fire.

Mr. Baker : The ward was cold, wasn't it ?—I did not consider the ward was cold.

The Commissioner : How many hot-water bottles were there ?—There were two in the bed to begin with, and they were taken out when the trolley came into the ward, for the patient to be laid on the bed. There were four more added, and she was put back into the bed with a hot blanket.

*Q.* Was she put in front of the fire because the supply of hot-water bottles was inadequate to begin with ?—*A.* No, sir, because she received treatment before she was put in front of the fire. She was put in front of the fire about half-an-hour after she came into the ward.

The Commissioner : That does not correspond with your statement, Dr. Charles.

Mr. Procter : You are quite sure that she was not left ?—I am quite sure that she was not left from the time I entered the ward.

Mr. Baker : Do you know the name of the "special" who was attending the case ?—Nurse Seal.

*Q.* I understand you were not present at the operation ?—*A.* No.

*Q.* You received the patient down here ?—*A.* I received the patient in Ward 1.

*Q.* Had you been in Ward 1 before you received her there ?—*A.* Yes; I had been in Ward 1 all the morning.

*Q.* How many patients were there in the ward ?—*A.* At that time there were no patients in the ward.

*Q.* Do you mean when you received her there ?—*A.* When I received her there the patients were not there.

*Q.* Had there been any that morning ?—*A.* It was prepared to receive all my patients from Ward 7, but they were not down then.

The Commissioner : Is that special nurse still in the hospital ?

Mr. Procter : Yes.

Mr. Baker : Do you know who filled the hot-water bottles ?

Sister Chapman : I filled the first one.

*Q.* Was that before the patient came down ?—*A.* That was before the patient came down, and the others were irregular. I put a blanket to warm before the fire, ready for the patient.

*Q.* I understand you to say the ward was not cold ?—*A.* The ward was not cold. No; I had the ward prepared for the reception of the other patients.

*Q.* Do you seriously suggest that the doctor would have ordered the patient to be put on the trolley right in front of the fire if the ward had not been cold ?—*A.* Well, of course, the doctor's opinion may differ from the sister's, but we do not put them near the fire sometimes, especially the children. It is an unusual thing. It is possible that to put the patient before the fire is the best thing to be done.

*Q.* Do you think Dr. Charles thought it was cold ?—*A.* He did not give me his reasons for putting the patient in front of the fire.

### NURSE SEAL CALLED.

The Commissioner : The particular point that I have to put to you is that, as I understand, you were the "special" on this poor woman who was operated on by Mr. Jalland and Mr. Charles, one or both, and what I want to know is, when you went into the ward—Ward No. 1—did you find the patient under the charge of the lady who has just left us, or was she alone ?

Nurse Seal : No, Sir, she was with Sister Chapman.

*Q.* You are quite clear that you took on the patient direct from the Sister who had her in charge when she came from the theatre ?—*A.* Yes, she was there when I came in.

Mr. Baker : Were you there all the time until the patient died ?—Yes.

*Q.* Who was present when the patient died ?—*A.* Sister Chapman and I, and Dr. Macqueen was there at the same time.

*Q.* He was sent for ?—*A.* Yes.

*Q.* Was Dr. Charles present ?—*A.* I don't remember.

*Q.* Did you fill any hot-water bottles ?—*A.* Yes, Sir.

*Q.* Did you put them into the bed ?—*A.* They were already in the bed when I got there. We filled them afterwards, later on in the afternoon.

The Commissioner : How many were in the bed ?—Four, I believe, I am not quite sure.

Mr. Baker : What time in the afternoon was that ?—I could not say. I am not quite sure of the time.

*Q.* It would be several hours after the patient was brought into the ward that you refilled the bottles ?—*A.* Oh, yes.

### THE SECRETARY'S EXPLANATION.

Mr. Procter : I should like to call the Secretary to explain about the changing of the wards. There seems to be some question about the ward being cold, and he says he can explain it.

Mr. Neden : I don't refer to any particular case, but take for example the present case under discussion in Ward 7. It was decided to change from Ward 7 to Ward 1. That would mean to say that Ward 7 would be dismantled for cleaning purposes. Now, then, it had been arranged that the patients from Ward 7 should be transferred to Ward 1, and I believe evidence has already been given that in this particular case there were fires in the whole of the day previous and on the whole of the morning on which this patient was removed. What I want to make clear is this, that questions have been asked were any patients in before. It is obvious when a ward is being changed that there would be no patients in before, because all Ward 7 cases would be reserved for this particular ward that was being changed. There would be no cases in the ward, probably, for one or two days previously, excepting the cases from the ward which would be dismantled ready for cleaning.

The Commissioner : I have no difficulty in understanding that.

### THE BED-SORE AND OTHER CASES.

The Commissioner : The next case is No. 2.

"2. On July 6, 1912, at 12.5 A.M., the Matron reported to the house physician (present house surgeon) about a child in Victoria Ward having a bed-sore. This had been present for twelve days, and had been treated without the knowledge of the Resident. Arrangements had been made for the child to go out that day."

Then there was a letter bearing on that. The passage of the letter, signed by both Residents, on January 11, 1913, is this :

"On July 6, 1912, at 12.5 A.M., the Matron reported to the house physician (the present house surgeon) about a child in Victoria Ward having a bedsore. This had been present for twelve days, and had been treated without the knowledge of the Resident. Arrangements had been made for the child to go out that day."

And this is the addition :

"And evidently this was the only reason why the presence of the bedsore was reported.'

Mr. Baker : That was Dr. Macqueen.

The Commissioner : Do you wish to add anything to that statement, Dr. Macqueen?—No.

The Commissioner : Then I think we had better see the Matron.

The Matron was recalled.

The Commissioner : We are inquiring into the case—I may as well read you the lines :

"On July 6, 1912, at 12.5 A.M., the Matron reported to the house physician (the present house surgeon) about a child in Victoria Ward having a bedsore. This had been present for twelve days, and had been treated without the knowledge of the Resident. Arrangements had been made for the child to go out that day."

Did you know of this bedsore?—I reported a graze; I did not report a bedsore. It is the first mention I have heard of a bedsore.

*Q.* When did you report the graze? The gentleman concerned is Dr. Macqueen?—*A.* At the time he mentions I had left word with sister that I should like to see him, and he came to me when he had finished in the ward.

*Q.* But you had not reported it before then?—*A.* No.

*Q.* It is a fact that it was only reported to him just before the child was going out, and what you reported was a graze and not a bedsore?—*A.* Yes.

*Q.* That does not actually answer the question whether you knew of it before?—*A.* I did know of it. I knew of it the day after it happened. The sister told me of it. It was uncertain how it had happened. It was at first thought it was a burn.

*Q.* These are some notes made of an inquiry held dealing with many questions by the Medical Board. I will read this extract and see whether you confirm it or not. (Reads) :

"Dr. Macqueen reports the Matron reported that she had known of the sore, and it had been present for twelve days. Dr. Macqueen reports that the sore had not been previously reported to him by any one."

That you will agree with?

### SIZE OF THE BED-SORE.

"That the sore was thought to be due to a probationer overheating a blanket and wrapping the child in it after bathing. Dr. Macqueen examined the child the same day and found a sore one inch long and a quarter of an inch broad on the left side of the chest. The sore was superficial and healing. The child was discharged from the hospital, as the ward was being closed for cleaning, and came up for treatment in the Casualty for fourteen days. The sore was not seen by any member of the Honorary staff, nor reported to Dr. Macqueen or the Honorary at any time. The Matron told the house physician that the case had slipped from her memory, or she would have had the case reported sooner. The sore was known to the Matron nine days before Sister Fitton went on holiday."

Then there is a further reference to the case :

"Matron allowed the sore not to be reported. . . ."

Perhaps I should begin the paragraph before :

"Matron believes Sister Victoria reported to her in due course. Matron allowed the sore not to be reported, because she felt that Dr. Macqueen was on bad terms with this sister."

Does that represent the facts, Matron?

*A.* Yes, that is what I stated at the time. It was a very small thing, a very trivial thing, and it was not known how it occurred, and I told sister I would inquire, which I did. I had all the nurses up and inquired very minutely as to what had happened, and the conclusion I came to was what is stated there. It was not a bad sore; it was not a scald or a bottle burn, but I thought a nurse had allowed the blanket to get too hot, and I looked upon it as rather a small matter; it was such a very small case. I believe that it was healed.

*Q.* Almost healed, anyhow?—*A.* I think it was quite healed, and that the child had just fridged with the bandage. If I had seen the nurse——

Mr. Baker : But the child remained under treatment after it left the ward.—That, of course, I do not know about. Dr. Macqueen saw it again.

*Q.* "The child was discharged from hospital as the ward was closed for cleaning, and came up for treatment in the Casualty for fourteen days." It would not have come up for treatment if it had been entirely healed.

Mr. Gayner : My impression is that it did not come up for that bed-sore but for various reasons, of which that might have been one.

The Commissioner : Of course the point of the complaint is not as to the precise measure of the sore or what happened after, but the failure to report to the Resident the fact that this accident had happened, and as to that the facts appear not to be in dispute.

Mr. Gayner : No.

### WAS IT A BED-SORE?

Mr. Procter : Have you any opinion as to whether it was a bed-sore or not?

The Matron : I never had the least doubt about it.

*Q.* Is it your opinion that it was not in fact a bed-sore?—*A.* Certainly.

*Q.* What was it reported to you as being?—*A.* As a sore place and they did not know how it had occurred. That was why I set about to inquire into it and found out what it was and how it had occurred. Had it been a bed-sore or a bottle burn it would have been reported at once.

The Commissioner : May I ask you, Dr. Macqueen, do you admit that the sore was on the left side of the chest?

Dr. Macqueen : Yes.

*Q.* Rather an unusual place for a bed-sore.—*A.* No, the child was a small child, the age was six months, and that child was nearly always wet.

*Q.* Whereabouts on the left side of the chest?—*A.* Here (placing hand low down on body).

Mr. Procter: There are two nurses concerned in this case. I do not know whether you would like to hear them? Of course, as you say, it is as to the reporting.

The Commissioner: I don't want to see them at all unless they ought to have an opportunity of putting the matter before us, but their names are not introduced, and the sore is not the important thing; it is the reporting of the sore; therefore, I think it would be unwise to introduce them unless you have any special reason to do so.

Mr. Baker: The next case, No. 3, has already been dealt with. It is merely another instance of failure to report.

The Commissioner: Yes, we now come to No. 5.

Mr. Procter: Just let me go back to No. 2, the bed-sore. May I ask, Dr. Macqueen, did you report this bed-sore to the Honorary?

Dr. Macqueen: I did not.

*Q.* Is it not the rule that it should be reported?—*A.* It is not customary to report bed-sores to the Honorary.

*Q.* Is it not a rule of the hospital?—*A.* It was not in the rules given to me.

The Legal Assessor: Do I understand him to say that bed-sores should not be reported?

Mr. Baker: He did, and that it was not in the rules given to him.

Mr. Gayner: A breach of discipline.

Mr. Baker: I beg your pardon? What do you say the rule and custom is?

Mr. Gayner: That a bed-sore should be reported.

Mr. Baker: Is it a well-recognised rule and practice in the hospital?

Mr. Gayner: Absolutely. I am not speaking only of ours; it is well accepted at any hospital.

Mr. Baker (to Dr. Macqueen): How long did you know of the sore before the child left the hospital?

Dr. Macqueen: It was reported to me at 12.5 a.m. on the day it was leaving.

Mr. Baker: What opportunity would you have had to report it to the Honorary?—None; the Chairman had left the hospital.

Mr. Gayner: But the case should have been reported to my representative.

The Commissioner: But we have it that the case was not going to the Casualty for the bed-sore alone.

Mr. Procter: Was the child partly attended for the bed-sore?

Dr. Macqueen: Yes.

Mr. Procter: For the whole fortnight?

Dr. Macqueen: Yes.

Mr. Baker: Did you attend it?

Dr. Macqueen: Yes, I was Casualty Officer at the time, and I attended the child.

Mr. Baker: You never reported it at all, treating the child as an out-patient?

Dr. Macqueen: No, I did not.

Mr. Neden: May I just mention, although in that letter which Dr. Macqueen read it was given at 12.5 a.m., I should like his explanation why it was reported at 12.5 a.m. without any record as to the urgency of the case?—Some one might have thought it a very urgent matter indeed, but, as a matter of fact, the Matron had given orders to see Dr. Macqueen at the earliest opportunity, somewhere about 8 o'clock in the evening.

Mr. Baker: I have not made any suggestion.

Mr. Procter: Do you attach any importance to it?

The Commissioner: Not the least.

### CASE NO. 5. DOCTOR'S ORDERS NOT CARRIED OUT.

The Commissioner: Now we come to No. 5: "A patient admitted after an accident (suffering from slight concussion and shock, and who had an exceedingly bad pulse—not in any fit condition to be moved or washed) was found in the process of being washed ten minutes after admission." I have no other details of that case, and therefore I must ask Dr. Shepherd to give details of it.

Dr. Shepherd: It occurred between three and four o'clock.

The Commissioner: Will you tell us the name of the patient and the ward?—I cannot recall the name, it was in Ward 2.

Mr. Procter: Albert Fernley, I believe, aged thirty-four, admitted to Ward No. 2 on December 24, and discharged January 2?—That might be the case, I cannot say definitely; I expect it is.

Mr. Baker: Had you already seen this patient, Dr. Shepherd?

The Commissioner: I think Dr. Shepherd should tell his own story.

Dr. Shepherd: I was on casualty duty in the afternoon, and the patient was brought in on a stretcher; I examined him. His history, so far as I could get it, was that he had been struck by some iron on the head and also about the body and the abdomen, and he was only semi-conscious, and had a very bad shocked condition; and I sent him immediately up to the ward, and ordered that he should be put to bed and left perfectly quiet. My colleague was not there at the time, otherwise it would have been his case. Ten minutes afterwards I was on the way to my own ward; the door of Ward 2 was open, and I noticed that there were screens round one bed, behind which two nurses were working, and I went in and found this patient was in process of being washed.

The Commissioner: I want you to describe it in detail?—He was lying on the top of the usual made-up bed, and one nurse was washing him down while the other was drying him.

The Commissioner: How much of him was uncovered?—About half, fully half.

The Commissioner: The method of washing being that adopted in ordinary cases, not seriously ill?—Yes. I felt his pulse and found it was not at all in good condition; and, having given orders that he should be perfectly quiet, as he was only recovering from shock, I asked why this was not done, and, getting no satisfactory explanation, reported it to Dr. Macqueen.

The Commissioner: Will you tell us the subsequent career of the case?—I did not pay very much attention to it.

*Q.* You can tell me what happened, roughly; what did the case turn out to be; he was a man struck on the head?—*A.* Slight concussion and shock, but nothing definite developed, and he recovered all right after some days in the hospital.

*Q.* I see; he was only in the hospital about nine or ten days?—*A.* No.

*Q.* What do you think was the matter with his heart?—*A.* He had mitral disease.

*Q.* What was his pulse?—*A.* Very feeble.

*Q.* What did you discover?—*A.* When I examined him I was struck by the weakness of the man's pulse, and examined him for it.

*Q.* Have you anything to add; what was the nature of his injuries; did anything come out more than we have been told?—*A.* No, concussion and shock.

*Q.* Did you lay stress on the condition of his heart to Dr. Macqueen, because the note here about it is "slight concussion and shock"; he was not in very long, and I want to know why were the phenomena of shock and concussion so marked?—*A.* The man, I believe, was struck by a weight of about fifteen hundred-weight on the head.

*Q.* How was he struck on the head?—*A.* I believe it was a crane swinging on him.

The Legal Assessor: I see you did not say anything about the fact that you told them not to wash him; you told them to take care of him and keep him quiet?—I told them to put him to bed, and that he was to be left quite quiet.

*Q.* You did not say that; that was rather a serious breach in your report was it not?—*A.* I understood that patients with those orders are not washed.

*Q.* Quite so, but you did not say that in your report to The Hospital; I think I should have added "after my express orders that he was to be left quite quiet"?—*A.* I did not think that was necessary.

Mr. Procter: Will you tell us, Dr. Shepherd, to clear up the question about the pulse, what was the actual pulse you took?—I cannot remember.

*Q.* Will you tell us to whom you gave instructions?—*A.* I gave instructions to the casualty sister.

*Q.* Do you know who she was?—*A.* Sister Turner, I expect.

*Q.* Did you tell her what you have told us about the heart condition?—*A.* I did not mention the heart condition to her, no more than that the man had an exceedingly bad pulse.

*Q.* You told her that he had a bad pulse?—*A.* Yes.

*Q.* You did not mention about the heart trouble?—*A.* No, not, at least, to my knowledge at the present moment.

*Q.* You took no notes?—*A.* No.

*Q.* This was the subject of an inquiry by Dr. Macdonald and Mr. Gayner?—*A.* I believe it was.

*Q.* Did you give any evidence before them?—*A.* I cannot remember at the present moment.

The Commissioner: Have we any notes of that?

Dr. Macdonald: Yes, in these two books, I believe.

Mr. Procter: Do I understand that you don't remember whether you made any statement at all?—I cannot.

*Q.* Then it is of no use asking whether you told them about the pulse?—*A.* No.

*Q.* Can you remember at what hour you saw him in the casualty ward?—*A.* It would be between 3 and 4 o'clock, or shortly after 4 it might have been, I should say that would be about the time.

*Q.* You cannot fix it any nearer?—*A.* No.

*Q.* You are speaking to ten minutes in the hospital?—*A.* Ten minutes after admission.

*Q.* But you are going to speak definitely about ten minutes, are you not?—*A.* Well, I followed the man up to the ward about ten minutes after he came in.

*Q.* But that ten minutes is to begin between 3 and 4; can you give us some hour when it did begin?—*A.* No, I cannot tell you exactly when the man came in.

Dr. Macdonald: I find on reference to the notes that it was not this case that was brought before us.

### A MATTER FOR THE COMMISSIONER.

Mr. Procter: Can you tell us the exact time when you gave the orders to the casualty sister?

Mr. Baker: I think he has answered that.

Dr. Shepherd: No more than that it was between 3 and 4.

Mr. Procter: How was he taken away?—He was taken up by the porter to Ward 2.

*Q.* Do you know what the process of washing a patient is?—*A.* Yes.

*Q.* How long does it take?—*A.* It varies with the smartness of the nurses; five or ten minutes, or fifteen minutes, and it depends upon the dirty condition of the patient.

*Q.* What is the procedure?—*A.* As a rule the patient is first laid out on a made-up bed and a rug put over him; one nurse with soap and water starts scrubbing the patient down while the other follows and dries as she goes on.

*Q.* Is it a usual rule to wash a patient when brought in?—*A.* It depends on the condition of the patient.

*Q.* Is that left to the discretion of the nurse, or should it be the subject of an express order by the Resident?—*A.* It depends upon the sisters to a great extent.

*Q.* But I mean as a general rule?—*A.* Well, the order is sometimes given and sometimes not.

*Q.* Did you give an order in this case that he was not to be washed?—*A.* I did not give an order that he was not to be washed, but I expressed the desire that he was to be put to bed and left quiet, and I understood from that that he was not to be washed.

*Q.* Don't you think it would have been better if you had given a specific order?—*A.* That I took to be specific enough.

*Q.* You did, but would he not, in the ordinary course, be washed?—*A.* I cannot speak very much of the custom on the casualty side.

*Q.* What would be the custom on the medical side?—*A.* Again, it depends on his condition.

*Q.* I mean if a case is handed over to a nurse or sister, would it be her duty to wash the patient in the absence of any specific directions to the contrary?—*A.* Yes, I expect it would be.

*Q.* And you not having given any specific instructions that he was not to be washed, it would follow that he was washed?

Mr. Baker: Surely that is a matter for argument as to what constitutes "specific" direction.

Mr. Procter: Specific direction would be not to be washed.

Mr. Baker: Specific direction as to whether he is to have certain treatment.

The Commissioner: But Dr. Shepherd's contention is that his instructions that the patient was to be left quiet would carry with it that he should not be washed.

Mr. Procter: I will leave it with you.

The Commissioner: It is a matter upon which I shall have to form an opinion.

Mr. Procter: I want to call the sister and nurse concerned.

The Commissioner: Yes, please. (To Dr. Shepherd): You saw this case in the casualty department, and the case then went into a ward; did anyone go with it from the casualty department?—I expect the sister or casualty nurse would.

*Q.* Would she go with you?—*A.* There is a lift, the ward is upstairs.

*Q.* Then the casualty sister or nurse would go with you, and leave the patient in charge of the sister in whose ward it is admitted. As a matter of fact you went up because you were acting for Dr. Macqueen?—*A.* Yes.

### SISTER HANNAH CALLED.

Sister Hannah was examined by Mr. Procter.

*Q.* Do you remember the case of A. Fernley, on December 24?—*A.* Yes.

*Q.* Were you the sister in charge of Ward 2?—*A.* Yes.

*Q.* Who brought him to your ward?—*A.* The first-aid man from the railway works brought him up.

*Q.* Did any nurse come with him?—*A.* I did not see one.

*Q.* Were any directions brought to you with him?—*A.* Nothing special.

*Q.* Had you any information as to his condition?—*A.* I went and saw the patient and I saw the man who brought him up and he told me about him, how he met with the accident.

The Commissioner: How did it happen, so far as your recollection goes?—*A.* He was hit with a piece of iron on the head.

*Q.* It is suggested that he was struck by a crane?—*A.* Yes.

Mr. Procter: Was any information given you as to the state of his heart?—No.

*Q.* Or his pulse?—*A.* No.

The Commissioner: But the patient came up with an ambulance man?—I saw the nurse immediately and she reported to me as to temperature, and pulse, and respiration.

*Q.* Do you remember what they were?—*A.* Pulse 64, normal temperature, and respiration normal, 23 or 24, I am not quite certain.

Mr. Procter: Does that indicate any weakness of the heart?—No.

*Q.* What is your custom when you see a patient in the ward?

The Commissioner: Will you mind my interpolating a question; the statement is made that he was suffering severely from shock; did you get that impression?—No, I didn't.

*Q.* You are accustomed to surgical cases, shock, and collapse, and you must be familiar with them?—*A.* No, when I saw him I did not think he was collapsed, and I went on with my decorations.

Mr. Procter: What is the usual thing in such cases?—We put them to bed, and the temperature, pulse, and respiration are taken.

*Q.* And what else?—*A.* We knew the patient was coming on and the bed was got ready.

*Q.* And then?—*A.* He was undressed, and got ready for washing. Nurse Bewlay went on washing him.

*Q.* I understand Dr. Shepherd came in during the washing?—*A.* Yes.

*Q.* How long after he had been in the ward did you begin to wash him?—*A.* I cannot say exactly, but over half an hour; we could not get him to bed and prepare him for washing in less.

*Q.* Was there anything in the condition of this patient that would lead you to suppose he was not fit to be washed?

The Commissioner: Of course, there are various ways of washing a patient, are there not?—*A.* Yes.

*Q.* There is hardly a case so serious that you could not wash with special attachments. Assuming this patient was in a condition of serious shock and collapse you would not have washed him in the way you did?—*A.* No.

*Q.* We don't want to go into further details, because it all depends upon the question of evidence as to what condition of shock and collapse he was in, but if he was in such a condition you would never have washed him as you did?—*A.* No.

By Mr. Procter: I have not gathered from you how long after he was admitted that Dr. Shepherd came on the scene?—I cannot say exactly.

*Q.* But according to you it must have been more than half an hour?—*A.* Oh, yes, more than half an hour before Nurse started washing him.

*Q.* And he came in while the washing was going on?—*A.* Yes.

*Q.* Did you hear Dr. Shepherd's statement that he should not have been washed?—*A.* I asked Dr. Shepherd to excuse me, and I met him at the door and inquired how the patient was, and he told me that the patient was very bad, and he was cold and should have more hot bottles. I went straight to the patient, washed my hands, and took his pulse; it was quite a good pulse, a normal pulse.

*Q.* There was a direct difference of opinion between you and Dr. Shepherd?—*A.* I asked Nurse if the pulse had changed since she first took it, and she said "No," and I went straight down to Dr. Shepherd and could not find him in his rooms. I met him about an hour afterwards, and told him that I had been looking for him, and that I went at once when he left the ward and took the patient's pulse, which was then good, and he was quite warm. Dr. Shepherd was quite nice, and said he thought he had better be left till Dr. Macqueen came. Afterwards Nurse told me that Dr. Shepherd had stopped her washing the patient, and that Dr. Shepherd had been upset before he came to the ward.

The Commissioner: I thought you told us, Dr. Shepherd, that you went first to the ward with the Casualty Sister?—I went up after I had sent the Casualty Sister. I gave him time to get undressed and in bed.

*Q.* You did not tell us how long afterwards?—*A.* I said about ten minutes.

*Q.* Then you do not agree with the Sister as to that?—*A.* I cannot say that it was much more than ten minutes, to my mind.

*Q.* There is that difference.

Sister Hannah: It is impossible to undress a patient, get a patient into bed, and wash him——

The Commissioner: I don't want to argue the point; I can form my own opinion as to that.

Mr. Baker: How many were assisting in undressing him?—The First-aid man assisted, as he always does, and the Nurse.

*Q.* There were three then?—*A.* I did not at that time; I was decorating. It was Christmas time.

The Commissioner: Are there any clinical notes?

Dr. Macdonald: It is a surgical case, and there is no surgeon here this morning.

Mr. Baker: I understood you to say that the patient was brought in by the first-aid man, and that you did not see the nurse, and that there were no special directions?—Yes.

*Q.* Did you know Dr. Shepherd gave special directions when he ordered the patient to be removed to the Casualty Ward?—*A.* It is the first I have heard about it.

*Q.* Then you did not subsequently, when the nurse came in, receive directions from him?—*A.* No; only that a patient was coming up.

*Q.* You received no instructions from Dr. Shepherd at all about the case?—*A.* No.

*Q.* Then I want to deal with the pulse. You say it was quite normal, and mentioned 64. Do you call 64 a normal pulse?—*A.* With some men it is; sometimes we have a pulse of 64 which is quite normal.

*Q.* Is not that extremely low?—*A.* It is low.

*Q.* Is not the average about 80?—*A.* 70 I consider normal for a man.

*Q.* Do you consider 80 normal?—*A.* Yes.

*Q.* So that you say 60, 70, 80 are all normal?—*A.* Yes, for some patients.

The Commissioner: Is your suggestion, Dr. Shepherd, that his pulse was unusually slow; is it your view that his pulse was abnormally slow or quick?—It was tending to be slow.

*Q.* And he had mitral disease?—*A.* Yes.

Mr. Baker (to Sister Hannah): You say you were decorating?—Yes.

*Q.* Does that mean that you were not paying great attention to the case?—*A.* I saw the case and my senior nurse saw it.

*Q.* Who is that?—*A.* Nurse Bewlay.

*Q.* But you had had no special instructions?—*A.* No, I had not.

*Q.* Was there any reason why you should make any special examination?—*A.* No.

*Q.* So that the impression you formed, which you have given the Commissioner to-day, is based upon the information given you by Nurse Bewlay as to his pulse and temperature?—*A.* Yes, and I saw the patient myself.

*Q.* But you say you did not examine him?—*A.* No; I left him to be undressed and washed.

*Q.* You just looked at him before giving the orders for him to be undressed?—*A.* Yes, and spoke about him with the man who brought him to the ward.

### NURSE BEWLAY CALLED.

Mr. Procter: You were in Ward No. 2 in December last; do you remember the case of A. Fernley?—Yes.

*Q.* Are you the senior nurse?—*A.* Yes.

*Q.* Did you actually receive the case from the Casualty Ward?—*A.* I do not know whether it came from the Casualty Ward, or straight up.

*Q.* Who brought him up?—*A.* The Porter and a man from the railway works.

*Q.* Did any nurse come with him?—*A.* No.

*Q.* Was any particular report given you of him?—*A.* No.

*Q.* What actually was said?—*A.* The man who came with the patient told me of the accident that happened.

The Commissioner: Did he give you any details of the accident?—He said that he had been struck on the head by a crane.

The Commissioner: The next point is the condition in which you found him when he came in. We have it in evidence that he was suffering from slight concussion and shock; what we want is your observations; did you see any evidence of shock and concussion?—No, there did not appear to be any—in fact, he began to speak once.

*Q.* He did not appear to be very ill? Because, after all, it does not require an expert to know when a man is very much collapsed?—*A.* He did not appear to me to be collapsed.

*Q.* It is a matter of common sense whether a man looks knocked out by an injury; he did not appear to give you that impression?—*A.* No.

*Q.* Tell us his pulse, temperature, and respiration?—*A.* All normal.

*Q.* Were they charted?—*A.* I put them on a slip of paper, and the Sister took it.

*Q.* That paper is likely to have disappeared?—*A.* Sister charts it.

*Q.* And that chart would be the result of your examination?—*A.* Yes, Sir.

Mr. Baker: I understand that she corroborates the Sister that no instructions were received as to washing?

The Commissioner: No; the contention is that he should not be washed if in the condition described by Dr. Shepherd.

Mr. Procter: How long was the patient in the ward before the washing commenced?—About half an hour, I should say.

*Q.* Then how long after you began to wash him was it that Dr. Shepherd came in?—A. We had not started; we were just about to start.

The Commissioner: Do you mean that the patient was uncovered and prepared?—No; he had several blankets and hot bottles round him.

Q. When Dr. Shepherd saw him?—A. Yes.

Q. So if Dr. Shepherd says you were actually washing him and he was uncovered, that is not correct?—A. No.

Mr. Procter: The washing had not begun?—No.

Q. What experience have you?—A. I have been here three years and had five years' previous nursing.

The Commissioner (producing a chart): Does that relate to your case?—Yes, that is the one, Sir.

Then I see at the beginning the pulse is given as 64 and the respiration 24, and when they were next taken it is the same, 64 and 24, and it keeps at about those figures.

Mr. Baker: I think you were decorating?—Yes.

Q. Have you a clear recollection of this incident?—A. Yes.

Q. Very clear?—A. Yes.

Q. What time was it when the patient was brought up?—A. Between four and five in the afternoon.

Q. I want you to put it a bit closer than that?—A. I could not say the exact time.

Q. I ask you, you know, because you said you remembered this very carefully; do you know what time it was when Dr. Shepherd came up?—A. I should say about half an hour after the patient was admitted.

Q. Yes, I know you said that, but what time?—A. I cannot tell you.

Q. Although you cannot tell me either the time when the patient was brought up or the time when Dr. Shepherd came in, you are yet prepared to say quite positively it was about half an hour after?—A. Yes.

Q. Do you know Dr. Shepherd says it was about ten minutes, by which he means, I suppose, it might have been a quarter of an hour; are you prepared to say that that is untrue; are you prepared to say twenty minutes is an impossible time between the doctor coming and the patient being brought in? You give it as half an hour?—A. I do not know, because I did not look at the time.

Q. You put it at half an hour, and you say your recollection is very clear?—No answer.

Q. You cannot help me; would you say that twenty minutes was wrong if it is said that twenty minutes was the time; are you prepared to say that was wrong?—A. It might have been twenty minutes to half an hour.

Q. Are you prepared to say a quarter of an hour is wrong?—A. I could not say definitely.

Q. Are you prepared to say that ten minutes is wrong?—A. It was more than ten minutes.

Q. You are not prepared to say it was more than a quarter of an hour?—A. I do not know between a quarter and half an hour.

The Commissioner: The nurse's evidence is based upon the estimate of the time that would be necessary to undress the case, and she shortens the necessary time because she says that when the doctor arrived the patient was in the blankets.

### SISTER TURNER CALLED.

The Commissioner: You are the casualty nurse?—Yes.

Q. Can you remember the case of Fernley who met with an accident by being struck on the head?—A. I cannot remember it; I get so many cases I do not remember one from the other.

Q. You never heard of the case until this morning?—A. I might have heard of it, but I cannot remember it.

Q. You did not know we were inquiring into it?

The Legal Assessor: You did not remember the case, and nothing was told you?

Mr. Neden (Secretary): I did not know that she was interested in the case until her name was mentioned here.

The Commissioner: This patient was admitted the day before Christmas and was brought up, having been struck on the head by a crane, and was taken from the casualty department into the ward; admitted by Dr. Shepherd and brought to the casualty by a workman; the workman accompanied him and the porter to the ward. Does that enable you to remember anything about him?—No, Sir, I am sorry it does not.

Q. Obviously it is no use asking her more. Dr. Shepherd, you have had an opportunity of seeing these charts?—A. Yes.

Q. I notice on admission his pulse is 60, and it remains at about that and up to 70 until his discharge. That would not suggest the pulse of a man suffering from a large amount of mitral disease?—A. No, Sir.

Mr. Procter: We have it that he was discharged cured on January 2.

The Commissioner: Do you wish to carry it any further?

Mr. Baker: No, I do not think so.

### PATIENTS' COMPLAINTS—THE SLIPPER CASE.

The Commissioner: Then we come to the slipper case, No. 6, to which the note is this—we have no details of it—it is referred to in this book, page 77: "The case of Mr. Hughes; patient taken without slippers to the Secretary's office; Sister Lilly; lack of supervision by Matron." I must trust to Drs. Macqueen or Shepherd to give us any further details.

Mr. Procter: May I shorten this? I think you will find that the Committee inquired into it.

Mr. Baker: How did that arise; an inquiry by whom?

Mr. Procter: By the Committee.

The Commissioner: This is an extract from the House Committee's minutes of October 22, 1912. It is the case of a patient who was taken to the Secretary's office without slippers on her feet. The finding of the Committee is as follows: "It was agreed that the Secretary write Mr. Fawcett, the husband of the patient, thanking him for bringing the matter to the notice of the Committee, and stating that it was evident that some errors of judgment had occurred, and that steps will be taken to prevent a recurrence of the incident." Do you wish to deal with that?

Mr. Baker: Only one thing, there is one point I want to make.

Mr. Procter: There is a letter from Mr. Fawcett——

Mr. Baker: I wish to use this as a specific instance in support of a general allegation which we find later on of responsibility being placed on the wrong person—I forget the number——

The Legal Assessor: That is on page 648.

Mr. Baker: Thank you very much. My instructions are to make this an instance in evidence. I will ask Dr. Macqueen.

The Commissioner: To put this evidence in relation to this particular allegation that responsibility that ought to rest with the sister is put upon the nurses?

Mr. Baker: I want to identify the names, so that I can use it as evidence later on. Dr. Macqueen, I thing this was your case?—Yes.

Q. Do you remember the nurse responsible?—A. The Sister was Sister Lilly, the Nurse was Nurse Sharp.

Q. Do you know whether Nurse Sharp or Sister Lilly was blamed?—A. I believe Nurse Sharp was blamed.

Q. Is there a minute dealing with this? I call for it.

The Commissioner: I have read the entry in the book: "Notes on the inquiry; case of Mr. Hughes; patient taken without slippers to the Secretary's office; Sister Lilly; lack of supervision by Matron."

Mr. Baker: I can put it in this way: my instructions are that Sister Lilly, who was really responsible, was not blamed, and Nurse Sharp was, and I will leave it to my friend to cross-examine.

Dr. Macdonald: We did not go into that very fully because it had become serious and had been fully investigated by another Committee—the House Committee—and I believe there is a report on this, but it is not within my definite knowledge.

The Commissioner: It was not properly investigated?

Mr. Jalland: I believe that that is a report of two ladies of the Special Committee which reported to the House Committee; I believe that that is their report, that extract that you have read.

The Commissioner: We have here the minutes of the Committee, that means that the findings of the Sub-Committee were agreed to by the House Committee.

Mr. Procter : Should we not ask Dr. Macqueen why he considers that Sister Lilly should be blamed.

The Legal Assessor (to Mr. Baker) : Should you not call the nurse; she surely is your witness; you say she is the person wrongly blamed; if she says no, then that is an end of your contention.

Mr. Baker : I have no minutes of the Committee.

The Commissioner : I think if it was a Ladies' Special Committee, you can take it that there were no minutes. (Laughter.)

### HOUSE COMMITTEE'S INQUIRY.

The Commissioner was then handed a minute book, and read the following minutes of the House Committee under date October 22, 1912 :

"The Committee inquired into the circumstances connected with the treatment of a Mrs. Fawcett on the morning of her discharge from the hospital, brought to their notice by her husband. Mrs. Fawcett was admitted on October 2nd, and on the following morning was operated on for appendicitis by Mr. Hughes. She was to be discharged on October 16, and she wrote to her husband to bring her outdoor clothes on that day at 10.30 A.M. Mr. Fawcett was interviewed and told the Committee that he brought the clothes and took his wife home in a cab. On arrival she asked to go straight to bed, as she did not wish to get cold; she further stated that she had been got up that morning about 4 A.M., as the bed was wanted for another patient. She had been put on a couch, wrapped up in blankets, and was comfortable until the day staff came on duty, when the blankets were removed. She felt cold, and another patient wrapped her feet up in a shawl, in spite of which, however, she was still cold. At 10 o'clock one of the nurses put her home stockings on, and she walked to the secretary's office to sign the discharge register without slippers. Since reaching home Mr. Fawcett said his wife had been very unwell, having caught a chill, and on Sunday morning last her temperature was 104. This morning (Tuesday) it was 101. Mr. Fawcett said he did not wish to bring trouble on anyone, but he had been requested to mention the matter by Mr. Hughes, to whom he had related the circumstances, on meeting him, and being asked how his wife was progressing.

"The Lady Superintendent (Miss Tute) was seen, and said that the night nurse had reported that Mrs. Fawcett did not rise till 5.40, and was put by the fire, wrapped up in two blankets. She (the nurse) had received instructions from the day sister to prepare the bed for another patient who was to be admitted on the 16th. Nurse Patchett, the night nurse referred to, was then called in, and said the patient was not got out of bed until 5.40. She began to make the beds at 5 o'clock, and Mrs. Fawcett was the last of three who were going out that day. Her clothing consisted of a dressing-gown, dressing-jacket, and two blankets. She would have difficulty in finishing her work if the patients were not got up very early, and the usual time for commencing to make up the beds was 5 o'clock. Sister Lilly, the day sister, was then questioned, and said she saw Mrs. Fawcett on a couch near the fire when she came on duty at 8 A.M., and told a nurse to take away the blankets and wrap the patient in a rug in order to make room to clear up the ward. The patient at this time had stockings on. At about 10 o'clock she took some other patients to the secretary's office to sign the register, and told Nurse Sharp to get some slippers for Mrs. Fawcett, and she met her at the ward door and brought her to the office, but she did not notice if the slippers were on. She had left word the night before for the bed to be prepared for a new patient, but she did not know what time the said patient would be coming in. Nurse Sharp, the day nurse, was then interviewed, and said Mrs. Fawcett had her stockings put on about 8 A.M., but she did not know if she wore slippers, and she did not notice if she had any on when she took her to the ward door to meet the sister who was to take her on to the office. Nurse Sharp was censured for not ascertaining if the patient had slippers on before she left the ward. It was agreed that the secretary write to Mr. Fawcett, thanking him for bringing the matter to the notice of the Committee, and stating that it was evident that some errors of judgment had occurred, and that steps would be taken to prevent a recurrence of the incident. The opinion was also expressed that it was very undesirable that patients who were to be discharged should be got out of bed so early as 5 A.M. the same morning, particularly in cases where serious operations had been performed.

The Commissioner : Well, I am afraid it is not within the scope of my reference to go into that, but it appears to me that the Committee dealt with the matter in a most proper manner, and with every consideration for Mr. Fawcett.

Mr. Neden : That was our intention. There is a further minute at the next meeting.

The Legal Assessor : It says that Nurse Sharp was censured; will not that satisfy you, Mr. Baker?

Mr. Neden read the following extract from the minutes of the meeting of the House Committee dated November 12, 1912 : "A letter was read from Nurse Sharp referring to the complaint from Mr. Fawcett investigated at the last meeting. Nurse Sharp said she was not prepared for the inquiry when she was before the Committee, and that a statement had been made respecting her conduct which was incorrect. The matter was referred to Mrs. Morrell and Mrs. Gayner to deal with."

Mr. Baker : I am not persisting in a charge against the authorities except to support other general allegations, that in fact Nurse Sharp was blamed instead of Sister Lily, who was really responsible.

The Legal Assessor : Surely Sister Lily was also held responsible.

Mr. Baker : I did not understand it so.

Mr. Neden read a further minute of a meeting of the House Committee on November 26, 1912, as follows : "With reference to the complaint *re* Mrs. Fawcett, Mrs. Morrell reported that along with Mrs. Gayner she had interviewed Nurse Sharp, and they had come to the conclusion that there had been some slight oversight with regard to the attendance on Mrs. Fawcett on the morning of her discharge. Mrs. Morrell had visited Mrs. Fawcett at home, who said she was quite satisfied with the operation and general attention, but felt that she should not have been taken to the office to sign the discharge register without slippers."

Mr. Baker : I am not saying this was a very serious breach of duty on the part of the hospital authorities towards the patients; that is not my point at all.

The Commissioner : It is this, that in several instances responsibilities which ought to rest with the sister were attempted to be put on the nurse. Now the facts are that the case was very thoroughly investigated by the House Committee and that they arrived at a certain conclusion. Is it possible to reopen it? The evidence before us is that they took infinite pains to get at the facts; that certain irregularities had occurred. The evidence seems to be on one side. The sister told the nurse to get the patient ready, and the sister, if brought before you, would probably say that that included putting the slippers on, and that they did not do so; and the nurse would say she did not understand she had to put them on, or thought they were on, or that she forgot them. It appears to me that the Committee dealt with it quite properly, and that you cannot carry it further.

Mr. Baker : Very well then.

The Commissioner : Then we take out No. 6 charge.

### SPECIAL NURSING.—THE DIPHTHERIA CASE.

We now come to No. 7, "A case of diphtheria on which tracheotomy had been performed, and which required special nursing: A nurse was sent as 'special' who had never seen a tracheotomy case before, while one who had had fever training and nursed many similar cases was going about that night doing little or nothing. Result: Membrane was allowed to accumulate in the tube which was coughed out of the wound, and the nurse was unable to replace it. We were urgently called to the case several times because of this, and in the end a second tracheotomy (low) had to be performed."

*Q.* Who was in charge of this case?

Mr. Baker: Is this your case, Dr. Shepherd?

Dr. Shepherd: Yes.

The Commissioner: May I read out some evidence of yours, Dr. Shepherd, to see whether you agree with it? This is in the note-book No. 1 of evidence given before the special committee of the Medical Board:—

"H. P. reports case of tracheotomy, now in Ward 3, states that he informed Matron that the case was one of special difficulty. He considers it very regrettable that a nurse was put in charge of this case who had a good general competence, but lacked the special training of the nurse who was doing night extra that night. In the event, both Residents were up with the case till 4.30 A.M."

Then Dr. Macdonald makes a note that the evidence was read over to Drs. Macqueen and Shepherd, and they agreed that it was a correct abstract.

Dr. Shepherd: Yes, that is correct.

The Commissioner: Do you wish to amplify that, Dr. Shepherd?

Dr. Shepherd: I have no wish to amplify it any more than that I considered the case a particularly severe one, and that I considered that the choice of such a "special" in that case was an exceedingly bad one. I had considerable trouble with it.

*Q.* Who was the "special"?—*A.* Nurse Mouls.

*Q.* Particularly in view of the fact that you thought a more experienced nurse was available, I take it?—*A.* Yes, one who had nursed tracheotomy before.

*Q.* Yes, one of special training?—*A.* Yes.

*Q.* Will you tell me what operation was performed?—*A.* An ordinary tracheotomy.

*Q.* Where?—*A.* Through the first and second rings of the trachea, the operation of high tracheotomy. I performed the first. Below the level of the thyroid.

*Q.* I just want to know where. And the low level tracheotomy took place?—*A.* Yes, below the level of the low thyroid.

*Q.* Did you have much difficulty in inserting the tube after the first tracheotomy?—*A.* No; the thing had to be done immediately.

*Q.* Were you sitting up with the child?—*A.* I was afterwards. I sat with the child, I should say, from any time after three until half-past four, and I saw it several times in between.

*Q.* How was it possible for the tube to be coughed out of the wound?—*A.* I cannot understand. The child was very restless, and, as it was exceedingly ill, I suppose the constant rolling about got the tapes unfastened and the coughing coughed the tube out. I cannot explain how it did come out.

*Q.* It is not possible to cough the tube out if it is properly fastened, is it?—*A.* No.

*Q.* And, of course, for the fastening you were responsible?—*A.* For the fastening I was responsible.

*Q.* The nurse did not herself remove the whole tube, I suppose.—*A.* Not to my knowledge.

*Q.* You say in the result membrane was allowed to accumulate in the tube?—*A.* Yes, but there seemed to be very few feathers in the hospital that night. She did not know the exact technique of feathering.

The Commissioner: Have you any questions, Mr. Baker?

Mr. Baker: No; any questions I have I think I had better postpone for re-examination after Mr. Procter's questions.

Mr. Procter: Well, I don't know that I have very much to ask after what he has given you. The only point, I understand, you make is that the nurse had no previous experience of tracheotomy. That is all?—Yes. The point is that the choice of such a nurse was a bad one, in view of the fact that there was a good nurse who had nursed similar tracheotomy cases before.

Mr. Baker: Available?—Available.

Mr. Procter: Did you, at the completion of the case, compliment the nurse on the way she had looked after the patient?—When the more serious part of the case was over, after the tube had to be taken out entirely, I did compliment the nurse on the latter part of her treatment, but certainly not on her former. It was her first case. She had done very well considering, but certainly was nothing like competent for such a case.

*Q.* You did not blame her?—*A.* I did not blame her, because she had no knowledge of such a case.

*Q.* It is a question of fastening the tube?—*A.* Not at all.

The Legal Assessor: May I suggest, from your point of view, I don't see how you could improve on the questions put by Sir Cooper?

Mr. Procter: I agree. I want to call Sister Wright.

The Commissioner: Is she the sister of the ward? Was this in the Isolation Ward or the General Ward?

Mr. Procter: Ward 3.

### THE MATRON CALLED.

The Commissioner: Now, Matron, we are investigating an allegation that is made by the Residents with regard to a case of diphtheria. I may as well read it, though you are no doubt familiar with it. [Paragraph from THE HOSPITAL read. I presume the Matron is called on the nursing question.

Mr. Procter: That is so, sir; the suitability of the nurse.

The Commissioner: The suitability of the nurse. Perhaps you had better ask her.

Mr. Procter: Matron, it is suggested by Dr. Shepherd that you sent him a nurse without any previous experience of tracheotomy for this case, when you could have sent one who was doing little or nothing?

The Matron: I don't know who the available nurse was at all.

The Commissioner: What was the name of the nurse who, having special experience, on that night was going about doing little or nothing?

Dr. Shepherd: Nurse Hunt.

The Matron: I think she was night nurse in Ward 2 on that night.

Mr. Procter: Does that mean she was not available for this case?

The Matron: She could have been available if I had put her there, but I like my senior nurses to have experience, and give them experience when I can. Nurse Hunt had had children's training in London, and did not come here for children's training. The nurse who was put on was the senior nurse.

*Q.* That was Nurse Mouls?—*A.* Yes.

*Q.* What do you say of her competence for tracheotomy?—*A.* Simply that she was senior nurse, and I wished her to have experience before finishing her training. Naturally the nurses come here for training and for experience. They must all have a case for the first time, and they work under the night sister.

*Q.* Were you told that this was a specially urgent case of tracheotomy?—*A.* No; I put the "special" on before I saw the doctor at all. I heard there was a case, and arranged for a "special."

The Commissioner: Obviously it must have been a very urgent case, because tracheotomy was done in the casualty room?—Yes, and hearing that I arranged for the night

"special." I always endeavour to give my senior nurses a case of that kind before they leave.

The Commissioner : The Matron's argument is perfectly plain—that she had in the building a nurse with special experience in tracheotomy : that is true enough. She does not admit for a moment that she had any nurse going about doing little or nothing, either that night or any other night, but that nurse—Nurse Hunt—was occupied in a ward. That she put on a nurse who was in training, and who, amongst other items of training, it was desirable to instruct in a tracheotomy case; and that she believed that she had a general competence, though she had no special experience. Is that your view?—That is so. I always look in my book to see who has had a case of that kind, and endeavour that they should have a case, if possible, before finishing their training.

Mr. Procter : It is also said that there was an insufficient supply of feathers that night in the hospital. What do you say to that?—It is the first time I have heard of it.

The Commissioner : Matron would not be requisitioned for feathers, I suppose. You were not requisitioned for feathers in the middle of the night?—No; I have heard nothing about it until now.

Mr. Baker : Do you know how many patients there were in Ward 2 that night?—I could not say without seeing the night report. There would probably be between twenty and twenty-six, but I cannot speak for that night.

Mr. Baker : If you don't know, you don't know. I only wanted to ascertain first of all.

The Commissioner : Her argument is not that she could not have taken Nurse Hunt out of the ward if she had so desired.

Mr. Baker : Your object in putting Nurse Mouls to deal with this case was that she might have experience of it?—Certainly.

*Q.* You wanted her to have experience of it?—*A.* Before finishing her training; yes.

*Q.* Do you know whether she had ever had experience of cases of this kind before?—*A.* Only the cases that she has heard of since she has been in the building. She had not nursed a case.

### NURSE TURNER CALLED.

The Commissioner : We have seen you before, this morning. Now we are asking you about something that no doubt you have a better recollection of. It is about this case of diphtheria on which Nurse Mouls was "special." I don't know precisely what the sister is called for, so I will leave Mr. Procter to ask the questions. Oh, yes, this case you do recollect. It was a child that was brought in, I suppose with urgent dyspnœa, breathing very badly.—*A.* Yes.

*Q.* And Dr. Shepherd was called to see it?—*A.* Yes.

*Q.* And it was so ill, and the symptoms so urgent, that the trachectomy was performed in the casualty room?—*A.* Yes.

*Q.* Was the operation a specially difficult one? It could not have been easy, because the child was struggling, I suppose. Did the child have an anæsthetic?—*A.* No, sir.

*Q.* The tube went in pretty easily, or not?—*A.* Yes, fairly easy. The child was struggling.

*Q.* And then the child was taken from the casualty room with the tube, and the tube was tied in, of course?—*A.* Yes.

*Q.* And from that point you know nothing?—*A.* No, sir.

### SISTER WRIGHT CALLED.

Mr. Neden : Sister Wright is the ward sister who received the patient from the casualty room.

The Commissioner : At what time did the patient come in?—Sister Wright : Five minutes to eight.

*Q.* In the evening?—*A.* Yes.

*Q.* And what time did you go off duty?—*A.* We are supposed to go off at twenty past eight, but I was not off until after nine that night.

*Q.* So that the patient was under your eye from 7.55, do you say, to half past nine or a quarter past nine?—*A.* Yes.

*Q.* And the "special" was there as soon as the patient got into the ward or thereabouts, I suppose?—*A.* Afterwards. I was "special" until half-past eight.

*Q.* She was the night "special"?—*A.* Yes.

*Q.* So that you overlapped with her duty between 8.30 and about 9.30?—*A.* Yes.

*Q.* You were on together?—*A.* Practically. I left her in the side ward at half-past eight, you see.

*Q.* What did you yourself see of this child; I mean how much attendance on the child did you do yourself?—*A.* When the child came in, I put it to bed. It was breathing very badly. I put in hot-water bottles, got a fire on. I asked the doctor if the tube was all right, and he looked and said "Yes." They had not gone five minutes when the tube came out.

*Q.* How did the tube come out? By what agency did the tube come out?—*A.* It was breathing so badly.

*Q.* But when you say the tube came out : a tube can come out, or be taken out. Which do you mean?—*A.* It came out, of itself.

*Q.* The child might have pulled the tube out?—*A.* It did not.

*Q.* I am only asking.—*A.* Yes.

*Q.* You say that did not happen?—*A.* No.

*Q.* How did it come out?—*A.* It was so blocked up, I think. It did not seem to be fixed tight enough. It was too loose.

*Q.* In other words, you think it was not tied in properly?—*A.* Yes.

*Q.* Of course, you could not cough out a properly tied-in tube, could you?—*A.* No.

*Q.* But it was not that the child was left unattended and pulled it out?—*A.* No, Sir.

*Q.* But that somehow or other the child, by its efforts in coughing, got the tube out?—*A.* Yes.

*Q.* And therefore presumably it was not properly tied in?—*A.* Yes, Sir.

*Q.* Then the breathing was very bad. Did membrane accumulate in the tube? Did you have to use feathers?—*A.* I hadn't got any.

*Q.* There were no feathers?—*A.* No. I sent a nurse for feathers. It was just brought in without anything. We did not keep the feathers in the ward. They were downstairs, where the patient was done.

*Q.* Oh, the dilators and instruments had gone down into the casualty room?—*A.* Yes, and they had not come up.

*Q.* And you had no feathers. What did you do to try and get the mucus out?—*A.* I did nothing. I was by myself. I allowed the child to sit up and put its arms over its head and cough, and I got the tube in.

*Q.* The tube had actually come out?—*A.* It was right out. Yes, Sir.

*Q.* You got it in, then, without any assistance from the Residents?—*A.* Yes, before they came the tube was in, and it was breathing better then.

*Q.* So that really no feathers arrived? Did any feathers arrive while you were still on duty?—*A.* Oh, yes, they came up. Nurse was bringing them.

*Q.* Was there any lack of them for the rest of the night?—*A.* No, Sir.

*Q.* As far as you know. Of course, you went off at half-past nine?—*A.* Yes.

*Q.* You cannot speak to anything later than that?—*A.* No.

*Q.* Do you know who the night sister was, whether she came round to look at the case?—*A.* Yes.

Mr. Baker : Was the child restless before the tube came out?—Not very restless.

*Q.* Were you watching it all the time?—*A.* Yes, I was standing by it.

*Q.* So that the tube came out underneath your eyes?—*A.* Yes.

*Q.* Did you examine the tube before it came out?—*A.* Yes.

*Q.* To see if it was properly put in?—*A.* Yes, I had asked the doctors if it was all right.

*Q.* Yes, I understand that, and then the doctors went away. But after they left, did you yourself examine it to see if it was all right?—*A.* No, I don't think so.

*Q.* I am not suggesting that you should. I am only asking you, did you yourself examine it to see if it was, in your opinion, rightly put in, or securely put in?—*A.* Not when they said it was all right.

*Q.* That is what I want. You think I am trying to catch you, but I am not. You did not look yourself to see that it was in. The next thing that happened was that it came out?—*A.* I had looked before they went and saw it was all right.

The Commissioner: A tube may be not properly in, and yet, to a sister or anybody else who looks at the throat, the tube seems as if it was properly in. I think she is answering your questions quite fairly. She could not possibly know by mere inspection whether the tube was in properly or not. She might say "I don't think it is in properly, because the child is doing so badly." She might say, "It is not tied properly."

Mr. Baker: I wanted to give her an opportunity of saying anything of that kind. But I think I may take it now that you did not make any observation yourself, after the doctor has left, which showed you that the tube was improperly or insecurely fastened?—It looked loose, but then they said it was all right.

*Q.* Yes, but after the doctors had gone. I am confining my questions entirely to the time after the house surgeon had left you; between that time and the time the tube came out?—*A.* Between that time it was only five minutes, and I was watching the tube all the time. I did not have time to watch anything else, because I was trying to keep the child quiet.

*Q.* Was the child restless, then?—*A.* It was restless all the time. It was breathing so badly.

*Q.* Then you did not notice yourself, after Dr. Shepherd had left, that the tube was put in badly. That is right, isn't it?—*A.* I don't say the tube was put in badly.

Mr. Baker: Then the answer is "No."

The Commissioner: The answer is "No," but unfortunately the next item is that in five minutes' time the tube came out, and therefore she has some difficulty in saying that the tube was rightly in.

Mr. Baker: The next question is that that arose from your presumption that it had not been properly put in.

The Commissioner: It may have been put in properly, but not tied properly.

Mr. Baker: I think it is clear in your mind, sir, that the nurse did not make a definite examination, and I don't think she does accuse the doctors. She has given her evidence very fairly, but at any rate I have satisfied you that there is no evidence yet, from her at any rate, that the tube was improperly put in.

The Commissioner: I don't wish you to ask me what conclusion I have come to.

Mr. Baker: I don't wish to ask that. But is there evidence? I mean the suggestion was made, and as far as I could see there was no data given on which the suggestion was formed.

The Commissioner: I don't tell you what conclusion I have come to, but in a tracheotomy case it is impossible for a child to cough out a tube which is properly put in. The tube may have been in its proper position but not properly tied, and therefore the difficulty we are in is how that tube managed to come out if the evidence of the sister is that the child could not itself have pulled it out. I don't see that your questions can carry it further.

### NURSE MOULS CALLED.

The Commissioner: Now you were the special on this case?—Yes.

*Q.* What time did you take the case over?—*A.* 8.20 I went to the bedroom.

*Q.* And what was the condition of the child then?—*A.* The child was very ill.

*Q.* I mean as to breathing?—*A.* Its breathing was difficult.

*Q.* Very difficult?—*A.* Yes, the child was very restless.

*Q.* You had had no previous experience of tracheotomy cases?—*A.* No.

*Q.* What general training had you had?—*A.* I had been here two years and eight months.

*Q.* So you were in your third year, and your general experience was considerable; but of tracheotomy this was the first case you had had?—*A.* Yes.

*Q.* Did the tube become very much blocked with mucus and membrane?—*A.* I feathered it frequently, and took it out and cleaned it.

*Q.* Had you plenty of feathers to go on with, or were the feathers very short that night?—*A.* No, I had plenty of feathers.

*Q.* You know you would not forget the first case. We never forget the first case of anything, either students or nurses, so you would be sure to remember whether you had plenty of feathers. Did the outer tube come out while you were in charge?—*A.* Yes.

*Q.* How do you think that came about? How did the child manage to get the tube out?—*A.* I don't know; it just came out.

*Q.* It seemed to cough it out?—*A.* Yes.

*Q.* How long was it after you had been on duty that the second operation took place?—*A.* I don't know; a good time.

*Q.* Was it several hours after you had been on duty?—*A.* It was about eleven.

*Q.* Well, you came on duty to see the child about 8.20, and it was something like three hours after?—*A.* Yes.

*Q.* Was the second operation pretty easily done?—*A.* Yes.

*Q.* Did the child have an anæsthetic for the second operation?—*A.* No, Sir.

*Q.* Did the tube come out again after the second operation?—*A.* No, Sir.

*Q.* The tube came out not at all after the second operation?—*A.* No, Sir.

*Q.* But before the second operation was done it did come out, but that you cannot explain.—*A.* No, Sir.

*Q.* You are clear that the child did not pull it out?—*A.* No, Sir.

*Q.* Where was the tube tied?—*A.* Round its neck.

*Q.* Certainly; where were the knots?—*A.* At the side.

*Q.* Here? [pointing].—*A.* I think so.

*Q.* You didn't notice particularly?—*A.* No, I am not quite sure.

*Q.* It was your first case and you did not notice?—*A.* No.

Mr. Baker: Do you remember if you had to alter the strings at all?—I tied it when Mr. Macqueen put the tube back.

*Q.* There was a good deal of blood, wasn't there?—*A.* The second operation?

*Q.* Yes. I am dealing with after 9.30. You came on about 9.30.—*A.* No; 8.30.

*Q.* But didn't the strings get covered with blood from time to time, do you remember?—*A.* No, I don't remember.

*Q.* You don't remember whether you did the strings at all?—*A.* No, I did not have to change the strings.

*Q.* Not at all?—*A.* Quite sure.

The Commissioner: You never changed the strings at all?—No.

Mr. Baker: I don't mean putting in new strings; I mean any alteration, re-tying. Did you have to re-tie the strings?—No.

*Q.* You did not have to touch the strings at all?—*A.* No.

*Q.* I thought you told us that when the tracheotomy tube was replaced by Dr. Macqueen you tied the strings?—*A.* Yes.

*Q.* That was after the second operation?—*A.* Yes.

The Commissioner: And after that the tube did not come out?—No.

*Q.* Did the child recover? I understand that it did.—*A.* Yes.

Mr. Procter: How long was Dr. Shepherd with the child?—When?

*Q.* At any time. I mean did he stay in the ward with the child for any considerable time?—*A.* Not for a considerable time.

*Q.* How long did he stay?—*A.* He was in the ward when I came on, and he was in the ward at the second operation.

*Q.* Was he in the ward between those times?—*A.* I don't remember that he was.

*Q.* What period was he in at any one time?—*A.* I don't exactly know how long; it was not long.

*Q.* What do you mean by not long?—*A.* He stopped a long time after the theatre operation.

*Q.* How long do you say?—*A.* Dr. Macqueen and Dr. Shepherd both stopped.

*Q.* How long?—*A.* I don't know exactly how long.

Mr. Baker: Wasn't Dr. Shepherd sitting on the bed a long time and using the feather?—Do you remember that? —That was after the child was taken to the theatre. That was the theatre operation, not the incision in the ward.

*Q.* Yes, but that was during the night.—*A.* Yes.

The Commissioner: I don't quite understand that, nurse. Where was the second tracheotomy done then?—There was an incision made.

*Q.* When I say where, I don't mean whereabouts in the child's neck, but whereabouts in the hospital?—*A.* In the ward one was, and one was done in the theatre later that night.

*Q.* Were there three operations, because the child was first tracheotomised in the casualty room?—*A.* And then the incision was made larger in the ward between eleven and twelve.

*Q.* And then—tell me about the third; I did not understand Dr. Shepherd in his evidence—then the child was taken into the theatre?—*A.* Dr. Shepherd was doing a round in the ward, and come into the ward, and I said the child's breathing was distressed; it was not breathing through the tube. Dr. Shepherd asked me if the tube was clean. I said I had just had the tube out. So I put my hand in front of the tube to see if the child was breathing, and Dr. Shepherd said, "Put a feather in front."

*Q.* But was this before the incision?—*A.* This was after the incision.

*Q.* The child comes into the ward with tracheotomy done, and there it is for a certain time, and then, as I understand it, the incision is made longer?—*A.* Yes.

*Q.* And is that the time you are alluding to?—*A.* No, it went to the theatre afterwards.

*Q.* How long after the incision had been lengthened was it before the child was taken to the theatre?—*A.* About two hours, I should think. I am not certain as to the exact time.

*Q.* And then it went to the theatre?—*A.* Yes.

*Q.* And who operated in the theatre?—*A.* Dr. Macqueen.

*Q.* So the first tracheotomy was Dr. Shepherd's; the incision was lengthened by?—*A.* Dr. Macqueen.

*Q.* And the third operation, if we count the lengthening of the incision an operation, was done by?—*A.* Dr. Macqueen.

*Q.* I think perhaps we had better hear what Dr. Macqueen has to say.

### DR. MACQUEEN CALLED.

Dr. Macqueen: The second one to which the nurse refers was merely an opening of the dilator to get the tube in.

*Q.* Which had come out.—*A.* Yes.

*Q.* It was not an incision then?—*A.* No, it was a dilatation; the ordinary dilator.

*Q.* There is a great difference isn't there, between prolonging a cut in the neck and——

Nurse Mouls: There was a larger incision made in the ward.

Mr. Baker: Do you know what time it was?

Nurse Mouls: It was between eleven and twelve. Two other doctors were present, Dr. Conan and another doctor.

*Q.* In the ward?—*A.* Yes.

*Q.* And who were those doctors?—*A.* They were strange doctors.

*Q.* They were visitors?—*A.* Yes.

*Q.* Friends, do you mean?—*A.* Friends of Dr. Macqueen and Dr. Shepherd.

The Commissioner: That is the first we have heard of that. Then your evidence is that it was not a mere matter of putting in dilators, but that actually the incision was lengthened by the use of a cutting instrument by Dr. Macqueen in the ward?—Yes.

*Q.* That I understand you deny, Dr. Macqueen?

Dr. Macqueen: It was merely a dilatation.

Mr. Baker: Well, Dr. Shepherd was there. Would you like to ask him his recollection?

The Commissioner: Yes, I think we will.

Mr. Baker: Afterwards.

### WHAT HAPPENED IN THE THEATRE.

The Commissioner: Would you go on with what happened in the theatre, Dr. Macqueen?

Dr. Macqueen: I think it was between 3 and 4 o'clock in the morning that I had to perform a low tracheotomy in the theatre. I had just gone to bed when Mr. Shepherd came for me and said that nurse had told him that the tube was out again. He asked me to go up and see the case. The child was breathing so badly even after the tube had been inserted—there seemed to be an accumulation of something in the throat—that I asked nurse to take the child to the theatre, and put in a tracheotomy tube either two or three rings lower.

*Q.* You continued the incision in the trachea, did you? —*A.* Not in the trachea, in the neck.

*Q.* Did the tube on your second operation go into a fresh opening entirely in the trachea?—*A.* Yes, a fresh opening.

*Q.* You did a second tracheotomy?—*A.* Yes.

*Q.* Then perhaps may I ask you, in your own mind, what rendered the second tracheotomy necessary?—*A.* There seemed to be an accumulation in the trachea which it could not get rid of.

*Q.* So that really the second operation, you took it, was due to some blockage of the trachea?—*A.* Yes.

*Q.* It was not that it appeared that the breathing was unsatisfactory after the first operation?—*A.* Oh, no.

*Q.* You did not think it was any fault of the first operation?—*A.* No. I was present at the first operation, which Mr. Shepherd did, and the child was very much relieved after it. The child was scarcely able to get breath when admitted to the hospital, and after the operation it breathed comparatively easily.

*Q.* And what was your explanation of the tube coming out, as it seems to have done on more occasions than one? Why in your mind was it that the tube came out after the first tracheotomy on several occasions, and with your lower tracheotomy you were much more successful in keeping the tube in?—*A.* After the second one the child was much quieter. After the first the child was extremely restless, tossing about in the bed and coughing a great deal.

The Commissioner (to Dr. Shepherd): Really, all I think I am asking you is this: there is a question of whether this incision was prolonged or whether it was merely putting the dilators into the trachea?

Dr. Shepherd: It was merely a putting of the dilators in, and the incision naturally seemed to lengthen because the thing was stretched.

*Q.* Or did it seem to broaden?—*A.* Broaden. Of course, it would not actually lengthen.

Mr. Baker: The only question is, if you put it precisely to him, did he in fact make a longer incision?—No.

The Commissioner: No. His evidence is not. You hadn't a scalpel in your hand?—No.

Nurse Mouls: When the child went to the theatre the tube was in. The tube was not out then. That was the time Mr. Shepherd came into the ward, and I told him the breathing was distressed. He asked me if the tube was clear, and I said, "Yes, I had just cleaned it." The child went blue, and Dr. Shepherd said, "I will fetch Dr. Macqueen.

The Commissioner: You have no explanation, Dr. Shepherd, of how it should be that if the tube was entirely free the child was breathing so badly?

Dr. Shepherd: There was an excessive amount of membrane in the trachea, and it was continually getting blocked. The membrane had really got below the tube, and the tube was getting continually blocked.

*Q.* That was a position that the nurse could not get at?—*A.* Well, no, except by continual feathering.

The Commissioner (to Mr. Procter): Don't you want to ask about the complimentary remarks?

Mr. Procter: Well, it was admitted by Dr. Shepherd. (To witness): What did Dr. Shepherd tell you at the conclusion of the case?—He simply said, "Thank you very much for nursing the case. It is quite all right."

### SISTER HUTCHINS CALLED.

The Commissioner: We are asking you to tell us what you know about this tracheotomy case? Did you see it at all?—Yes, Sir; I saw it frequently in the night.

*Q.* Had you any opportunity of watching the nurse in charge?—*A.* Yes, I had the first time I was up. I was rather busy that night.

*Q.* Can you give any time as to when you saw the child?—*A.* I saw the child at 8.30.

*Q.* That was just when the "special" came on?—*A.* Yes; and I saw it again at 9, and then again between 10 and 11, and 12.30 was the next time.

*Q.* Were you present at the operation in the theatre?—*A.* Yes, Sir.

*Q.* Were you present in the ward when, according to the nurse, the incision was lengthened, and when, according to the doctors, the dilator was put in?—*A.* Yes, I was there then; that was 12.30.

*Q.* What did you see at that time? Because there is a direct conflict of statement as to whether an instrument was used, whether a scalpel was used to lengthen the incision, or whether all that was done was the insertion in the wound of dilators. What is you evidence about that if you were there?—*A.* Well, an incision was made. There were rather a lot round the bed; but there was an incision made, a larger incision. Dr. Shepherd kept saying to Dr. Macqueen to make it larger, and during that time a piece of wool or something came out of the tube.

*Q.* There was a crowd round the bed, the crowd consisting of the "special," Dr. Macqueen, and Dr. Shepherd, and some friends of theirs?—*A.* Yes, two friends.

*Q.* Two doctors, presumably, or friends anyhow?—*A.* Yes.

*Q.* Are you quite sure that you saw a knife used, a scalpel used, to prolong the incision?—*A.* Well, the scalpel was soiled. I was picking up the things—they were putting them on the fire—trying to catch them in the receiver—and I understood that the wound had been lengthened.

*Q.* You actually saw a scalpel?—*A.* Yes.

*Q.* You are clear about that?—*A.* Yes, I am certain.

*Q.* A scalpel which to your eye had been used?—*A.* Yes, it had been used; it was soiled.

*Q.* Now let us hear about the cotton-wool?—*A.* Well, Nurse cleared away the things, and what we thought was a large piece of membrane was found, and I put it away in a hurry with some carbolic, at least the Nurse did. We thought it was membrane, and it was shown to the doctor the next day; but it was discovered it was cotton-wool, and they thought it had been coughed up at the time. That is what I understood, that it came from the wound at the time.

*Q.* You have seen membrane, haven't you?—*A.* Yes, I have.

*Q.* It is not remarkably like cotton-wool in a general way, is it?—*A.* It looked as if it might have been part of the trachea. That is what Dr. Macqueen said at the time. He thought it was, I believe.

*Q.* But Mr. Macqueen did not say it was part of the trachea, did he?—*A.* At the time I think they had some doubt about what it was.

*Q.* I thought you said this was found on the floor, and it was shown next morning?—*A.* Yes; the Day Sister showed it to the doctors, and I believe that they, at least I understood that they, thought it was.

*Q.* What?—*A.* Part of the trachea, or something, till they pulled it to pieces and found it was cotton-wool?—That is what I understood.

The Commissioner (to Mr. Baker): Do you wish to ask any question?

Mr. Baker: I don't ask any questions. I don't attach sufficient importance to it.

The Commissioner: I am not suggesting you should.

Mr. Baker: Do you mean to ask about the friends who were there?

The Commissioner: Well, I did not think it was important. There were friends there. Possibly the friends might have obscured the view the Sister had of the operation.

Dr. Macqueen: The end of the scalpel was used as a dilator—the flat end, not the knife end.

Mr. Baker: Who was the doctor who was there? Your friend?

Dr. Macqueen: Dr. Conan.

Sister Hutchins: Nurse was holding the childs head at the time, so she would have a good view.

The Commissioner: Yes, the Nurse's evidence is that the incision was prolonged, and that the scalpel was used. We have now found that there was a scalpel on the scene. This is the first intimation we have had of that. What is the handle of the scalpel? A metal handle?

Dr. Macqueen: Yes.

The Commissioner: It is, of course, possible for anybody seeing the use of it to have not exactly seen what was taking place, and seeing a metal handle put into the wound, to think the cutting edge was put into the wound.

Sister Hutchins: But it was the blade that was soiled.

The Commissioner: Yes; well, there was a good deal of blood about—must have been, you know.

The inquiry was then adjourned for luncheon.

### PROBATIONER IN CHARGE OF CHILDREN'S WARD AT NIGHT.

When the proceedings were resumed,

The Commissioner said: Well, then, Mr. Baker, we are proceeding now to No. 8 on page 647: "A nurse who had only had four months' training was left in charge of the Children's Ward at night." We have no particulars, and I have no particulars in any of the correspondence except that, so I will ask you to call your witness.

### DR. SHEPHERD CALLED.

Mr. Baker: Do you remember what happened in this case? Do you remember when it was, first of all?

Dr. Shepherd: It was some time, I think, after the Christmas holidays. I can't remember the exact date.

The Commissioner: Which Christmas?—Christmas of last year.

Mr. Baker: Was a nurse left in charge of the Children's Ward?—She was a probationer of between three and four months' standing in charge of the ward at night.

*Q.* Was it the Children's Ward?—*A.* Yes.

*Q.* What was the name?—*A.* Nurse Dann.

*Q.* Do you know how many patients there were in the Children's Ward?—*A.* The ward was full.

The Commissioner: How many patients?—Either 22 or 26. I cannot remember the exact number; its full complement.

Mr. Baker: Do you know if there were other nurses available, and in particular whether there were two nurses in the Isolation Ward who could have been sent to the Children's Ward?—Yes, it was possible. (Dr. Shepherd was understood to add, "Certainly it would have been rather hard lines.")

Mr. Procter: I don't know whether the Matron ought to be here to hear this.

The Commissioner: I don't mind.

Mr. Procter: It might save a little time.

Mr. Baker: Anything to save time, but do you want me to stop this examination?

The Commissioner: No, I don't want you to stop. (To the Secretary): You will get the Matron. (To Mr. Baker): Go on with your examination.

Mr. Baker: Do you know how long Nurse Dann was in charge of the Children's Ward?—Part of that night and I think the whole of the next night.

*Q.* Two consecutive nights?—*A.* Yes, as far as I can remember.

*Q.* Can you tell me the names of the two nurses who were in your mind when you suggested that they should be brought over?—*A.* Nurse Thompson and Nurse Bewlay were in the Isolation Ward.

*Q.* Do you know what patients those two nurses had to deal with in the Isolation Ward?—*A.* A case of scarlet—an empyema; it might have been scarlet.

*Q.* That was one case?—*A.* Yes.

*Q.* Were there any others?—*A.* No.

*Q.* On which night was that?—*A.* That was on the same night as it occurred in the Children's Ward.

*Q.* One night or two?—*A.* The same night and the next night.

*Q.* On the same two nights?—*A.* Yes.

### AT THIS POINT THE MATRON ENTERED THE ROOM.

The Commissioner: Shall I put the matter briefly to the Matron?

Mr. Procter: If you will, Sir.

The Commissioner: Well, Matron, the charge we are considering is this. "A nurse who had only four months' training was left in charge of the Children's Ward at night." What Dr. Shepherd tells us is that you had a probationer named Dann who was of about three or four months' training, that for the whole of one night and part of another she was in charge of the Children's Ward, which was then full, and she was in charge of the Children's Ward in spite of the fact that you might have put in charge of that ward either Nurse Thompson or Nurse Bewlay, more experienced nurses, who were then only occupied in looking after one patient in the Isolation Ward.

The Matron: I could not very well take a nurse from Isolation.

The Commissioner: Have I put the case correctly?

Mr. Baker: With perfect accuracy.

The Matron: To take a nurse from Isolation and put her in the Children's Ward.

The Commissioner: No, Matron. That you had available nurses of longer training than Nurse Dann whom you might, not for that particular night, to whom you might have arranged to give charge of the Children's Ward. Now what is your reply?

The Matron: My reply is that that occurred in the middle of the night, about two or three o'clock in the morning, that the nurse was not feeling well.

*Q.* The regular nurse?—*A.* The regular nurse was not feeling well, consequently it was deemed advisable she should go to bed, and someone else must take her place. Well, naturally, in the middle of the night, you don't wish to disturb two or three other wards. Nurse Dann had been a considerable time in the Children's Ward, knew every case, was particularly capable with them, and the calling of her up disturbed no other ward at all. There were fourteen children in the ward the night she was called up.

*Q.* And the beds are?—*A.* Twenty-four.

*Q.* Fourteen patients—twenty-four beds that night?—*A.* And they were mostly very convalescent cases that this nurse was quite able to attend to, having been in the ward so long. She probably knew more about the children than an older nurse who had not been in the ward.

*Q.* And how long did she remain in charge of the ward?—*A.* One night and a part of a night—the part of the night that she was called up and the following night, when I deemed it advisable to let the nurse remain in bed another night.

*Q.* During the time the proper nurse, if one may say so, was not very well?—*A.* Yes. The second night I think there were some cases came in. The night she was called up I know there were fourteen, and I think one or two came in the following day.

*Q.* You say that it would have been quite improper to have moved either of those nurses from the Isolation Ward. You mean by that that the character of the case they were nursing there would have rendered it improper to move them? I see that you have got other reasons.—*A.* At present I cannot really remember what case was in Isolation.

*Q.* What we are told is that it was empyema?—*A.* Oh, then it had scarlet as well.

*Q.* What Dr. Shepherd says is that it was probably connected with scarlet fever?—*A.* Then I should not consider it advisable to put her in the Children's Ward.

Mr. Procter: There were fourteen cases, and I understand they were none of them serious, I think you say?—*A.* They were very convalescent.

*Q.* And the four that might be described as the worst of the lot I have got a list of here. Can you remember them?—*A.* Yes.

*Q.* There was a case of Charlie Wynne, a case of typhoid. Do you remember that?—*A.* Yes.

The Commissioner: Was that a convalescent typhoid?—Yes.

Mr. Procter: We have the reports.

Mr. Baker: Yes; I think we can agree as to these—a case of typhoid, a case of pneumonia, a case of chorea, and a bottle-fed baby.

Mr. Procter: Yes, these were the four cases, and they were not serious cases.—No.

Mr. Procter: I have got a copy of the Night Report here. Would you like to have the particulars, sir?

The Commissioner: Well, I had better look at these particulars. Does anything arise as regards these cases? Is it contended that they were all of a convalescent sort?

Mr. Baker: Is that disputed?

The Commissioner: Is that disputed?

Dr. Shepherd: They were not all convalescent.

Mr. Baker: It is disputed.

The Commissioner: It is obvious even to a layman that a typhoid case which is in a serious condition may be different from a convalescent typhoid.

Mr. Baker: Well, I will ask Dr. Shepherd now.

The Commissioner: Now, may I just take the cases, and then we will ask Dr. Shepherd about them. One is said to be a case of typhoid—Charles Wynne. "Night report, that he slept very well. He took beef-tea, bread and milk, and was very comfortable." Have you anything to say as to whether that case was convalescent? What stage of typhoid was it, Dr. Shepherd?

Dr. Shepherd: It was about, I should say, the second week, going on for the third.

The Commissioner: Then typhoid, assuming the diagnosis to be accurate, is not convalescent in the second or third week?

Dr. Shepherd: No, it is not convalescent.

The Commissioner: Is there any note of the temperature?

The Secretary: It is on my list 97.4.

The Commissioner: Can you tell me the date of the admission of Charlie Wynne? When was he admitted?

The Matron: I don't remember at all.

The Commissioner: I don't see the chart. I only have a note, temperature 97.4, but obviously that is not the whole of the temperature chart. I can judge of the duration of the case either by the dates or the temperature chart, and I have neither.

The Secretary went to fetch the temperature charts.

### TEMPERATURE CHARTS SENT FOR.

The Commissioner: I don't know when the typhoid was admitted. I know that the time I am dealing with was January 13.

Mr. Baker: Perhaps while this information is being obtained I might ask the Matron one question. You said that you thought it better not to disturb two or three of the wards?—Yes.

*Q.* Dr. Shepherd's suggestion was, wasn't it, that you should, to use your own word, disturb one other ward, the isolation ward, where two nurses were looking after one case?—The Matron: Well, they would not be looking after it at once. One would be the day nurse, and one would be the night nurse.

*Q.* But I mean it would not involve disturbing more than that one ward, would it?—*A.* But I think it would be impossible for me to take a nurse who was nursing scarlet fever and put her in the children's ward. I should not think of doing such a thing.

*Q.* It would mean disinfection, of course, wouldn't it?

Is there any other reason to make it impossible?—*A.* Well, you would hardly begin in the middle of the night to disinfect a nurse and leave a patient alone. It is a long process. The nurse has to wash her head.

The Commissioner: Well, all of us who are married know that that is a long process.

There being some delay in the provision of the temperature charts, the Commissioner said: Shall we try this pneumonia case? There is a case of pneumonia and empyema—Lucy Ruston. The temperature there is 98. Do you dispute that that was convalescent?

Dr. Shepherd: I cannot remember all these cases now. I cannot remember what stage they were in.

The Commissioner: Your impression of the typhoid is that it was in the second or third week?

Dr. Shepherd: It may have been later on; that is so far as my recollection serves me. I was not well myself when the typhoid came in.

The Commissioner: And the empyema you are in doubt about? There appear to be two empyemas, Lucy Ruston and Alice Copley?

Dr. Shepherd: Lucy Ruston had been done last; I think some time about Christmas or a little after.

The Commissioner: There was a case of chorea, on which the note is "Slept very well, ordinary breakfast, very comfortable. Alfred Kendall." Do you remember?

Dr. Shepherd: Yes, he was restless.

The Commissioner: And the bottle-fed baby?

Dr. Shepherd: That child sometimes required attention all night. I have heard it cry all night. It had to be nursed.

The Commissioner: What was it admitted for? All I see is that it was a bottle-fed baby, which is hardly a disease.

Dr. Shepherd: I don't know; I think it was Dr. Macqueen's case.

Dr. Macqueen: It was Hampshire, I think.

The Commissioner: What was the matter with the bottle-fed baby?

Dr. Macqueen: It was osteomyelitis, I think.

At this point Nurse Dann was called for, but the Matron explained that she was ill in bed under the care of Dr. Evelyn.

Dr. Evelyn: If it is very exceptional, she probably might get down.

The Commissioner: If she is ill in bed and warded, I don't think it is of such importance.

Mr. Baker: It is, I think, admitted that she had only three or four months' training?

Mr. Procter: That is not disputed.

The Commissioner: What were you desirous of bringing Nurse Dann before us for? What was she to prove?

Mr. Procter: I thought she might prove who the baby was and what was the matter with it.

The Commissioner: You don't want her to come and say the ward was light and she found no difficulty in dealing with it.

Mr. Procter: Well, I would have asked her, if it had been possible. We have sent for the charts.

### SURGEON KEPT WAITING FOR PATIENT IN OPERATING THEATRE.

The Commissioner: Well, can we pass on? The next item is: "Delay (sometimes as much as two hours) in taking a patient to the theatre after instructions had been given for the case to be in readiness at a certain time."

Mr. Baker: There is an explanation I should like to give on that. There is a printer's error there. As it reads, it is "delay," then, in brackets, "sometimes as much as two hours." The suggestion was not intended to be at all that there was a delay of two hours, but that the delay was caused in bringing a patient to the theatre after there had been two hours' warning that that patient would be required at a certain time. It is unfortunate, I agree, but it is quite obvious that, as it stands, that is not intended.

The Commissioner: I think that is brought out in the note that appears in this note-book, No. Q of evidence taken at the nursing sub-committee of the Medical Board. "H. S. reports delay and unpunctuality in bringing cases to theatre, and quotes case of an emergency from Ward 6, in which Mr. Gostling was kept waiting in the theatre twenty minutes, notwithstanding that two hours' notice of the time of operation had been given. Says that similar delay has occurred often from all parts of the hospital except Ward 7, Sisters Hutchins and Barron, and the ten wards." Then there is a further note: "Delay in getting cases to theatre. Sister Barron, called, says delay in getting cases to theatre is apt to occur, owing to shortness of staff, when there is a succession of cases to go to the theatre from one ward."

Mr. Baker: I will ask Dr. Macqueen one question on that, whether that is his note. It purports, I think you say, to be Dr. Macqueen's note?

The Commissioner: It purports to be Dr. Macqueen's report given in evidence before this Committee.

Dr. Macqueen: Yes, it is. It referred principally to emergency cases, not on the ordinary operating days when there was a succession of cases.

The Commissioner: Have you particulars of cases in which it has occurred, or is it a general statement?

Dr. Macqueen: It is a general statement.

Mr. Baker: Is the case of Mr. Gostling one specific case, do you mean?

Dr. Macqueen: Yes.

Mr. Baker: Can you point to others?

Dr. Macqueen: It was the only specific case that I can refer to.

Mr. Baker: The only one to which you can refer now?

Dr. Macqueen: Yes.

The Commissioner: That is the only specific case given here. (To Mr. Procter): Do you know which case it refers to?

Mr. Procter: No, we do not, in such a vague charge as this.

The Commissioner: You mean the finding of the sub-committee on it. Unless you can find other things in this book—all I have been able to find is what I have read.

The Secretary: We did not report on that date. We inquired from various sisters, and we can speak to it.

The Commissioner: Can anybody tell us as to this particular case of Mr. Gostling?

The Secretary: We have no particulars. Nothing was given in this statement as to which case was referred to, and we have been really unable to find any specific instance. Therefore our note of that is that the statement is so vague and no particulars given we rather say we want proof.

The Commissioner: Well, Dr. Macqueen, can you give us the name of the patient?

Dr. Macqueen: I am afraid I cannot now. The delay occurred in more than that specific case. Practically in every case of emergency there was delay.

The Commissioner: We shall be quite content at the moment, I think, to have some details of this case which you reported in this book.

Dr. Macqueen: I am afraid I cannot remember it now. Mr. Gostling asked Dr. Shepherd first of all to go to the wards and see why there was delay. It was Ward 6.

The Commissioner: Do you think that Mr. Gostling might be able to help us with this case?

Dr. Macqueen: It is possible. The patient in this case came from Ward 6. He first of all sent Dr. Shepherd to see why they were so long, and then he asked me to go.

The Secretary at this point went to fetch Mr. Gostling.

Mr. Baker: The minute you read out, Sir, was that a decision of the Medical Board?

The Commissioner (passing the book): That was a sister's statement in evidence.

Mr. Procter: If that is the only case I may say it was inquired into and found that there was in that case a delay of twenty-five minutes—if that is sufficient.

Mr. Baker: Yes, Mr. Gostling's case.

Mr. Procter: But there is an explanation which we should like to put before you.

The Commissioner: Yes, Mr. Gostling will put it before us.

### MR. GOSTLING EXPLAINS.

The Commissioner: Now we have to take Mr. Gostling over two other cases; the first is the delay about getting persons into the operating room (No. 99 charge)—sometimes as much as two hours was quoted by Dr. Macqueen.

Mr. Baker: Do you ever remember delays in the operating theatre because patients were not brought in?—Yes.

*Q.* Could you remember cases?—*A.* I think it was one or two cases.

*Q.* Generally emergency cases?—*A.* I think it was entirely.

*Q.* Do you remember one occasion when you sent Dr. Shepherd or Dr. Macqueen into the ward to find out the cause of the delay?—*A.* Yes.

*Q.* I think there was one case of a very long delay of about twenty-five minutes?—*A.* Yes, I cannot remember exactly the time, but I was waiting in the theatre and it seemed a long time.

The Commissioner: Do you think the case reported occurred?—Yes, I feel sure it did.

*Q.* Do you know of other cases in which you sent one or other of the residents?—*A.* I do not recall another.

Mr. Procter: Was no explanation given you?—I do not think there was.

Mr. Baker: Certainly. I was dealing with another point. I was not sure whether this was in evidence, because I think it may help to shorten the proceedings. I understand that is evidence given before the Medical Board Committee who sat to inquire.

The Commissioner: By Sister Barron. No, Mr. Baker, the sister says "the delay in getting the cases to the theatre is apt to occur owing to shortness of staff when there is a succession of cases to go to the theatre from one ward." What she means is this, that supposing a surgeon arranges three or four operations one after another, of his own patients, a patient is taken to the theatre. With that patient I believe it is the custom here for a sister and a nurse to go to the theatre, so that that takes a sister and nurse out of the ward. That case is operated on. The next case, before coming to the theatre, may require a cathedra dose before it can follow case No. 1, and some delay occurs in that manner. But that is not Dr. Macqueen's statement. His statement is that delay occurs in emergency cases. There is a distinction between the two.

Mr. Baker: What I was wondering was whether there is a finding by that sub-committee.

The Commissioner: No, that is the statement of Sister Barron.

### GENERAL LAXITY IN CARRYING OUT ORDERS.

In order to avoid delay, the Commissioner proceeded: Do you think we might go on to No. 10? Have we any further reference to No. 10? Would you give us evidence of No. 10, Mr. Baker? It is "General laxity in carrying out orders."

Mr. Baker: This is Dr. Shepherd. I think you both speak to this. Dr. Shepherd can give you evidence upon this.

Mr. Baker: Laxity in carrying out orders by whom?

Dr. Shepherd: By anybody who received them, either sister or nurse.

The Commissioner: Laxity on the part of sisters or nurses in carrying out the orders given by the residents?—Yes.

Mr. Baker: Have you in your mind any particular ward?—I had Ward 6.

The Commissioner: Sister ——? Sister Hill. And the Children's Ward when the late Sister Victoria was there. Those are the two that I had in my mind.

Mr. Baker: Any other wards?—Besides the one specific case that has been dealt with, when my orders were not carried out.

The Commissioner: Which case?—Collier.

The Commissioner: Is Sister Hill still here?

The Secretary: No.

The Commissioner: Both these sisters have left, have they?

The Secretary: Yes.

Mr. Baker: Was there another instance which you point to, Dr. Shepherd; the instance of washing?—*A.* Yes, I point to that as well.

The Commissioner: That is a case we have had before.

Mr. Baker: Are there any other cases that you can call to mind at the moment as examples?—Not that I can call to mind at the moment. These things are rather difficult to get at.

Mr. Watson: You have no specific allegations with regard to any cases except the cases we are dealing with in The Hospital paper?—Yes.

The Commissioner: I understand that you would have been prepared to give us an example in the case of Sister Hill?—Well, I did refer to the matter of going round at night, or afternoon visit, ordering things for certain patients, and, on inquiring as to the result, finding that the whole of it had been omitted to be given out. That is the general statement.

Mr. Baker: Perhaps I ought to have asked you this in regard to the washing case. What is the particular charge you allude to in the washing case?—*A.* That my orders were not carried out there. I said that a patient had to go to bed and be left quiet.

*Q.* I think it is already in evidence that that order was never received in the ward where the patient was?—*A.*—No; it was never carried out. It never seemed to have been received.

Mr. Procter: In the case of Sister Hill you are speaking generally?

Mr. Watson: It was denied that it was ever given.

Mr. Baker: I don't think it could be denied that it was ever given. It has never been denied.

Mr. Watson: A nurse said they were the only people who attended there, and they never heard the order.

Mr. Baker: The only evidence that was called, apart from my evidence, was evidence by the sisters or nurses in the ward where the patient was received, not in the ward where Dr. Shepherd gave the order at all.

Mr. Procter: Yes, I agree there.

The Commissioner: My recollection is the recollection of Mr. Baker.

Mr. Procter: You have made a sort of general charge against Sister Hill, Ward 6, of not carrying out orders?

Dr. Shepherd: Yes.

*Q.* Did you ever report any of these incidents to the honoraries in charge of the cases?—*A.* Not to my recollection. I may have complained about Sister Hill to certain of the honoraries.

*Q.* Did you mention any specific instance where your orders had not been carried out?—*A.* Not to my mind at present.

The Commissioner: Did you ever report the negligence of Sister Hill in not carrying out orders to the Matron?—No, never.

*Q.* That would have been the more ordinary course, I suppose, wouldn't it?—*A.* It might have been.

*Q.* It is usual?—*A.* It is usual, yes, I quite agree.

*Q.* I mean—I understand your care in the witness-box, so to speak—but it surely would be the custom to report a sister to the Matron?—*A.* Yes.

The Commissioner (to Mr. Procter): You cannot carry that further, I take it. You have no witnesses to summon.

Mr. Procter: I cannot.

### NEGLECT TO TAKE PULSE RESPIRATIONS AND IN REPORTING OF DEATHS.

The Commissioner : No. 11. "Neglect to take pulse and respirations, already referred to in the Children's Ward, but also found in other wards." Well, now, the Children's Ward, I think, we have dealt with.

Mr. Procter : We have dealt with it.

The Commissioner : But are there any particulars of other wards? Who is responsible?

Mr. Baker : Dr. Shepherd. You are dealing with Ward 2.

The Commissioner : I am dealing with any ward that is not the Children's Ward.

Mr. Baker : Can you, Dr. Shepherd, give us any specific instances?—Oh, it is Dr. Macqueen.

Dr. Macqueen : On a good many of the charts in Ward 2 we found, for quite a considerable period, no record of pulse or respirations.

Mr. Procter : That affects Sister Hannah. Might we have her in?

The Commissioner : Especially if she would come with the charts. Do you keep any of the charts?

The Secretary : Oh, yes, the charts are kept; but we do not know which wards.

The Commissioner : I sympathise with you.

The Secretary : There may be delay.

The Commissioner : But you probably have some charts, and if you could produce some—between which period, Dr. Macqueen? I want to see some samples of charts.

Dr. Macqueen : It would be prior to Christmas.

The Commissioner : Would you be content with the charts for the month of December?

Dr. Macqueen : Yes.

The December charts were then sent for.

Dr. Macqueen : Perhaps the sister will admit that she did not keep the charts. (Laughter.)

### SISTER HANNAH SENT FOR.

The Commissioner : I think we may as well go on with No. 12 : "Frequent failure to report deaths to the Resident in charge."

Mr. Baker : They both speak to this. Dr. Shepherd, have you any specific instances to give to the Commissioner in support of that statement?

Dr. Shepherd : Yes, there is the old case of Collier.

Mr. Baker : Any others?—Yes, a child, or rather a baby, of—I think it would be about ten days old; I cannot exactly recollect. In Victoria Ward.

The Commissioner : In the days of the later sister?—No.

*Q.* Later?—*A.* Later.

Mr. Baker : Any others?—The death occurred during the night—any time after half-past eight o'clock and eight the next morning.

*Q.* Were you on duty that night?—*A.* I am always on duty.

The Commissioner : What was the case?—A case of morbus maculosus.

*Q.* And when was the death reported to you?—*A.* The first I heard of the death was from Dr. Macqueen in the corridor that morning at eleven o'clock.

Mr. Baker : Was it your case, or Dr. Macqueen's?—It was my case.

*Q.* Do you know when the child died?—*A.* I think it died some time between five and seven in the morning, but of that I cannot be certain.

The Commissioner : Is there a sister concerned?—It would be the night sister.

*Q.* It would be, presumably, the night sister who was looking after the Victoria Ward.

Mr. Procter : Can you give us the date?—I cannot; but I will get the date for you if you will give me the counterfoils of the death certificate book.

The Commissioner : Yes, that will be an easy plan.

Mr. Gayner : I will get the death certificates.

Dr. Macqueen : There is a particular instance—I can also get the date from the death certificate. It was a case of a man in Ward 2, who was operated on. At the operation it was found that he had malignant disease of the pancreas. I saw him about seven in the evening, and I asked that his friends should be sent for as he was getting rapidly worse. The next I heard of the case was the friends came and asked me for the death certificate.

The Commissioner : You had mentioned that case?

Dr. Macqueen : Yes.

Mr. Baker : Not before you in this inquiry?

The Commissioner : No, but it is on his notebook.

Mr. Procter : When did they ask you for his death certificate?

Dr. Macqueen : I should say about 8.30, an hour or an hour and a half after I had seen him.

### SYSTEM OF REPORTING DEATHS.

The Commissioner : Was there any system in the hospital of reporting deaths? Are there any tickets to be left with the Residents?—It was customary for the Night Sister, if the death occurred during the night, to leave a note on the Resident's breakfast table to the effect that such-and-such had died.

*Q.* That was the way in which you would have expected to hear of the death?—*A.* Not of the one to which I refer, but of the one to which Dr. Shepherd refers. The one to which I refer occurred in the evening.

The Commissioner : Perhaps this represents the practice : "When deaths occur in the daytime, the Resident is informed verbally if possible. If, however, circumstances prevent this, a slip of paper is placed in their room. At the same time a note is sent to the Matron, and this is the invariable rule." Does that represent the rule of the place?—Yes.

The Commissioner : We might now, if possible, see Sister Hill with regard to this charting.

Mr. Procter : She has gone.

The Commissioner : Oh, it is not Sister Hill. I want the Sister of Ward 2.

Sister Hannah then attended. At the same time Dr. Macqueen said he had found among the counterfoils of the death certificates the date of the case to which he referred.

### SISTER HANNAH EXAMINED.

The Commissioner : I am afraid you will have to wait. We have now got a witness. Now, Dr. Macqueen, this is the Sister of Ward 2, and your statement is that charts in that ward were found defective. It is not that they were charted incorrectly, but they were not charted at all. Now, Sister, is that so, or is it not?

Sister Hannah : To my knowledge, I thought I was doing them properly.

*Q.* Well, do you mean that you were charting?—*A.* That I was charting them.

*Q.* Understand, Dr. Macqueen does not say that you chart a temperature of 101 as 100, but his statement is that it has happened in your ward that the cases are not charted at all?

Dr. Macqueen : In pulse and respirations, not in temperature.

Sister Hannah : But, Dr. Macqueen, why didn't you tell me?

The Commissioner : That, I think, was a very natural impulse for you to say that, but it is not exactly what we want. We want to know whether as a fact that happened?—I don't know that it did. I don't know of any case. Dr. Macqueen never mentioned one case to me that he was dissatisfied about in my ward during the time that he was house surgeon.

*Q.* It is not a particular case, but it happens that pulse and respirations were not charted.—*A.* I don't know of one. I have tried to keep them right.

The Commissioner : Then, Dr. Macqueen, your rather optimistic view is not realised?

Sister Hannah : I have tried to keep my charting right, and I was never told of any fault.

Mr. Baker : Cannot we see from the charts themselves?

The Commissioner : If I spend my time in looking through these, obviously there will be delay. Perhaps Dr. Macqueen will look at them.

A pile of charts was passed to Dr. Macqueen, who picked one out and it was shown to Sister Hannah.

The Commissioner : There is a case in point. This was Reuben Sprutson, under Mr. Gostling. He came in on April 13. His age was 68. There is no notice of what the disease was, but his temperature is charted. His pulse and respiration appear not to be charted there.—It was an ordinary case. First of all I took his pulse, and I thought it was not necessary to go on taking it.

Mr. Baker : That is one case. There (passing over the charts) are three others.

The Commissioner : This is a case of John Petty. That was a case of senile gangrene, in which amputation was performed. The temperature runs continuously charted. Pulse and respiration are taken occasionally. And your explanation of that, sister, would be?—I was away when this paper was entered up.

*Q.* But your explanation at the time you were there would be, I suppose, that the case was going on satisfactorily?—*A.* Yes, probably I should take his pulse but not record it.

*Q.* This is another one in which the pulse and respiration were not taken—I will say were not recorded—for about a fortnight?—*A.* I was away when this patient was entered up.

*Q.* That would prove that what happened when you were there happened when you were away.

Dr. Macqueen : This is one in which the patient was admitted on March 5 last year.

The Commissioner : Suffering from what?

Dr. Macqueen : It does not say.

*Q.* I thought perhaps you remembered the case?—*A.* No, I don't. Discharged on the 7th ———.

*Q.* Been in more than a month?—*A.* Almost two months, and in that time it has only been charted twice—the day of admission and the day on which the patient had an anæsthetic.

*Q.* But you have no recollection of what the case was?—*A.* No; as a matter of fact it was before I came.

*Q.* Were you in charge then in May?—*A.* Yes.

Sister Hannah : It was a very old case, which had been in the ward before.

Mr. Baker : I don't want to multiply instances of this, but if we could take it that perhaps Dr. Macqueen's optimism was not so misplaced after all, now that the nurse is confronted with these charts.

### PULSE AND RESPIRATION CHARTED WHEN SISTER CONSIDERED DESIRABLE.

The Commissioner : I think, sister, it comes to this, that the pulse and respiration is charted by you when it appears to you desirable to do so?—Yes, sir.

*Q.* And that sometimes you have taken them with your fingers on going round, without recording them?—*A.* Yes.

Mr. Procter : Does it not appear on those charts where the pulse and respiration is not taken the temperature is normal?—Yes, where the temperature is above 99 the pulse and respiration is always taken and reported.

*Q.* But where the temperature is normal I gather you do not take pulse and respiration?—*A.* No, not in all cases.

*Q.* And is that the reason?—*A.* Yes.

The Commissioner (to Dr. Macqueen) : Had you ever called sister's attention to this?

Dr. Macqueen : Not Sister Hannah's attention.

*Q.* Had your honorary staff ever raised objections to this absence of charting?—*A.* No. Of course, we were more particularly referring to the Children's Ward.

The Commissioner : Yes, that was in the days of the sister who has gone. Now we want to identify the baby and the patient who suffered from the disease of the pancreas.

Mr. Procter (referring to papers brought into the room) : This is case No. 8.

The Commissioner : The question here is which is the typhoid patient. Charles Wynne? He was admitted on the 18th of December. What is the date on which this nurse had been in charge a day and part of a night?

Dr. Macqueen : The night porter would tell you that.

Mr. Procter : January 13, I think, Sir. "Called up between 2 and 3 A.M. on January 13 to relieve the night nurse of Victoria Ward." Is that right, Matron?

The Matron : Yes.

The Commissioner : Then Charles Wynne at that time, January 13, had temperature at night of something under 99. It was normal next morning; 99 on the 15th; and the temperature runs very much in a normal fashion there. So that if that was the typhoid it would come very much down to the normal?

Dr. Shepherd : Yes.

The Commissioner : Now the next one : Lucy Ruston. (The paper relating to this case could not be found, however, but that about Alice Copley was.) What was Alice Copley suffering from?

Dr. Macdonald : Empyema.

Dr. Shepherd : Alice Copley was convalescent.

The Commissioner : She had been admitted on October 10. I see she must have been convalescent.

Dr. Shepherd : That is not the one that is referred to.

The Matron : It was mentioned as the empyema.

Mr. Baker : No, but Dr. Shepherd says there were two cases.

The Matron : Lucy Ruston was empyema and pneumonia.

The Commissioner : There is only one case that we really want to trace, then.

Dr. Shepherd : Lucy Ruston.

### DEATH CERTIFICATES EXAMINED.

The Commissioner : The chorea is immaterial for your purpose—I mean temperature is immaterial. That dislocates the proceedings once more. Now I think we may as well have those certificates.

Mr. Baker : Yes.

Dr. Shepherd : The child with morbus maculosus, Alice Halstrip, died on January 3.

The Commissioner : When?

Dr. Shepherd : The time is not recorded on the counterfoil.

*Q.* That was the case that the night sister should have reported to you?—*A.* Yes, or I should have had a note left on the table.

*Q.* The night sister was implicated in that case?—*A.* Yes.

*Q.* And it was January 3, so we know who the night sister was?—*A.* Yes.

*Q.* Are you satisfied who the night sister would be?—*A.* I am not absolutely satisfied about dates. I think it will be Sister Joicey.

Mr. Baker : The other one is a patient with malignant pancreas disease, named Fothergill, aged 38, who died on December 28.

The Commissioner : Is that also the night sister?

Dr. Macqueen : No, I think it was the day sister. At any rate it occurred in the evening. It was either just at the end of the daytime or the beginning of the night.

The Commissioner : I hear that Mr. Gostling cannot possibly come just now. He says he does not remember anything about the matter. He can come before six, so you will get him as soon as you

Dr. Macdonald : He did say something to us, I think. I think there is something in one of those books about the delay.

Mr. Procter : All we wanted in that case was to have offered to you some explanation of the delay, which we can probably get from some of the sisters concerned.

The Commissioner : I thought the best person to ask was Mr. Gostling.

Mr. Procter : If we can get at him.

The Commissioner : He says he can come, but he says he does not remember anything about the matter. Do you want to present evidence under those circumstances?

Mr. Procter : Yes, we will make inquiries, if you will let it stand over.

Mr. Baker : I understand this note that there is could not be made use of as documentary evidence.

Dr. Macdonald : I thought there was a note in those two books that we sent to you about the time of the delay that Mr. Gostling had. It is indexed at the end.

Dr. Macqueen : I may point out that the report of January 3, on which the death occurred, is initialled "E. H." I suppose that was Sister Hutchins.

The Commissioner (to Sister Hannah) : It appears that on December 28 a man named Fothergill died in your ward. He was suffering from malignant disease of the pancreas. I don't know whether you remember the case. This may refresh your memory (handing counterfoil). He was thirty-eight.

Dr. Macqueen : It was a case that came from Ward 3, and the patient had an operation performed on him by Mr. Gostling.

Sister Hannah : And I did not report the death to you?

The Commissioner : Oh, no, no. Do you remember the case?—I remember the case.

*Q.* That is the first question. You have now got on the track of the case, anyhow. I sympathise with you very much, because of course to have a case suddenly presented to you, when you have got a large number of cases under your care, is difficult.—*A.* It is difficult. I was only in the ward one day.

*Q.* The other statement is that the death of this patient was not reported by you to Dr. Macqueen, and that the first he heard of it—though that does not concern you—the first way in which he knew the patient had died was by seeing the relatives come to the hospital, and he learnt of the death from them, before you had told him or informed him in any sort of way that the patient had gone. —*A.* I cannot remember the facts about it, but I am perfectly sure that I should not do it. The first thing I think of doing is going to report a change in the patient's condition.

*Q.* This patient was dead?—*A.* Yes, but then there was a certain change.

*Q.* That is an important change, isn't it?—*A.* I remember the patient. Dr. Macqueen came up to see the patient.

Dr. Macqueen : When I saw the patient he was alive.

Sister Hannah : Didn't you see him after death?

Dr. Macqueen : After the friends came.

Sister Hannah : I am perfectly certain you would have a report of that case given to you by a nurse.

Mr. Baker : You "would have" or "should"?—I cannot say that he did, but the first thing I think of doing after death is sending a nurse with a message to the doctor.

*Q.* Whom would you send?—*A.* One of the nurses from the ward.

*Q.* You don't know, in this particular case, who was the nurse you sent?—*A.* I cannot remember in every case which nurse I sent. I know quite well Dr. Macqueen knew the patient was dying. He came in and ordered strychnine for him, and he was very near the end then.

*Q.* Yes, but that does not touch the case, sister.—*A.* I cannot remember the nurse who went.

The Commissioner : Well, I don't think we can carry that any further.

Mr. Procter : I am afraid not.

The Secretary : If we had had notice we might have proved it. This is the first we have heard of it.

### COUNSEL'S PROTEST.

Mr. Baker : I don't think that is a proper remark to make. I have given you a certain amount of laxity, and have not complained of several remarks you have made. It is a matter for comment, and I don't think it ought to go on the notes.

The Commissioner : I don't think in this case Mr. Neden has suffered by the fact that he had not notice. Of course, we all know that it is difficult to meet a charge hastily, but in this case nothing that would have been done would have furthered the matter. Here we have the sister, who tells us she is quite clear that she must have told the nurse to give the information, but she cannot remember the name of the nurse. We could not have carried it further, I think, with six months' notice. Now we come to January 3, Alice Halstrip. This is the small baby, and there you think that Sister Hutchins was concerned, because she initialled the night report. And you are getting Sister Drysdale as well, aren't you?

Mr. Procter : No, Sir.

The Secretary : I met Sister Hutchins just outside the door, and asked her if she remembered the case of Halstrip, and she said "No."

Dr. Macqueen called attention to a note in the night report book : "Baby Halstrip seen dead, 7.20." Signed by Sister Hutchins.

The Commissioner : She would have left that note before she went off duty. What time did the new sister come on duty?

The Matron : Eight o'clock in the morning.

The Commissioner : So that if the night sister saw a child dead at 7.20 A.M. it would be her business to report it

The Matron : Yes.

### SISTER HUTCHINS CALLED.

The Commissioner : There is yet one other matter we have to inquire into. It seems that on the morning of January 3 a small child died. It was only about ten days old, and it died at that time. Have you any recollection of that?

Mr. Baker : I am told it was three weeks old.

Dr. Shepherd : The child's cot was in the middle of the room.

The Commissioner : You said ten days.

Dr. Shepherd : I said ten days.

The Commissioner : It was three weeks old, and I presume it was rather a blotchy looking child. It was suffering from morbus maculosus. Do you remember it?

Sister Hutchins : I think I do, but really two children died close together. In fact, I think both died the same day.

*Q.* Let me put it to you that you seem to have had something to do with it, as you say there that it died at 7.20 A.M., and it was seen dead.—*A.* Yes.

*Q.* And I suppose you must have seen it, didn't you? —*A.* Yes.

*Q.* Well, now, the statement by Dr. Shepherd is that he was not informed of that death. The custom is to put a note in the Resident's room, isn't it, for night deaths? —*A.* Well, since Collier's affair that is. I have been most particular about reporting deaths to the Resident. I think I would have gone to Dr. Shepherd's and told him that that child was dead.

*Q.* Between 7.20 and the time——?—*A.* As soon as the child was dead.

*Q.* What a very early caller you would have been. Do you find your Residents generally active at 7.20 A.M.?—*A.* Sometimes they are.

*Q.* And you would have called on him to tell him the baby was dead at that hour, would you?—*A.* I think I would have told him about the child.

*Q.* I feel sure if the child had been alive, and he could have done anything for it, you would have called on him, but simply to report a death would you have thought it necessary to wake him at 7.20, or before 8 o'clock? As a matter of fact, I think your memory is defective as to whether you did anything?—*A.* I cannot remember whether I went to his room or whether I left a note, but I think I would be most particular.

*Q.* You have no recollection?—*A.* I cannot remember.

### MORE CARE SINCE MORTUARY INCIDENT.

*Q.* Then I think you say that things have been a little more regular in that matter since the incident of Collier?—*A.* We have been most particular.

*Q.* Before then a little more laxity was admissible?—*A.* Well, I have been most careful.

*Q.* You really cannot tell us that you did go to Dr. Shepherd's room?—*A.* I ought to have gone to Dr. Shepherd's room, but at 7.20 I don't suppose I would.

*Q.* Or to have left a note?—*A.* Well, if I didn't go to his room I must have left a note.

*Q.* Where was the note generally put?—*A.* On the dining-room table. That is where they had their meal.

*Q.* So that it was more or less at the mercy of the housemaid or the lady who cleans the rooms?—*A.* Yes, I suppose it would be in that case.

*Q.* I am only suggesting possibilities.—*A.* Yes.

Mr. Baker: Can we finish off the empyema?

Mr. Procter: There is a chart here of Lucy Ruston.

The Commissioner: Is that the empyema referred to, Dr. Shepherd?

Dr. Shepherd: That probably is.

Mr. Procter: That was the pneumonia and empyema.

The Commissioner: Yes, that is the case Lucy Ruston. This is not the whole of the chart.

Dr. Macdonald: Apparently not. The part that refers to the admission he has neglected to bring. But this is evidently January.

The Commissioner: Then this has been in since December 18, Dr. Shepherd, according to this chart. Was it operated on?

Dr. Shepherd: That is not a case I recollect, if it was entered on December 18.

The Commissioner: I have only got a fragment of it here, and all it says is "Lucy Ruston, four years old." There is no diagnosis. It was admitted on December 18, and this is a piece of chart, I think, clearly, of January, or, at any rate, of a following month, because the chart begins on the 8th of the month, and she was admitted on the 18th.

Dr. Shepherd: Possibly that may be the child. I know there was one that had pneumonia, and developed empyema afterwards.

The Commissioner: Well, we may get the rest of the chart, I suppose, any time.

Dr. Macdonald: I will tell Mr. Neden when he comes.

Dr. Shepherd (after seeing the chart): Yes, that is the child.

The Commissioner: Well, then, what do you say as to the temperature?

Dr. Shepherd: The temperature was all right, but its pulse was exceedingly bad. On the 13th it was 120, 126, 120, and 128.

*Q.* And the child was how old?—*A.* Four years. Respirations 32, 38, 38.

*Q.* So that the child had been operated on, I suppose?—*A.* Yes, it had. I think it was operated on either on Christmas Day or the day after.

*Q.* Did the child ever recover, do you remember?—*A.* The child was in the ward when I left.

Mr. Baker: I see on December 14 the record says that it had 152 pulse.

The Commissioner: Oh, what Dr. Shepherd said was right.

Mr. Procter: The Matron says the child did recover.

The Commissioner: There seems no doubt that the child was in a grave condition, from that report.

Mr. Baker: We need not bother further.

The Commissioner: No, the child was in a serious condition.

### ABSENCE OF NURSES FROM WARDS.

The Commissioner: Now we come to 13:—

"It was a frequent occurrence to pay a ward visit and never see a sister or nurse until the visit was almost finished—sometimes not at all."

And then among the notes in the first volume is this entry—no, that is not the evidence of these doctors.

Mr. Watson: That is the only evidence we have.

The Commissioner: Then I am afraid I must ask you.

Mr. Baker: Dr. Shepherd, first of all, when you were in the habit of going through the wards—do you wish to mention any particular ward?

Dr. Shepherd: Ward 6.

*Q.* Ward 6 alone, or only particularly Ward 6?—*A.* Particularly Ward 6.

*Q.* Will you explain to the Commissioner what you used to find, and what you here complain of?

The Commissioner: Let us get the Sister here.

Mr. Procter: She has gone. She is not in the service.

Mr. Baker: Who is the Sister?—Sister Hill.

*Q.* Was Sister Hill always in charge of Ward 6?—*A.* Yes, always.

The Commissioner: Well, I am afraid we must take that as unfortunate, because we cannot get Sister Hill's attendance, but still——. It was a frequent occurrence to go through the wards and see nobody, or to see the sister or some of the nurses while you were at your last patient or your second last patient.

The Commissioner: After being how long in the ward?—Time enough to make your whole visit of the whole of the patients there.

*Q.* Yes, and that would be?—*A.* Five minutes, I should say.

*Q.* You mean you would have visited all the ward in five minutes?—*A.* Yes, if there were no serious cases; if you were walking round.

*Q.* What I am driving at is how long were they left without the attendance of a nurse? If you had said to me, "I frequently went round the ward and saw all my cases and saw no nurse," I should not have immediately concluded that that was only five minutes.—*A.* No.

Mr. Baker: Do you mean that without summoning a nurse you found nobody there?—Yes.

*Q.* Are you suggesting here or not that when you summoned the nurses they were not there?—*A.* I have——

The Commissioner: Oh, that is on another point. I dare say he is going to say that. What I was trying to find out was how long was he in a ward before a nurse came unsummoned, and he tells me he might go round the ward in five minutes. If that is the maximum time he went without a nurse, I can imagine that that is not what he is going to tell me exactly.

Dr. Shepherd: Five to ten minutes. Five minutes is a good average.

The Commissioner: Well, now, Mr. Baker, raise your point now. When he summoned a nurse did she come?

Mr. Baker: It may be my wrong suggestion. Do you suggest that when you summoned the nurses under these conditions they did not answer?

Dr. Shepherd: On certain occasions the patients at the end of a ward would shout for a nurse and yet nobody would appear.

*Q.* But I am not dealing with patients. I mean when you had to shout for a nurse. Have you ever had to summon a nurse and she did not come?—*A.* I never did so, for the simple reason that the patients used to do it for me.

Mr. Baker: I see.

The Commissioner: When the patients called to summon spirits from the vasty deep they answered, did they?—Sometimes there was an interval between; not always.

*Q.* You mean there were calls for a nurse and no nurse arrived at all?—*A.* Yes.

*Q.* Were there intervals in the day when the ward was left entirely without a nurse?—*A.* I cannot speak to that. I am not always in the ward.

*Q.* And when you did not get a nurse did you go in search of her yourself, or go out?—*A.* No, I always stayed in the ward until the nurse came.

The Commissioner: We have no direct evidence.

Mr. Baker: No; I am going to ask Dr. Macqueen if he will corroborate that with respect to other wards.

### DR. MACQUEEN'S EVIDENCE.

Mr. Baker: Have you heard the evidence given by Dr. Shepherd?

Dr. Macqueen: I have.

*Q.* Do you corroborate that?—*A.* I can corroborate it for the same ward. I was in charge of that ward prior to Dr. Shepherd having it.

*Q.* That is Ward 6?—*A.* Yes, and Sister Hill. On several occasions I have gone round the ward, examined fully at least three cases; I have even gone to the door of what you call the ward kitchen and knocked there, and called for a nursing sister, and no one has come. On several occasions I did the whole ward and the balcony as well, and left the ward without seeing anybody.

*Q.* And how many minutes do you put down for a complete round?—*A.* Sometimes as much as 25 minutes.

*Q.* So that your statement is that you were left unattended, or unseen, for 25 minutes on some occasions?

Mr. Watson: Did this happen often?—Not the 25 minutes.

*Q.* Did you complain to anybody?—*A.* I have asked the Sister where she had been.

*Q.* Only Sister Hill?—*A.* No, I have complained to Dr. Evelyn.

Mr. Baker: Have you been dealing only with Ward VI.?—Yes, up to the present.

The Commissioner: I understand up to the present all this evidence is Ward 6.

Mr. Baker: Have you noticed the same thing in any other wards?—I have noticed the same thing in Ward 2; not to the same extent.

The Commissioner: And that ward—is the Sister still here?—Sister Hannah. I have never gone round the whole ward without seeing anyone.

*Q.* What limit of time shall we put down there?—*A.* Say when I had examined my first two or three cases.

*Q.* Shall we say ten?—*A.* Not so much as that. About five.

*Q.* And at the end of that time have you taken any steps to secure the presence of a nurse or a sister, or they appeared themselves?—*A.* They have come.

Mr. Procter: Dr. Macqueen, did you ever report this to the Matron?—I don't remember whether I reported Sister Hill to the Matron or not.

The Commissioner: But you have not reported Sister Hannah?—No.

Mr. Procter: The case where you say you were twenty-five minutes on one occasion; that is serious; don't you think?—Yes.

*Q.* Did you report that to the Matron?—*A.* No, not to the Matron.

*Q.* Did you report it to anybody?—*A.* I told Dr. Evelyn.

*Q.* You reported that particular case to Dr. Evelyn?—*A.* I was talking so frequently about that particular ward to Dr. Evelyn I don't remember.

Mr. Procter (to Dr. Shepherd): Dr. Shepherd, have you ever reported these cases to the Matron?

Dr. Shepherd: I don't know that I reported them in detail, but I made frequent complaints to Dr. Evelyn about the ward.

*Q.* Have you any notes of the reports you made?—*A.* No.

*Q.* They were all verbal?—*A.* All verbal.

*Q.* And no note kept?—*A.* No note kept.

### DR. EVELYN CALLED.

The Commissioner: Perhaps we might take Dr. Evelyn. Dr. Evelyn, would you just come and tell us about this. What do you know about this matter?

Dr. Evelyn: Certainly, Sir. It is the first that I have heard of any complaints in regard to irregularity as to attendance of a sister or nurse in the ward—a complaint made by either Dr. Macqueen or Mr. Shepherd. They have on occasions made other complaints about Sister Hill, which I have investigated, but I do not remember their having made any complaint to me about coming into the ward and not finding the sister or nurse to attend. If they had done so—certainly on frequent occasions—I should have brought the matter before the notice of the Matron, as I did the other day, when I wished to make complaints of Sister Hill in regard to other matters.

Mr. Procter: I suppose if you heard of a case extending to twenty-five minutes it would impress itself on your memory very considerably?—Very considerably indeed.

*Q.* That would be a serious case?—*A.* Yes.

*Q.* And you are quite sure that was never reported?—*A.* Never.

Mr. Baker: Dr. Evelyn, you used the expression "I don't remember ever to have heard complaints." Are you prepared to say that neither Dr. Shepherd nor Dr. Macqueen ever made complaints to you about this behaviour?—I don't remember.

*Q.* Are you prepared to say that they have never made any complaints?—*A.* Yes, I think so.

*Q.* You are prepared to say that?—*A.* Yes.

*Q.* That they have never made complaints to you?—*A.* I don't remember any about this particular matter of not finding a sister or nurse in the ward.

*Q.* I put it to you that you had on one occasion a considerable conversation about this twenty-five minutes. Do you suggest, I mean, that they are deliberately lying? *A.* Not at all. I said this, that I do not remember their ever making any complaints to me on this question.

*Q.* That is a very fair answer, that you don't remember. But I want to know whether you go further than that, and say they did not in fact, and are prepared to tell the Commissioner that that is untrue?—*A.* The very fact that I do not remember it implies that they never did, as far as I can see. I should not say they had not reported these cases to me if they had.

*Q.* On the other hand, if you did not remember it you would say so?—*A.* Yes, I don't remember.

*Q.* Do you think it is possible it has slipped your memory?—*A.* I don't think so.

*Q.* You don't think it has, so that your suggestion is that they have invented it?—*A.* My answer is, I don't remember it, and I confess this, that if they had said it I should have remembered it, because I was always very careful about any complaints that were made to me about sisters or nurses.

*Q.* May I take it you won't put it any higher than that you don't remember it?—*A.* I don't remember it.

Mr. Procter: I can call before you Sister Hannah, who is sister of one of the wards.

The Commissioner: Yes, I should like to have her.

### SISTER HANNAH CALLED.

The Commissioner: We have heard from Dr. Macqueen that when he has been going round your ward, on several occasions he has found neither you nor any nurse there, and that he has had to wait as long as five minutes before anybody has appeared on the scene. Is that so?

Sister Hannah: It has occasionally happened sometimes so. We have been late with the ward work, and we have been having lunch in the day room. Perhaps that very same morning the doctor has come to the ward rather earlier than usual, and we have all been in the day room at lunch. The ward maid would be in the ward doing the work. She would come to the day room to tell us, but it would only be a matter of just coming over the landing, and I should go at once. That is not often.

*Q.* At a certain hour, when you and the nurses are having lunch, the ward may be left with nobody but a ward maid in it?—*A.* Yes, if the patients are all satisfactory, and they can be left.

*Q.* Yes, of course, you may have an extra, or special, or something of that kind in the ward?—*A.* Yes, and if there is any patient very ill one always stays in the ward and gets lunch later.

*Q.* The staff of your ward is yourself and how many nurses?—*A.* Three nurses.

*Q.* And you all go away to luncheon at the same time, unless there is some serious case in the ward demanding one or other to stay behind?—*A.* Yes, that is just a few yards from the ward.

*Q.* Yes, I understand that. Then during that time there is a ward maid, who is working in the ward, and who may, I suppose, be busy in the annexes scrubbing up something or doing some work?—*A.* She is usually polishing the floor at that time when we are having lunch.

*Q.* But her duties may take her into apartments which are not precisely in the ward, though just off the ward?—*A.* Yes.

Q. And it would be possible on such an occasion for the ward maid not to know of the presence of the resident in the ward, unless she had sharp ears?—A. Yes.

Q. Some residents are more light-footed than others, are they not, just as some night sisters are more light-footed than others?—A. Yes.

Q. That also is a fact well known to the profession, the younger members of it? We have all been young at one time.—A. Yes.

Mr. Procter : Will you explain, Sister, what you mean by lunch, exactly?—The lunch is just a cup of tea and bread and butter.

Q. How long does it take?—A. About a quarter to ten, or half-past nine sometimes.

Q. How long does it take?—A. About a quarter of an hour.

Q. Where do you take it?—A. In the day room, which is quite close to the ward. We usually hear the doctor when he comes out.

Q. You can hear what goes on, I suppose?—A. With the doors open. We can hear them talking. That has happened occasionally in the ward, and I have apologised to Dr. Macqueen. He has always been quite nice about it whenever it has happened. He has not complained to me of it.

Q. Would there always be a ward maid in the ward?—A. Always when we are having lunch the ward maid is about in the wards.

The Commissioner : The ward maid is about. (To the Matron) : Matron, Sister has correctly represented the custom of the hospital in that matter—that it is the general custom that all the nursing staff may be out of the ward for that quarter of an hour?

The Matron : Not invariably so in the medical wards. A nurse usually stays while the other ones are getting lunch.

Q. Usually in a medical ward there is at least one serious case?—A. And it is so in the surgical wards if there is anybody ill.

### GENERAL ABSENCE OF WARD ETIQUETTE.

The Commissioner : Yes, so she has explained. We go on to 14 now : that "there was a general lack of ward etiquette." Under the head of "ward etiquette" we have here :—

"The house surgeon and house physician report that when doing the regular morning round he has often walked through front and back wards without seeing any member of the nursing staff, and this has been especially noticeable in Ward 6. The H. S. says he has several times gone to the back and found no one there."

Of course, that represents very much what we have investigated already.

"Ward etiquette under Sister Hill.—Regret ward should have been found empty. Balconies a source of difficulty. Dr. Shepherd was asked to call if he did not see the nurse at once on entering the ward."

And Dr. Shepherd had patients apparently obliging enough to take that duty upon themselves and save trouble. Mr. Baker, would you ask for a definition of ward etiquette?

Mr. Baker (to Dr. Shepherd) : By this charge of ward etiquette do you refer to the general attitude of the nursing staff towards the residents?

Dr. Shepherd : Yes. It is a generalised charge, including the one which we have just gone through.

Q. Does it refer to the absence of courteous services?—A. Yes.

Q. Well, can you give instances of that? It is difficult for me to suggest them rather.—A. Yes; well, if you have been examining a case, or been tapping, auscultating, or anything of that sort, you naturally wish to wash your hands or something afterwards. Well, as frequently as otherwise you are allowed to pour out the water for yourself.

Q. Are there no other specific instances you want to suggest to the Commissioner? I will put it as evidence to Dr. Shepherd. We have already one instance in evidence—Sister Hutchins?—A. Yes, that is another point.

Q. What was it?

The Commissioner : Does this come under ward etiquette?

Dr. Shepherd : No; this comes under a rude remark in the ward.

Mr. Baker : I am suggesting a specific instance of lack of courtesy.

Mr. Procter : Shall we have Sister Hutchins here?

The Commissioner : We shall be able to judge when we hear the charge.

Mr. Baker : It was an admission which I obtained from Sister Hutchins yesterday that she had never quarrelled with Dr. Shepherd, but on one occasion she told him his behaviour was stupid.

The Commissioner : I don't think I should go into that.

Mr. Baker : I am not going into it any further, but I am suggesting it as an instance.

The Commissioner : Yes, we should have to have more of the conversation.

Mr. Baker : Well, whether it was justified or not, I think, is another matter to whether it was courteous or not.

The Commissioner : It is for you to say whether you will go into it, but I don't think myself I should pursue it.

Mr. Baker : I think I ought to ask Dr. Macqueen a general question whether he corroborates that. Dr. Macqueen, have you heard what Dr. Shepherd has said?

Dr. Macqueen : Yes.

Q. Do you corroborate it?—A. I corroborate all that he has said.

The Commissioner : Is it a rule of the hospital that they should pour out water for the residents?—I don't suggest it as a rule.

Q. Very much depends in these matters upon the maintenance of friendly relations as distinct from purely official relations?—A. I suppose so.

Q. I suppose so. It is the experience of all of us?—A. Yes.

The Commissioner : You don't want to carry that further, Mr. Baker?

Mr. Baker : Certainly not.

The Commissioner : Then the laying-out of bodies is the one we have dealt with, I presume?

Mr. Baker : Yes.

### EMPLOYMENT OF EXTERNAL NURSES.

The Commissioner : "Frequently external nurses had to be obtained, as the hospital staff was so inadequate." I suppose the emphasis there comes from the hospital staff being so inadequate. Of course, it is a merit to have got nurses anyhow if the staff is inadequate, but what evidence have you that the hospital staff is inadequate? Do you wish to give anything in support of that?

Mr. Baker called Dr. Shepherd.

Mr. Baker : Was it frequently the practice to have two or three extra nurses, or do you call them external nurses?—External nurses.

Q. External nurses in the hospital?—A. Yes, two or three.

Q. Three external nurses?—A. Two, I can confidently say.

Q. How often did that happen?—A. Oh, sometimes during my term one a week, sometimes not one for two or three weeks; and then sometimes two or three in.

Mr. Baker : I don't know whether this is challenged, but my instructions are that it can be proved from the Matron's books.

Mr. Procter : I don't dispute that we had external nurses. It is rather a merit than otherwise.

Mr. Watson : It may be so. That is not the question.

Mr. Baker : Dr. Macqueen, do you corroborate that?

Dr. Macqueen : Yes, and even with those external nurses——

The Commissioner: Yes, that is the point. Obviously it is a merit, as I have said, to call in external nurses if they are wanted, but what I want to hear is your evidence that the regular hospital staff is inadequate.

Dr. Macqueen: Well, even with those external nurses there was not a sufficiency at nights to run the theatre if there was an operation.

*Q.* Then it is the inadequacy of the theatre staff at night?—*A.* Well, there is no theatre staff at night.

*Q.* Have you any other parts of the hospital where you regard the staff as inadequate?—*A.* Well, of course, it came up over the case of the one special acting in two cases.

The Commissioner: Yes, which of course we have.

Mr. Baker: And in the case of the nurse with three or four months' experience in the Children's Ward.

The Commissioner: Yes, that was inadequacy in the sense of inefficiency.

Mr. Baker: No, insufficiency. You remember the Matron told us that she did not want to call up anybody in the night and disturb two or three wards.

The Commissioner: No, I understand that the charge was that a nurse of four months' training took charge of the ward. But if she was capable she was a nurse. You don't want more than one nurse.

Mr. Baker: No, but arising out of that question the question seemed to be raised of insufficiency, because she was called up specially, as I understood it, and if they had had plenty of nurses available she would not have been put there.

The Commissioner: I am afraid I did not take that point. (To Dr. Shepherd): Did you put that point forward?

Dr. Shepherd: No, not quite.

Mr. Baker: No, I put it forward now, arising out of the answers given by Dr. Shepherd and the Matron when dealing with this.

The Commissioner: Then what I have to consider now is the absence of a theatre staff at night, the question of want at times of "specials," that only one "special" was employed when two might have been usefully employed, and then I must ask Mr. Baker to formulate once more——

Mr. Baker: Certainly. As a specific instance, if you want them, of cases where there were insufficient nurses, I crave in aid the case of the inexperienced nurse who was in the Children's Ward—two nights, wasn't it?

The Commissioner: A night and a half.

Mr. Baker: A night and a half. Because there were no other nurses who could have been got to do that work without disinfecting the two who were in the isolation ward.

### THE STAFF: REPORT BY DR. MACDONALD AND MR. GAYNER.

Mr. Procter: On the question of the staff, I refer to a report, of which you have had a copy, by Dr. Macdonald and Mr. Gayner. Would you like me to read it?

The Commissioner: I would like Dr. Macdonald to be called. It is a report by Dr. Macdonald and Mr. Gayner on an inquiry held by them into the nursing and other matters in connection with the inner working of the York County Hospital. We want so much read as concerns the nursing staff. I will read this:—

"The specific charges against the nursing in the hospital have occupied little of our time, and, with small exceptions, have been found to be either entirely groundless, or trivial and petty.

"The case of the man who fractured his femur while in hospital appears to have been an entirely unavoidable accident. This man, who had been admitted for a traumatic synovitis of the knee, had been allowed up, and was to have been discharged from the hospital on the day of his accident.

"The question of the adequacy of the night staff has occupied a much larger portion of our time, and, indeed, the question is so large that we must consider it separately in relation (1) to the work of the wards; (2) the work of the theatre; and (3) the provision of 'specials.'

"(1) On the one hand, we have been told that the night staff is so short that it should be increased by the permanent provision of a night extra—*i.e.*, a nurse to be moved about the hospital wherever the work is heaviest. On the other hand, it has been clearly pointed out that if such a nurse is provided (*a*) each night nurse considers her own ward the most in need of help; (*b*) there is a tendency for those nurses who do not get this extra help to call for the assistance of the night sister at the least push. Nevertheless, we are inclined to think that the difficulty of providing extra help at night will be best met in the future, as often in the past, by the provision of a night extra. And in making this recommendation we do not suggest any alteration of the existing custom whereby (*a*) the Matron allots extra help to a heavy ward; (*b*) the night sister temporarily removes a nurse from a light ward, leaving the flat in charge of one nurse only.

"(2) The staffing of the theatre at night. Here, again, we have been met by the demand for increased help, and also with the objection that a member of the existing night staff has on occasion been employed in the administration of the anæsthetic, and thus diverted from her allotted duty.

"Also that operations have been wantonly deferred till the day staff had gone off duty.

"We are, however, inclined to think that the theatre night staff must be increased from its present number of two nurses by the addition of the theatre nurse, care being taken that the time so taken from the hours of sleep be made up to her later.

### PROVISION OF "SPECIALS"

"(3) Provision of 'specials.' In this regard some amazing evidence has been put before us of the hurtful and wasteful use which has been made of the hospital resources. Day and night 'specials' have been retained for a patient who was up and about the ward, and in another case day and night 'specials' were kept on for three weeks, yet the honorary in charge of the case has assured us that it would have been quite sufficient to have had 'specials' for forty-eight hours only. This is a very grave matter, and we would remind the members of this Board that by Minute of October 4, 1912, they themselves have ruled that if 'specials' be ordered for any case such order must be confirmed in writing by the honorary medical officer in charge on each occasion he visits the ward. Also we would hope that the ordering of 'specials' will only be done after the most careful consideration. It is obvious that provision of 'specials' is a matter of extreme difficulty to the Matron. She is allowed thirty nurses, and we have been told that on one occasion she had to provide as many as eight 'specials'—*i.e.*, nurses who may not leave the patient's bedside, but must employ other nurses to carry and fetch for them. We have been greatly impressed by the evidence put before us on this question. 'Specials,' we have been told, make some patients restless when they are there, and others restless when they are taken off. Also that the use of 'specials' makes the nursing in the ward generally much more difficult, as the ward nurses dislike having the care of their patients withheld from them during the most acute portion of the patient's illness. This feeling is, of course, accentuated when the 'special' has to be provided from outside the hospital. Except in the case of patients in isolation, delirious patients in a side ward, and some cases of tracheotomy, we feel that 'specials' are nearly always a disadvantage. It is better that the sister should apply to the Matron for extra help in the ward.

"The Eye Ward Staff.—The work in this ward has been enormously increased since Dr. Anderson's time; more and much larger operations are now regularly performed in the ward; the new theatre requires more work than the old, and the greater number of patients now having general anæsthetics all mean extra work. Further, there seems need in the theatre during operations for two nurses of experience.

"The eye ward, with its twelve or thirteen in-patients and its out-patients twice a week, scarcely seems big enough to demand the constant staff of one sister and two nurses; yet it is clear that an extra nurse is required from 1 o'clock onwards on operating days, and also on the nurses' half-day and on Friday mornings. Further, that there is need for two nurses with special experience during the actual operations on Mondays. This last is, of course, most difficult to provide in a hospital like ours, where the nurses come for training, and are necessarily constantly moved about the hospital. We suggest, however, that the senior casualty nurse be called on for this duty. Monday is, of course, the heaviest casualty day in the week, and there are X-ray patients to be seen in the afternoon. If this arrangement is made it is clear that more casualty duty will be thrown on the sister in Ward 1 in the future than in the past, and also that there will be need for an additional nurse on the staff.

"We have been told that our staff is already high in proportion to the number of patients in the hospital. But we would point out the great amount of work occasioned in this hospital by reason of the number of rooms into which each ward is divided. This is, of course, an immense advantage in treating patients, but it is heavy on the nursing staff. The eye ward, for instance, may have patients in four rooms, and in the other wards our valuable balconies have done much to spread the work out."

### INCREASES IN STAFF RECOMMENDED.

The Commissioner: And finally, I think, you recommend some increases of staff. Is that summarised anywhere?—Yes, on page 8 are the recommendations to the House Committee:—

"We would respectfully suggest the importance of the provision of a sick room for nurses, and we would ask that the question of the mode of warming the wards be made the subject of further experiment, the present grates being, we believe, in most cases costly, dirty, noisy, and inefficient.

"Our inquiry leads us to the conclusion that there is need for an increase in the nursing staff in the eye ward.

"That an extra nurse is needed in the operating theatre at night.

"That the nurses' half-day should begin at 2 p.m. instead of 3 p.m.

"That the side ward in 'Victoria' should for the present be ear-marked for a nurses' sick room, and that consequently the isolation block will be liable to be more readily called into use. To carry this out will entail some increase in the nursing staff, and we would respectfully ask that the numbers by which it should be increased should be considered in consultation with the Matron, and, if at all possible, supplied."

Mr. Baker: It seems to bear out entirely the charge of my clients.

The Commissioner: Probably you will not require to put any questions.

Mr. Baker: No, sir.

The Commissioner: I may congratulate you (Drs. Macdonald and Gayner) upon producing an extremely lucid report which seems to me to deal with the defects in the hospital administration in an admirable manner. Have you anything to add?

Dr. Macdonald: I do not think I have anything to add except this, that if we had not a Treasurer and had an unlimited amount of funds we might have asked for very much more.

The Commissioner: Briefly, you are astonished at your own moderation?

Dr. Macdonald: Yes, if you like, sir, but within the limits of what we can do I consider our hospital is fairly well staffed, and our number of nurses to patients is, I think, somewhere about the average as given in Sir Henry Burdett's annual.

The Commissioner: What does it work out at?—One nurse to 3.6 patients.

The Commissioner: Now we come to "Food and Accommodation Faulty."

Mr. Procter: I would refer you to a minute of the House Committee in which that report is dealt with.

The Commissioner (reading): "The report on the nursing question was considered, for which this special meeting had been called, and the recommendations to the House Committee, commencing on page 8, were taken *seriatim*. The provision of room for sick nurses and the question of isolation accommodation were referred to the Medical Board. *Re* heating arrangements; this matter was left with Messrs. Jalland and Bickel. The following suggestions were approved and referred to the Matron to carry out if circumstances permit: Nurse to be at call for night work in the operating theatre; nurses' half-day to commence at 2 p.m." That is all that deals with the nursing question.

Mr. Neden: I think that is all.

### FOOD AND ACCOMMODATION FOR RESIDENTS.

Mr. Procter: There are one or two general questions I want to put to Dr. Charles, as a former Resident. (To Dr. Charles): You were Resident here before Dr. Shepherd?—Yes.

*Q.* You know that in the columns of The Hospital some of the charges are with regard to the food and accommodation of the Residents?—*A.* Yes.

*Q.* What have you to say about the treatment that you received?—*A.* All I can say is this——

Mr. Watson: This was before these people came on the scene.

Mr. Procter: The treatment is the same.

Mr. Watson: I do not think that carries us any further. It was before these gentlemen came.

Mr. Procter: He was here with Dr. Macqueen for part of the time.

Mr. Watson: Oh, well, any time that Dr. Macqueen was here.

Mr. Procter: And I am prepared to say also that the treatment is the same now.

The Commissioner: Tell us as to the treatment while Dr. Macqueen was here.

Dr. Charles: During the time that I was with Dr. Macqueen——

*Q.* How many months was that?—*A.* I should say three or four. About four. I must say that the food, to my mind, was splendid. I have been four years a house surgeon in Hartlepool, King's Lynn, York County, and now Ipswich and East Suffolk Hospital, and it beats all of them.

Mr. Baker: Then as to the accommodation, have you anything to say?—Well, the accommodation, of course, you have seen. My room was a wee bit small.

*Q.* Have you any complaint of the laundry work?—*A.* The laundry certainly as regards collars could be improved, but that is the same in all hospitals.

Mr. Watson (to Mr. Baker): As counsel, do you really continue your suggestion as regards collars being properly got up?

Mr. Baker: No.

Mr. Watson: With regard to cracked plates, you are not going to introduce that?

Mr. Baker: No, unless it is pressed. The reason it has been alleged is as instancing the objections that were made as to the food. I do not call attention to the details at all.

Mr. Watson: It is really so trivial, and with regard to the plates, Mr. Macqueen quite readily withdrew these allegations.

Mr. Baker : It is only to emphasise that the complaints were made. I must ask one or two questions.

Mr. Watson : The other things you withdraw?

Mr. Baker : The details? Yes.

Mr. Watson : As to the collars. Is it worth your while to deal with these sort of things when there are other suggestions made?

Mr. Baker : I am perfectly willing to withdraw them, and do withdraw them.

Mr. Watson : Unreservedly?

Mr. Baker : Yes, as to the cracked plates and collars, but as to the food there are questions which I must ask. —(To Mr. Charles) : I must put this to you. Dr. Shepherd came in September, didn't he?

Dr. Charles : Yes, that is my recollection.

*Q.* On his arrival, or shortly after his arrival, did you have any conversation with him about the food? Do you recollect?—*A.* No, not to my knowledge. I may have said that the food was good. I told Dr. Macqueen the same.

*Q.* I put it to you that you said to Dr. Shepherd that the food was beastly?—*A.* No, Sir; not to my knowledge. That I never said of any hospital. I could not truthfully say it about the County Hospital, York.

### LETTER FROM DR. CHARLES.

*Q.* Did you write a letter addressed to both of them on January 20 of this year from 37 Belsize Park, Hampstead? Is that your writing (handing letter to witness)?—*A.* Yes, I think that is my letter.

*Q.* Well, have you any doubt about it?—*A.* Yes.

The Commissioner : You mean you have not any doubt about it?—Yes, I do not think so, Sir.

Mr. Baker : I need not read the first part. Towards the end you say, "I feel sure they will have some difficulty in filling the post." I do not know whether I ought to read the whole of this letter.

The Commissioner : What does it deal with?

Mr. Baker (handing letter to Commissioner) : It is the passage I referred to which I suggest to Dr. Charles refers to conditions of treatment of resident surgeons and physicians.

Dr. Charles : I think you are going into a personal matter.

Mr. Baker : Well, I am bound to put this.

Dr. Charles : It is outside this inquiry altogether. It is a personal matter concerning myself, and in which I will not allow my mind to be biassed against the institution. To my recollection, I think that letter was marked "Private" on the envelope.

Mr. Baker : Well, we have the envelope here. (Envelope handed to witness.)

The Commissioner : Most of this letter I don't think it would be proper to read, but there is this statement referring to the York County Hospital : "I feel sure they will have some difficulty in filling the post." This is written when he knows that Dr. Macqueen is going. "At least, that is my wish, and I am sure it is yours."

Dr. Charles : But that is a personal matter, which has nothing to do with this inquiry as regards the institution. I would not allow my mind to be biassed as regards giving information. It is a matter that really has nothing to do with the inquiry. It is a personal matter concerning my brother and myself.

The Commissioner : I have not read anything of a personal character.

Dr. Charles : I don't see anything in relation to food in that.

Mr. Baker : I agree with you that food is not mentioned.

Mr. Procter (after reading the letter) : Does this refer to something of an entirely private nature?

Dr. Charles : A personal matter, entirely.

Mr. Procter : A personal matter, clearly.

Dr. Charles : A matter not concerning myself at all. It is a personal matter, and should not be brought into this inquiry.

*Q.* It has nothing to do with food?—*A.* No, not concerning myself, but a near relation of mine.

The Commissioner : But still you are hoping that at the hospital they will find some difficulty in filling the post. Surely it is fair to suppose that your sentiments were not altogether friendly towards the hospital?—Yes, that may be true, but as regards giving a true version of what happened I would not allow anything to bias my mind.

Mr. Watson : It is obvious, as you wrote that letter with regard to a relation of yours, your feeling was not very friendly towards the hospital. How do you come to give evidence here to-day? It either strengthens your position or not. Were you willing to give evidence here? —Not as regards my previous experience here.

The Commissioner : I have nothing further about this food and accommodation question except what appears in the columns of The Hospital newspaper. The question of food is withdrawn, I understand?

The Legal Assessor : There is this paragraph in The Hospital : "Regarding our personal treatment in the said hospital; our living apartments were, in our opinion, in an extremely unsatisfactory condition," and it goes on to refer to a report by Sir Henry Burdett. Do your clients wish to speak on that, Mr. Baker?

Mr. Baker : Very shortly; I wish Dr. Shepherd to speak about the condition of his bed. You know, Dr. Shepherd, that the Commissioner has had an opportunity of seeing these rooms. Have you also?—Yes.

*Q.* Were they in the same condition as when you were here?—*A.* No.

The Commissioner : Have you seen them now?—Yes, one was done just before I went.

*Q.* What do you mean by "done"?—*A.* Re-painted and re-papered.

Mr. Baker : When?—Either just before or immediately after Christmas.

*Q.* What was the condition of which you complained before?—*A.* I think that the room was exceedingly damp, and that there seemed to be a considerable number of papers on the wall, different layers of paper never taken off.

Mr. Baker : A process very well known to decorators, I believe.

The Legal Assessor : Will you ask Dr. Shepherd if he has been to a public school and seen the dormitories?

Mr. Baker : Have you been to a public school?—No.

*Q.* What school were you at?

The Commissioner : Does that matter?

The Legal Assessor : No, no, I don't mean that. I only wondered if he had seen the ordinary dormitories of an average public school.

Mr. Baker : There was an occasion just before you made the complaint, when you found that three papers were coming off the wall, and flapping in the night, and you pulled them off, and after that incident you made a complaint?—Yes, I made a complaint before, but no notice was taken of it, and the paper annoyed me, so I pulled it off when it was flapping.

*Q.* In consequence of that your room was re-papered? —*A.* I put it in such a condition that it had to be done. (Laughter.)

### LIVING APARTMENTS UNSATISFACTORY.

The Commissioner : That is the basis of your statement that your living apartments were extremely unsatisfactory? —Yes, the other rooms were never very clean at any time.

Mr. Baker : Oh, the other rooms were never very clean; what rooms?—I refer particularly to the dining-room and the sitting-room.

Mr. Baker : What do you mean by not clean?—They were dirty. (Laughter.)

*Q.* You do not mean that the rooms were not dusted?— *A.* They were properly dusted as a rule.

*Q.* Do you mean that?—*A.* They were dusted all right generally, but at other times they were not. I refer to the condition of the wall paper. It seemed to be extremely old.

*Q.* Do you know if they were also re-papered?—*A.* I cannot say; they may have been cleaned down.

The Commissioner : Would Dr. Macqueen like to speak as to this?—Only to corroborate Dr. Shepherd.

Mr. Baker : Was it in your place that there was a flapping wall-paper?—No, it was not.

The Commissioner : Did the dampness in the wall disappear when the new paper was put on?—I have not had an opportunity of seeing the rooms since.

*Q.* Would you mind having a look now?—*A.* Certainly not.

Mr. Procter: I thought you had unreservedly withdrawn all these.

Mr. Baker: We have not withdrawn any of these.

The Commissioner: I should be sorry for anything to be withdrawn on a technicality, as I never did myself understand that the question of accommodation was withdrawn.

Mr. Baker: Nor is it true in regard to the living apartments; I said we would not carry it further, but I do not think it is right to say these charges are withdrawn. It is not fair either to you or my clients.

The Legal Assessor: The question of accommodation was never mentioned.

Dr. Macqueen returned, and was asked by the Commissioner: Is there any sign of damp where the old damp was?—No, there does not seem to be any sign of damp just now.

The Commissioner: You are smiling; I am only admiring your caution. Of course, the important question was whether there was any permanent damp or whether it was some temporary cause which has now been remedied, and that appears to be so according to your remark?—Yes.

Mr. Baker: Have you examined the bedrooms?—Yes.

*Q.* Is it entirely different to what it was in your time?—*A.* Yes, it has been remedied.

The Commissioner: Which room do you consider to be in the worst condition?—It is a difficult matter to say.

The Commissioner: I have looked at the rooms and I have not the least doubt?—The dining-room.

The Commissioner: Yes, certainly. That room is, I believe, used not only by the residents but by the visiting staff?—Yes.

Mr. Procter: Mr. Jalland wonders whether you would like to ask him any question about the paper?

The Commissioner: I have seen the paper, and I do not think I need ask him any questions about it; I have formed my own opinion from seeing it, thank you. (To Mr. Baker): Now, I don't want to take advantage of anything you have, rightly or wrongly, withdrawn. Is there any more call for observation on food, etc.?

Mr. Baker: No.

### THE RESIGNATIONS OF DR. MACQUEEN AND DR. SHEPHERD.

The Commissioner: Then we come to the resignations.

Mr. Baker: You can put it on record by both standing up and having the letters read.

The Commissioner: You would like me to read first their letter of resignation?—That is how it comes in order of date. Perhaps if I ask Dr. Shepherd if he wrote the letter.

The Commissioner: I have a letter which is undated, but which I take it is the letter referred to.

Mr. Baker (to Dr. Macqueen): Is this letter [produced] in your writing?—It is signed by myself and Dr. Shepherd.

*Q.* It is undated?—*A.* It was written on December 27.

The Commissioner: This is a letter of which the date is accepted as December 27, 1912, addressed to the Chairman of the House Committee, York County Hospital: "Sir,—We the undersigned, being the resident medical officers of the above institution, wish to make a report on the condition of affairs existent in this hospital. As was pointed out at the last meeting of your Committee, if we wished to complain we should do so formally in writing to the House Committee. We do now, in pursuance thereof, make formal report and complaint. Ever since the time——"

Mr. Baker: So far as I am concerned, the only thing we require is that the resignation was sent in on that date.

The Commissioner: Every other part of this letter except this end piece has been considered, but, leaving those out, I will come to the relevant paragraph as to the resignation: "Unless radical changes along these suggested lines are promptly made, we, the undersigned, cannot do otherwise than to tender you our resignations separately and conjointly. We have struggled hard to raise the efficiency and good name of the hospital during our short tenure of office, but without aid such things are impossible. Any promise of this ancient institution's future usefulness can never be fulfilled under such conditions as exist at present." Then there was a special meeting of the House Committee on December 31, the minutes of which state that a letter sent by Drs. Macqueen and Shepherd was read, in which they complained generally of the internal administration of the hospital, and principally with regard to the nursing. They also refer to the condition of their living rooms, which they consider were not as comfortable as they should be, and quoted from Sir Henry Burdett's report of January 27, 1912. They concluded by saying that if changes were not promptly effected they would tender their resignations. Mr. Jalland reported that as advertisements could not have been got in for insertion in the medical papers of December 28, they had been held over to give the officers an opportunity of apologising.

The Commissioner: The date of that is December 31. But before that comes an extract of the House Committee's minutes of December 24. The Committee were informed that on Sunday, the 15th, the resident officers had broken their tea-service, as they contended it was unfit for use owing to its cracked condition. The broken pieces were examined, and from what could be seen the Committee considered that the action was not justified. They were interviewed separately, and ultimately a resolution was adopted that the two resident officers be given one month's notice, and leave of absence previously granted be withdrawn.

### THE MEETING OF THE HOUSE COMMITTEE.

The Commissioner: The first question that arises is, What happened at the meeting of the House Committee? These gentlemen attended and a certain resolution was passed. Were these gentlemen present when the Committee came to this conclusion to terminate their engagements? I presume they had left the room?

Mr. Neden: Yes, sir.

*Q.* Did you write them an official letter at once saying that their engagements were terminated, or was the official letter held over in view of the explanation which I have read?—*A.* Yes, the letter was held over until they were given an opportunity of apologising. I communicated this decision to them verbally in my office. They were both present, and I believe the words used were something to this effect: that there was no need to decide at once, that they would have a day or two to think it over, but they might let me know within a day or two. I asked them on the Saturday following, the 28th, whether they had come to any decision on the matter, and they said they had written on the whole subject to the Dean, who is the Chairman of the Committee.

The Commissioner: I think the chronology is quite plain. On the 24th the Committee decided to give a month's notice, but for several reasons there was no official letter sent to that effect, and on the 27th they wrote the letter to you which I have already read.

Mr. Baker: I think it is denied that this verbal communication was made.

The Commissioner: One wants first to get a clear view of what happened. Then on the 27th they sent in this letter, which I shall have to ask whether it is a resignation or a conditional resignation?

The Legal Assessor: I think it is a conditional resignation.

### FURTHER LETTER FROM THE RESIDENTS.

The Commissioner: Then they get a communication from the Committee on the 31st, and then they write a further letter on January 7, which I am prepared to read, if you like, now, if they will admit that it is their letter. It is dated January 7, 1913. "To the Chairman of the House Committee, York County Hospital.. Sir,—We are in receipt of your communication of December 31, 1912, from the House Committee, and beg to state that we cannot accept the resolution therein stated as passed by them. Our refusal is for two reasons, viz.,

"1. That on December 27, 1912, we wrote you a letter in which we resigned our posts as resident officers of this institution, our resignations therefore dating from that date.

"2. That even though our resignations had not been handed in, such dismissal by the House Committee is not in accordance with the rules which were in force at the time of our appointment. The rule to which we refer is this—viz., 'The house surgeon and the house physician shall be appointed and be removable by the House Committee and members of the honorary medical and surgical staffs.' As we were appointed by the combined board, our dismissal must also come from the combined board.

"We cannot restrain ourselves from expressing the opinion that in cancelling our holidays you adopted a very 'dog-in-the-manger' attitude. The house physician was in ill-health during the week prior to Christmas, and was advised by one of the honorary staff to go away for a holiday after the illness; but as the work of Christmas time was so heavy, he decided to stay on and 'see it through' in the knowledge that he would be having a holiday so soon after, but this is how he is treated. The house surgeon did double duty whilst his colleague was in ill-health, working practically night and day, and this is his reward.

"If we took premature action in breaking the cracked dishes before acquainting the House Committee we regret that action, but we maintain that they should not have been put before us, and we do not retract one iota from the statements made in our letters concerning the hospital and its internal affairs. If you, as a house committee, wish to see your hospital prosper, it is time you gave those internal affairs your most serious consideration."

The Commissioner: Now, I think, Mr. Baker, we have all the facts.

Mr. Baker: First of all, I want to ask Mr. Neden about this Saturday when you say you made the verbal communication to both Residents.

The Commissioner: Following the meeting of the House Committee on the 24th at which it was decided to give them notice.

Mr. Baker: What date was that?

Mr. Neden: The first communication I made to the Residents was on December 24, immediately after the meeting.

*Q.* I want you to particularise; how was it made?—*A.* By word of mouth.

*Q.* First of all, when did the Board sit?—*A.* At 11.30.

*Q.* How long for?—*A.* Approximately half an hour or three-quarters.

*Q.* And when do you say you spoke to the Residents?—*A.* They came into my office, I believe, before I went to lunch, probably about one o'clock.

*Q.* What happened?—*A.* They came and asked me the result of the meeting, and I said—"Well, of course, they have gone into it and do not agree with your action, and they have decided to give you a chance to apologise." They used words saying they did not intend to apologise; they made it very plain that they did not intend to apologise and asked what would be the result, and I said, of course if you don't apologise it would take effect and they would have to go.

*Q.* And then no definite communication was made to them that there had been a resolution passed that they were dismissed until December 31?—*A.* I considered that would be a definite communication then.

*Q.* I don't want to know what you consider, but I ask if there was no definite written communication before December 31?—*A.* No communication was made in writing.

*Q.* There is no doubt, is there, that the resolution was the decision of the House Committee alone?—*A.* At which were present representatives of the Medical Board.

*Q.* But was it a meeting of the Medical Board——?

The Commissioner: It is of no use wasting time. This was a decision of the House Committee, and technically it was not a decision of the House Committee sitting as a combined Board with the Medical Board?

Mr. Neden: No.

Mr. Baker: That is all I want to ask you.

Mr. Neden: "On the 16th January"—I am reading now from the minutes of the Medical Board—"it was unanimously agreed to approve the action of the House Committee with regard to the dismissal of the Resident Officers."

### MR. JALLAND'S STATEMENT.

Mr. Jalland: May I just mention a point on this? I think they scarcely quite know the position. On our House Committee there are two representatives of the Honorary Medical and Surgical staff to represent the Medical staff in all questions that come before the House Committee, and at this meeting we had the two representatives; they were there confirming the dismissal of these two Residents. A special meeting of the Medical staff was called soon afterwards.

Mr. Baker: This is, of course, a purely legal question which is being raised; I don't know whether you want to go into it.

The Commissioner: I don't like to interrupt any remarks Mr. Jalland would like to make, but I think I am thoroughly in possession of your views and only wish to hear what Mr. Jalland wishes to say.

Mr. Jalland: This was passed at a meeting of the House Committee with representatives of the Medical Board, and at a meeting of the Medical Board called soon after they unanimously confirmed the action then taken. That is all I wish to put before you, Sir.

The Commissioner: I thoroughly understand that.

Mr. Baker: I would just ask the doctors quite shortly: Dr. Macqueen, did you have any communication from the Secretary on December 31 that there had been a resolution passed purporting to dismiss you?—I did.

*Q.* Was that the first communication you received?—*A.* It was the first communication I received.

*Q.* Was that communication that that resolution was of the House Committee?—*A.* Yes.

*Q.* Have you got it?

The Legal Assessor: If this is going to be argued, I think you will agree with me that that letter is a qualified resignation?

Mr. Baker: Certainly; I don't know what point is being made here.

The Legal Assessor: I don't think any point is being made. It seems to me that that letter that you sent was a qualified resignation, and then they were asked to resign, and it is pretty obvious. It seems to me quite immaterial. The Hospital behaved as well as they possibly could have done, and gave them an opportunity to apologise. There is no reflection on your clients; they behaved as well as they could. There is no point in it at all.

The Commissioner: Well, now, we come to the last page:—

### THE DAY BEFORE DEPARTURE: TESTIMONIALS, THE HONORARIES AND THE RESIDENTS.

We terminated our engagements at York County Hospital on January 23, 1913.

On the day prior to the appointment of new Residents one of us asked a member of the honorary staff for a testimonial. He said that he would consider it. On the same evening a second member was also asked for a testimonial. He said that it had been suggested that we should get testimonials if we would promise not to say anything to the candidates who were coming the following day regarding the condition of affairs existent in the place. On the following morning the member of the honorary staff first referred to came to hospital and informed one of us that he had considered the matter and that he would grant a very good testimonial if an undertaking was given him that we should say nothing about the place to the candidates who were coming that day. This appeared to us to show that these testimonials were to be granted in the light of "hush-money," and they were refused. Both men admitted that our work both medically and surgically had been extremely satisfactory.

Mr. Baker: I will call upon the doctors to say who they were.

The Commissioner: Yes, I think that is a very important question.

Mr. Baker: Dr. Macqueen, on the day referred to who was the member of the honorary staff you asked for a testimonial?—Dr. Macdonald.

*Q.* You asked him for a testimonial?—*A.* Yes.

*Q.* What did he say?—*A.* He said he would consider the matter.

*Q.* The same evening did you ask anybody else?—*A.* Yes, I asked Mr. Gostling.

*Q.* Was anybody else present?—*A.* Mr. Shepherd.

*Q.* What did Mr. Gostling say?—*A.* Mr. Gostling said it had been suggested by Mr. Jalland that we should get testimonials provided we said nothing to the men who were following us about what the hospital was like.

*Q.* Then on the following morning did Mr. Macdonald speak to you?—*A.* Mr. Macdonald was at the hospital and spoke to me.

*Q.* Were you alone?—*A.* I was alone.

*Q.* What did he say?—*A.* He said he had decided the matter, and that he would give a very good testimonial if I would give him an undertaking that I would say nothing to the candidates who were coming that day concerning the hospital.

*Q.* Did you accept his conditions?—*A.* I did not.

*Q.* Did you accept Mr. Gostling's?—*A.* No.

*Q.* Was any question raised between you as to whether your work had been satisfactory?—*A.* No; both said at the time, of their own accord, that our work had been extremely satisfactory.

Mr. Procter: I don't wish to cross-examine. I will call Mr. Macdonald if you wish.

The Commissioner: It seems to me if I were in Mr. Macdonald's position I should consider it highly desirable. It is an extremely unpleasant charge against a professional man, or against any man.

### DR. MACDONALD'S STATEMENT.

Dr. Macdonald: It is, and I would like to say first of all that if I were doing the thing to-day, my action to-day would be a little different; but what was in my mind was this, and the reason for what I did was this: that when I give or receive a testimonial I always consider that it implies an assertion as to the loyalty of the person who receives it from me, to the extent which he has served, and also that he is a colleague with whom it is pleasant to deal. In fact, I do not care to give a testimonial unless I can say something to that effect and always feel that it is implied. Dr. Macqueen had committed one action, which I am willing to tell and amplify if you wish, which I considered was extremely disloyal to the institution; but, all the same, Dr. Macqueen had done good work for me, his relations with me had been pleasant, and I did not want not to give him a testimonial if I could. My final test in my own mind as to his loyalty to the institution was whether he would agree not to say to the candidates what he had been saying about the institution, whether he would agree not to prejudice them against the place. Because I think you will know if a young man comes into an institution and has a prejudice suggested to him he does not settle anywhere, and I well remember that that happened to myself on one occasion, when I was a new house surgeon in another place, where I was prejudiced against the post by a predecessor, and I did not settle there well at all, and I afterwards found that it was suggested to me quite unreasonably. That is, so far, my explanation and the reason I did this. But I did not say anything of this sort. I did not ask them to promise not to say anything to the candidates who were coming the following day regarding the conditions of affairs in the place. That seems as if there was something disgraceful which I wished to conceal. That was not the case at all. What I asked them not to do, and I don't think it was unreasonable, was not to prejudice the new man against the place. Now, Dr. Macqueen said "No," he would not give that undertaking; he used the word coercion. I think I said "No," it was no such question at all; the question was simply whether he would not prejudice the men, and if there was anything unsatisfactory in their relations with the Matron or in regard to food, etc., they would find it out for themselves. I knew that there was really nothing in it at all. I gave a testimonial to Dr. Shepherd because I understood in the first place that Dr. Shepherd was not primarily responsible for the trouble and was being carried away by his senior, Dr. Macqueen; and in the second place, Dr. Shepherd led me to understand that he would not do anything to prejudice the new men, which I think was very far from the action which he took when the new men were here; but I want emphatically to present it to you that that was not in any way in the nature of hush-money. It is an imputation I strongly resent; and I also strongly resent putting it in this way, that he promised not to say anything regarding the condition of affairs in the place, because that implies that there is something I wished to conceal. There is nothing I wish to conceal. I am proud of the institution, and think it does very good work, and have nothing to conceal, and am quite willing to stand by that. But again I would say, if asked to do it to-day I might take a different action.

Mr. Baker: With regard to Dr. Shepherd, you did give him a testimonial, didn't you?—Yes, I did.

*Q.* I rather gathered from your suggestion just now that you took an undertaking from him. Is that right?—*A.* No; he led me to understand it was something in this way——

*Q.* Do I understand that you took an undertaking from him before giving him the testimonial?—*A.* No; I said to him, as I handed it to him, "I understand, Shepherd, that you will do nothing to prejudice the new man against the place."

*Q.* Were these your exact words?—*A.* As far as I can recollect.

*Q.* What did he say?—*A.* As far as I can recollect, he said, "Oh, no, I have no wish to prejudice the men against the place"; I am quite clear about the meaning of the words.

### DR. SHEPHERD CALLED.

Dr. Shepherd was called and examined by Mr. Baker.

*Q.* You received a testimonial from Dr. Macdonald dated January 23, when he handed this to you?—*A.* It came by post.

*Q.* It came by post?—*A.* Yes.

Dr. Macdonald: It was during the morning, in close relation to it.

Mr. Baker: Did you see Dr. Macdonald that day?—I had not seen him for——

*Q.* When had you seen him before?—*A.* Two days before.

*Q.* Did you give him any undertaking such as he has suggested?—I gave him no undertaking whatever; I would take no testimonial on any undertaking.

**DR. MACDONALD.**

Mr. Baker: You gave that testimonial, and I think you wrote a letter to the *Yorkshire Evening Press* on March 15, in which you make use of the words "As to the doctors who have resigned, Dr. Macdonald said they had an exaggerated idea of their own importance." And after referring to their deficiency in tact and experience, and to their complaints as to the doctors' quarters, it goes on, I think——

Dr. Macdonald: That is not a letter; it is an interview with a member of the press.

*Q.* You did make that charge that they were deficient in tact?—*A.* Yes.

*Q.* Although you had given this testimonial: "Mr. W. M. Shepherd, M.B., Ch.B., has been House Physician to the York County Hospital for the last five months. Part of his duty was to act as House Surgeon in the Eye, Ear, Nose, and Throat Department. He had there to assist me with my out-patients, and had operations and had charge of the eye and ear wards. He was a valuable assistant, has a good knowledge of ophthalmoscopy, and could do refractions efficiently; and while he was a very competent assistant at operations and did dressings and the after-treatment of my operation cases, including several complicated mastoid operations, thoroughly, carefully, and well, I placed considerable confidence in his judgment, and felt quite comfortable in leaving my cases, including some which I left him to operate on, in his charge. His relations to me personally were always most pleasant, and I found him an agreeable colleague." Then follows your signature.

Mr. Procter (to Dr. Shepherd): You have told us you gave no undertaking to Dr. Macdonald?—Yes.

*Q.* Did you say anything that would lead him to suppose that you would not prejudice your successors?—*A.* That matter was going to be discussed, and I said, under those conditions just leave it alone.

*Q.* Is that all that was said?—*A.* Yes.

*Q.* Can you account for the other impression Dr. Macdonald has formed?—*A.* I cannot.

The Commissioner: Mr. Gostling is not in attendance, but I cannot believe that he would not desire to be when such a serious allegation is made.

Dr. Macdonald: He has a serious case on.

The Commissioner: We can hear Mr. Jalland. The point is this, that the evidence of Dr. Macqueen is that he was informed by Mr. Gostling that you (Mr. Jalland) had suggested that the question of whether he should have a testimonial or not depended rather upon keeping quiet about what had happened in the hospital?

Mr. Jalland: Yes, that had reference to their complaints about their food and things of that kind, which other residents absolutely denied. There was not a trace of it, and it was more the comfort of the institution and their living and feeding that I had in mind.

**MR. GOSTLING.**

The Commissioner: Was it your suggestion that the testimonial should depend upon their keeping quiet about any matter whatever?—Not in regard to any matters particularly. I said they should not try and upset the candidates which came.

*Q.* And unless they would undertake not to upset the candidates they ought not to have a testimonial?—*A.* Well, if I told them that——

Mr. Baker: But it is suggested that you made the suggestion to Mr. Gostling?—I very likely did.

The Commissioner: Then about this question of the testimonials. You have read these allegations, Mr. Gostling. We have had Dr. Macdonald, who has told us his position, and Mr. Jalland, who says it is possible that some observations attributed to him were made by him as to giving testimonials dependent upon some understanding with the Residents that they should not prejudice the place with their successors. What has been urged in regard to yourself is that you made the giving of a testimonial conditional upon some such undertaking?—Yes.

*Q.* Will you tell us what line you did take?—*A.* The line I took was that I would give Dr. Macqueen a testimonial if he would not, as I said, "crab" the place to the new men who were coming. It was just after the trouble with the Committee, chiefly about food and their accommodation. I had been a house-surgeon myself here years ago for two or three years, and I had been very comfortable, and I did not want to see new men come with a prejudice that the food and accommodation were bad. It would start them off in a bad way. That was the only reason why I made that conditional promise

*Q.* And, as a matter of fact, did you give either of them a testimonial?—*A.* I promised Dr. Macqueen one, but unfortunately I forgot to carry out that promise. I meant to give him one.

### THE TREATMENT OF THE NURSING STAFF.

The Commissioner: We will now take "The Treatment of the Nursing Staff." The charge in regard to this is as follows:—

"Regarding the treatment of the nursing staff, there seemed to be a general hesitancy on the part of the nurses themselves to report if they were ill. From an onlooker's standpoint, the only reason which we could assign for such extraordinary conduct was the ordeal (to them) which they had to undergo when they reported themselves.

"When they did report themselves there was in several cases a failure . . . to report immediately to the medical staff, and the sick nurses were often left lying in the nurses' home without a fire in their bedrooms—even in December and January. Illnesses too frequently were made light of by the Matron and sisters, and the nurses seemed to object to being placed in certain wards on account of the treatment meted out to them by sisters."

Mr. Baker: That is a general allegation followed by specific instances.

The Commissioner: We have no particulars; we cannot fill up the blanks.

Mr. Baker: No.

The Commissioner: Then we will go on to the specific instances. We will take first the case of the diphtheria, "where the patient had been in the nurses' home for several days prior to being seen by the Resident, or antitoxin administered."

Mr. Baker (to Dr. Shepherd): Was this case yours?—Yes.

*Q.* Was it handed over to you by Dr. Evelyn?—*A.* Yes, it was.

The Commissioner: It was Nurse Davis?—Yes.

Mr. Baker: And she was handed over to you as a patient by Dr. Evelyn?—Yes.

*Q.* Can you identify the date?—*A.* I cannot say the date.

The Commissioner: There is an extract from the House Committee's minutes of October 22, 1912: "The Matron also reported that Nurse Davis, who was stated at the last meeting to be suffering from sore throat (that, I take it, to be fourteen days before), had developed diphtheria, but was going on satisfactorily."

Mr. Baker: There is only one question I need ask you on that. Before she was sent up to you by Dr. Evelyn, had she, in your opinion, had diphtheria for several days?—She may have had; she must have had diphtheria before I saw her.

The Commissioner: Had Dr. Evelyn been attending to her long?—I don't know.

Mr. Baker: Had antitoxin been administered when the patient was handed over to your care?—Not to my knowledge.

*Q.* Do you know whether she had been in the nurses' home for several days?—*A.* She was in when I saw her.

*Q.* Do you know how long she had been there?—*A.* I cannot say.

The Commissioner: I have not the date when you saw her in the nurses' home? Neither have I got it. The date will appear on her chart.

Mr. Procter: The nursing staff are not attended by the Residents.

### THE CASE OF NURSE DAVIS.

The Commissioner: I thought about getting that through the Matron. (To the Matron): This is the case of Nurse Davis. You reported to the House Committee on the 22nd that Nurse Davis, who was stated to be suffering from a sore throat a fortnight before (so that would be October 8), you told the committee she had sore throat, and on the 22nd she had developed diphtheria?—I don't remember the dates.

*Q.* How many days had she been in the home before you called in Dr. Evelyn to see her?—*A.* Dr. Evelyn would see her the same morning she went to bed.

*Q.* Can you tell us what day she went to bed?—Wednesday evening, October 2.

*Q.* Dr. Evelyn would see her the same evening.—*A.* Yes.

*Q.* What were your general arrangements for the nursing staff?—*A.* We have a physician and surgeon for the nursing staff.

*Q.* What arrangements have you for looking after nurses when sick in the home?—*A.* The Assistant Matron and myself in the day-time and the night sister at night.

*Q.* Who, of course, has the whole hospital also to look over?—*A.* That is why the arrangements in the home are more difficult in the night-time than the days, because I feel anxious about the night sister being away for long, and the home is some distance away.

*Q.* But, at any rate, this nurse when taken ill was seen the next morning by Dr. Evelyn.—*A.* Yes.

Mr. Procter: In the case of nurses who require medical treatment, they are put in a separate ward?—In a special ward if it is not occupied; if not, they are put in the general ward with a screen round.

*Q.* In a special ward they are getting accommodation for which private patients are charged three guineas a week?—*A.* Yes.

*Q.* And they get equal treatment?—*A.* Yes.

Dr. Evelyn called and examined by the Commissioner:

*Q.* Did you, as a fact, see her on October 3?—*A.* Yes.

*Q.* Now we start fair?—*A.* I saw her on October 3, and saw that she had a suspicious throat, and I did what I always do in these cases. On coming away from the case I asked the house physician to kindly take a swab and give a dose of antitoxin.

*Q.* Did you give orders for the antitoxin before you had the result of the swab?—*A.* Yes, that is always my practice.

*Q.* How many units did you give her?—*A.* 4,000 units, and that is charted here. (Chart produced.)

The Commissioner: That disposes of the statement that it was several days before she was seen by the Resident or antitoxin administered. She went sick on the 2nd and had antitoxin on the 3rd.

Mr. Baker: Was October 2nd the first day you saw her?—I saw her on the 3rd.

The Commissioner: Then here is the letter from the nurse: "My Dear Miss Tute,—I must apologise for not answering your letter by return. I was away visiting and did not return home until too late for the post. Your statement was quite correct. I was reported to you on Tuesday, October 1, or on Wednesday, the 2nd, and was having antitoxin the same evening. I have no complaints to make against yourself or the sisters. I was treated with the greatest kindness all through, and was very sorry indeed when I read of the charges brought against the hospital."

The Commissioner: The next case is that of the "night nurse who was subject to heart attacks, and who was found on duty at night cyanosed and gasping for breath, in no fit condition to be even sitting up; and although ordered immediately off duty, had to remain two hours longer. There is a further reference to that in which Dr. Shepherd reported finding the nurse "deeply cyanosed and apparently suffering from cardiac failure," and asked that she be removed at once, as the heart condition demanded it.

The nurse was called and examined by the Commissioner: I want to hear about this attack you had at night. You were, it seems, found cyanosed and gasping for breath one night, and not in a condition to sit up. What have you to tell us about that attack?—I don't know. I just had a pain in my side. I don't know that I was cyanosed.

*Q.* How long had you been in the hospital?—*A.* I had been two years the previous April; it was then January.

*Q.* So you were in your third year?—*A.* Yes.

*Q.* How much sick time had you had?—*A.* Just a day or two.

*Q.* You had not been warded for any attack?—*A.* No.

*Q.* Have you been on duty since continuously?—*A.* I have not had any sick duty at all.

*Q.* Dr. Shepherd saw you and thought you should immediately go off duty? Did you hear him give any instructions to the night sister about it?—*A.* None whatever.

*Q.* Could he have given instructions without your hearing? Where were you?—*A.* I was in the ward.

*Q.* Were you present at the interview between Dr. Shepherd and the night sister?—*A.* Yes; I was giving my report to Dr. Shepherd, along with the night sister.

*Q.* You were going round with them when the doctor noticed your condition and thought you ought to go off duty?—*A.* Yes.

*Q.* And you did not hear him say anything about your going off?—*A.* No.

*Q.* Did you, as a fact, go off duty that night?—*A.* Yes, I went off about 2 A.M., as arranged.

Dr. Shepherd: I spoke to the sister as I was leaving the ward.

The Commissioner: So that the nurse might not hear?—Yes, she might not.

Mr. Baker: Have you had heart trouble in your time?

The Commissioner: I did not put that, because you are going to have an opportunity of hearing Dr. Evelyn. I put to her questions as to how much she had been off duty in the hospital, and she has told us that she has never been warded, and only off duty a few days. As to the condition of her heart, Dr. Evelyn can tell us.

Mr. Baker: My question was more directed to the time before she came here. (To witness): Had you trouble before you came here?—None that I know of.

### THE MEDICAL EXAMINATION OF NURSES.

The Commissioner: Matron, have you any arrangement for the medical examination of nurses before they are taken on the staff here?—Yes; they supply a medical certificate before they come, and when they have worked three months they are examined by Dr. Evelyn.

The Commissioner: That is what I expected to hear, and therefore I was waiting for Dr. Evelyn. Now, Dr. Evelyn, I have just heard that you examine these nurses after they have been a certain number of months in the ward. Is any record kept of your examination?—Nothing, other than that they are passed or refused.

*Q.* Is that kept in your register, Matron?—*A.* Yes.

*Q.* You have a note, but nothing more?—*A.* No.

*Q.* Have you the register of their doctor's certificate of previous good health, Matron?—*A.* Yes.

*Q.* Can you find this one?—*A.* Yes.

*Q.* Now, then, Dr. Evelyn?—*A.* I saw that nurse at Miss Tute's suggestion the day after she had had this cyanosed attack in the ward. I was not then given to understand that she had had this severe attack, and I somewhat superficially examined her and told the Matron to keep her in bed, and I ordered what I considered was necessary for her, and said I would see her again the next day. When I came into the hospital the next day the Matron said, "I wish you would see nurse, please; there is some question of her heart being bad." I examined her critically, and, asking nurse if she experienced any disability at all, she replied, "No, I do not; I feel a little bit out of breath." I said, "Do you think you can do some work?" and she replied, "Yes," and with that I said to the Matron, "I think nurse could get up and could come to duty as soon as you like." She went on duty, and has been on duty ever since.

The Commissioner: You say you examined her critically?—I found nothing wrong with her, no disability of any kind; in fact, I was given to understand that the patient was so ill in Victoria Ward that you could hear her heart if you stood by her, and that made me all the more anxious to examine her critically.

Mr. Baker : You say you did examine her critically and could find no cardiac trouble at all?—No.

*Q.* None whatever?—*A.* No.

The Commissioner : It is not suggested that you, Dr. Shepherd, examined her heart?—No.

*Q.* It was not such a murmur that you could hear across the room?—*A.* No, but she appeared extremely weak.

*Q.* You are not responsible for the statement that such a murmur could be heard without a stethoscope?—*A.* No.

Mr. Baker : Do you know, Dr. Evelyn, if she went off on holiday?—I do not know.

The Commissioner (reading report produced by Matron) : The medical certificate when she came for training seems to have been entirely satisfactory, her heart and other organs being described as "good."

Mr. Baker (to Dr. Evelyn) : Have you since that occasion ever warned her to go off duty?—I do not remember to have seen her since.

*Q.* Have you ever given explicit instructions that she should go off?—*A.* No.

*Q.* Prior to that occasion did you?—*A.* I don't know.

The Commissioner : I see that she entered on April 18, 1910, and she was passed by Dr. Evelyn on 22/8/1910, so that there were some months between the time of her beginning to work and the examination.

Mr. Baker : I will just ask Dr. Macqueen. Do you corroborate Dr. Shepherd ; were you in the ward at the time when Dr. Shepherd has told us that he saw Nurse Firth cyanosed and in a gasping condition?—I was not in the ward at the time, but I saw her shortly afterwards.

The Commissioner : And what condition did you find her in?—Cyanosed and gasping for breath.

### THE CASES OF SOME SICK NURSES.

The Commissioner : Then I will pass on to the case in which it is alleged that "a case of sinus suppuration and threatened frontal trouble was left in her bedroom in the month of December without a fire, and seemingly, no thought of it, until ordered."

Mr. Baker : Who was this nurse?

Dr. Shepherd : Nurse Bartholomew.

The Commissioner : Is she available?

The Matron : She is away for her holiday.

Mr. Baker : Do you know what treatment she had been having?

Dr. Shepherd : Treatment to sweat her. She did not improve, and her headaches got more severe, and Dr. Macdonald asked me to see that she was put to bed.

The Commissioner : Where?—In the Nurses' Home.

*Q.* And you saw her in the Nurses' Home?—*A.* Yes.

*Q.* The same day?—*A.* Yes.

Mr. Baker : How long after she was first put there did you see her again?—Either the next day or the day following that.

*Q.* Was there a fire in the room?—*A.* No.

Dr. Macdonald : This is a case of quite trifling importance, and to say that there was anything to take exception to in the treatment is absurd. I asked for a fire because I wanted a profuse sweating.

The Commissioner : That was the second day?—Yes.

Mr. Baker : In the month of December?—Yes.

The Matron : As a general case nurses don't have fires in their bedrooms the first day.

The Commissioner : This nurse did not appear to be physically ill?—No.

*Q.* Is it your recollection that by Dr. Macdonald's orders a fire was lighted the next day?—*A.* Yes, when it is necessary the nurses always have a fire, but generally when they are ill they do not require one in their room, because they are warded.

The Commissioner : We get to No. 4. "A case of severe abdominal colic first felt by the nurse on Sunday. She reported herself to the Sister on Monday : no notice was taken. She reported herself again, to the Matron, on Wednesday, and was not seen by a doctor until Thursday. At table this nurse had been noticed to have taken little or no food, and had asked one evening to be excused from attending the meal." Then we have a further report : "Nurse Scott, complaining of abdominal pain and attributing same to a natural cause, asked for castor oil and this was given her. Nurse Scott's half-day on Tuesday and although weather was very cold, she went out to friends to tea. Sister believes she vomited on Tuesday night." Do you wish to amplify that?

Dr. Macqueen : No, I do not wish to amplify that.

Mr. Baker : She reported herself to Sister Hannah?

The Legal Assessor : Yes, that is Sister Hannah's report.

The Commissioner : We must ask the Matron upon this report. Is it a fact that she reported herself to you on Wednesday and she was taken into the Home on Wednesday?—No, she never reported herself to me.

*Q.* What day did she go into the Home?—*A.* She went to bed Wednesday evening, and I told her she was not to get up on Thursday morning.

*Q.* Why did you say that?—*A.* She was reported to me on Tuesday morning by Sister Hannah as not feeling very well.

*Q.* Then she was taken with pain on Sunday and spoke to Sister Hannah on Monday?—*A.* Sister Hannah told me on Tuesday when I went round.

*Q.* And on Wednesday night came off duty and kept in bed on Thursday morning?—*A.* Yes.

*Q.* So that the history of the case is right except that she did not report herself to Matron?—*A.* Yes.

Sister Hannah : I first knew of Nurse's abdominal pains on Monday, when we were having some tea and she complained to me.

*Q.* She remained on duty?—*A.* Yes.

*Q.* Did you give her castor oil?—*A.* Not then.

*Q.* When did you report that she was sick to the Matron?—*A.* On the Tuesday.

*Q.* And she stayed on duty till the Wednesday night?—A. Tuesday. Wednesday she had her off day.

*Q.* Then she was on duty half Wednesday?—*A.* Yes.

*Q.* And on Thursday Matron kept her in bed till she had seen the doctor?—*A.* Yes.

Mr. Baker : Do you know if any complaint was made about Miss Scott by her own people?—No.

The Matron : I have had no complaint from them. It was on Tuesday morning when I went round that Nurse Hannah reported her to me, and I said Nurse Scott was to have a half-day out, and I asked the Assistant Matron to look after her as I was going out, and when she went to look after her she found she was out in the town visiting. The half-day out was on Tuesday.

*Q.* Then she was actually on duty the whole of Wednesday?—*A.* Yes; one naturally concluded she was feeling better, and she owned to feeling better on Wednesday night, and after supper asked to be excused, and then it was I said she was to go to bed and not get up next morning.

### DR. EVELYN AND THE RESIDENTS.

Dr. Evelyn : On entering the Residents' room one morning and finding them speaking about malpractices at the Hospital, I said, speaking on my own behalf and on behalf of the staff, "We shall be glad indeed if you will let us know of any really good specific cases of complaint about the administration of the Hospital." "Oh," said Dr. Macqueen, who was with Dr. Shepherd at the time, "we can give you one now." I said, "I shall be very glad if we can have it." He said there was a nurse in the Nurses' Home who had been there four days and had not seen a doctor. Considering that I had seen that nurse for four days in succession, and on two occasions I had come specially to the Hospital to see that nurse, and when they made that remark I had just come from the nurse and had asked the Matron to telephone to Dr. Gostling to come down in the afternoon to see the case, I think you may judge on what flimsy grounds a great number of these allegations are made.

Mr. Baker : Is this the case of Nurse Scott?—Yes, and we are told she had been left without a nurse at all.

The Commissioner : That must have been a singularly bold statement to make to you, the physician to the nursing staff?—I was surprised at it.

Mr. Baker : You had just come from seeing Nurse Scott?—For the fourth time in four days.

*Q.* Where did this interview take place?—*A.* In the Residents' room.

Q. Was anyone else present?—A. Drs. Macqueen and Shepherd.

Q. And Dr. Macqueen said, "We can give you an instance; there is a case upstairs," &c.?—A. Yes.

Q. Those are the very words?—A. Yes.

Q. Did you write them down?—A. No, but they were impressed upon my mind.

The Legal Assessor: What was the matter with the nurse? It has been suggested that it was ordinary stomach ache after eating too much Christmas pudding; is that right?—There was nothing particularly definite detected in her condition, but she complained of abdominal discomfort.

Q. I only want to know why you went four days?

Mr. Baker: Did she ultimately go away on account of this pain; was she ordered away?—We came to the conclusion that she had better have a holiday.

Q. Did you ever find out what was the matter?—A. We never found out absolutely definitely what was the matter. We took the trouble to apply to her doctor to get a report as to her condition and the cause of her previous trouble.

Q. Have you heard nothing more about her since?—A. I have not.

Dr. Macqueen was called and examined by Mr. Baker: You have heard the remark that it has been alleged you made?—Yes.

Q. Do you remember that occasion?—A. I do.

Q. Did you talk about this case?—A. Dr. Evelyn talked about it, and I told him the history of it.

Q. Did he ask you about cases with special regard to the inquiries which were being held?—A. He did.

Q. Do you know what you said in reply?—A. I told him about the case and the history of it; she complained on the Sunday and was never seen till the Thursday, and she was on duty all the time. While I was examining her with Mr. Gostling she had the pain exceedingly severe, and it sometimes doubled her up.

Mr. Gostling called.

Dr. Wilfred Gostling was called and examined by the Commissioner: First about this case of Nurse Scott, have you anything to add to Dr. Evelyn's evidence? I do not feel that I want to go into the medical aspect of the case specially?—I saw her at Dr. Evelyn's request, and we discussed it and subsequently wrote to Mr. Richardson, of Newcastle, who had operated on her, but there was nothing in the operation which would throw any light on the case. It was a perfectly simple appendix operation, not in an acute stage.

### SISTER AND NURSE.

The Commissioner: Now we come to the book-throwing incident.

Dr. Macqueen, examined by Mr. Baker: The names of the two parties concerned in this are Sister Hill and Nurse Ankhurst.

Nurse Ankhurst examined by the Commissioner: In the report in The Hospital it is said that a sister in one of the wards, losing control of herself, threw a book at a nurse in front of the patients. We hear that Sister Hill is the sister concerned, but she is no longer here. What can you tell us about it?—It was quite a mistake that I gave the wrong report to the sister, and she was rather annoyed with me, and lost her temper, and threw the book at me.

Q. What sort of book?—A. An exercise book.

Q. How far did she throw it?—A. I was quite close to her; she did not actually throw it, but slapped my hand with it.

Q. In a painful manner?—A. In a temper.

Q. Was it very painful?—A. No, but I was rather annoyed that she should insult me in front of the patients, and I did not think it right that a sister should lose her temper and throw a book at a nurse. I corrected myself when I gave her the wrong report.

Q. Will you show us how it was done, on Mr. Procter? (Laughter.)—A. I do not know if I can do it the same as she did; she was in a temper, and I am not.

### THE COMMISSIONER CONCLUDES THE PROCEEDINGS.

The Commissioner, in concluding the proceedings, said: I am not proposing to invite either Mr. Procter or Mr. Baker to address me unless they desire. All I can do is to acknowledge the great kindness with which the case has been put before me, not only on one side, but on both. I feel that we are indebted to The Hospital, and, venture to say, to these two gentlemen (Drs. Macqueen and Shepherd) for attending on what must have been a very unpleasant business. We could not have compelled their attendance, and yet without them the inquiry would have been very unsatisfactory, and could not have led to a result which would command confidence. On the other hand, I have to thank the hospital authorities, who, I feel certain, have put before us quite candidly all the information which they have, and I shall use my best judgment to come to an equitable conclusion on the matters submitted to me.

